# The first dimeric indole-diterpenoids from a marine-derived *Penicillium* sp. fungus and their potential for anti-obesity drugs

**DOI:** 10.1007/s42995-024-00253-x

**Published:** 2024-10-08

**Authors:** Hui-Fang Du, Lei Li, Ya-Hui Zhang, Xu Wang, Cheng-Yan Zhou, Hua-Jie Zhu, Charles U. Pittman, Jia-Wen Shou, Fei Cao

**Affiliations:** 1 College of Pharmaceutical Sciences, Key Laboratory of Medicinal Chemistry and Molecular Diagnostics of Education Ministry of China, Key Laboratory of Pharmaceutical Quality Control of Hebei Province, Hebei University, Baoding, 071002 China; 2 School of Chemistry and Pharmaceutical Engineering, Hebei University of Science and Technology, Shijiazhuang, 050018 China; 3 Department of Chemistry, Mississippi State University, Mississippi State, MS 39762 USA; 4 School of Life Sciences, The Chinese University of Hong Kong, Hong Kong, China

**Keywords:** Marine-derived fungus, *Penicillium* sp., Dimeric indole-diterpenoid, Absolute configuration, 3T3-L1 cell

## Abstract

Obesity has become a worldwide health problem. Seeking natural products with anti-obesity activity from lots of fungi has drawn the attention of pharmacologists. In our study, dipenipenoids A and B (**1** and **2**), the first dimeric indole-diterpenoids with a rare C-20–C-22′ linkage, and their monomers (**3** and **4**), were isolated from a marine-derived *Penicillium* sp. CF-06 fungus from *Suaeda salsa*. The absolute configurations of **1**–**3** were assigned by the calculated TDDFT ECD method. The structure of **4** was verified by a single-crystal X-ray diffraction method for the first time. Interestingly, **1** and** 2** displayed significant effects on the differentiation of 3T3-L1 adipocytes by down-regulating the expression of peroxisome proliferator-activated receptor gamma (PPAR*γ*) and CCAAT/enhancer binding protein alpha (C/EBP*α*) proteins, while monomers **3** and **4** exhibited no activity. Molecular docking results explained the mechanism that the interaction between dimer **1** and PPAR*γ* was stronger than that between monomer **3** and PPAR*γ*. Our research could provide new insight for the discovery of anti-obesity drugs.

**Supplementary Information:**

The online version contains supplementary material available at 10.1007/s42995-024-00253-x.

## Introduction

Nutrient excess over energy expenditure fuels obesity, driving adipocyte proliferation and enlargement, culminating in abnormal fat deposition (Kalra et al. 2021). Obesity, paralleling HIV/AIDS, substance misuse, and alcoholism in global health impact (Chooi et al. 2019), disrupts glucose and lipid homeostasis, expanding adipose tissue and causing ectopic lipid build-up, instigating lipid metabolism disorders and hyperlipidemia. It triggers insulin resistance, impairing glucose handling in vital organs, escalating blood glucose and type 2 diabetes mellitus risk (Scully et al. 2021), while exacerbating comorbidities like atherosclerosis and non-alcoholic fatty liver disease, heightening health risks and mortality (Chapman and Sposito 2008). Simple obesity, accounting for roughly 95% of cases, stems predominantly from poor lifestyle, including unhealthy diets and inactivity (Alsulami et al. 2023).

Treatment strategies encompass physical activity, dietary modification, pharmacotherapy, and bariatric surgery (Ruban et al. 2019), targeting caloric deficits. Lifestyle changes require sustained effort, and surgery, while effective, is reserved for severe cases due to risks (Mann et al. 2007). Pharmacotherapies are prevalent (Sweeting et al. 2015), divided into metabolic stimulators and fat absorption blockers. Metformin enhances metabolism and insulin sensitivity but lacks obesity treatment approval (Yerevanian and Soukas 2019), while orlistat reduces weight by inhibiting gut fat absorption, albeit with side effects (Ballinger and Peikin 2002). Other agents, like laxatives and traditional Chinese medicines, limit fat absorption but have limitations (Sui et al. 2012). Hence, identifying new natural substances that enhance glucose and lipid metabolism is critical for public health.

**Fig. 1:**
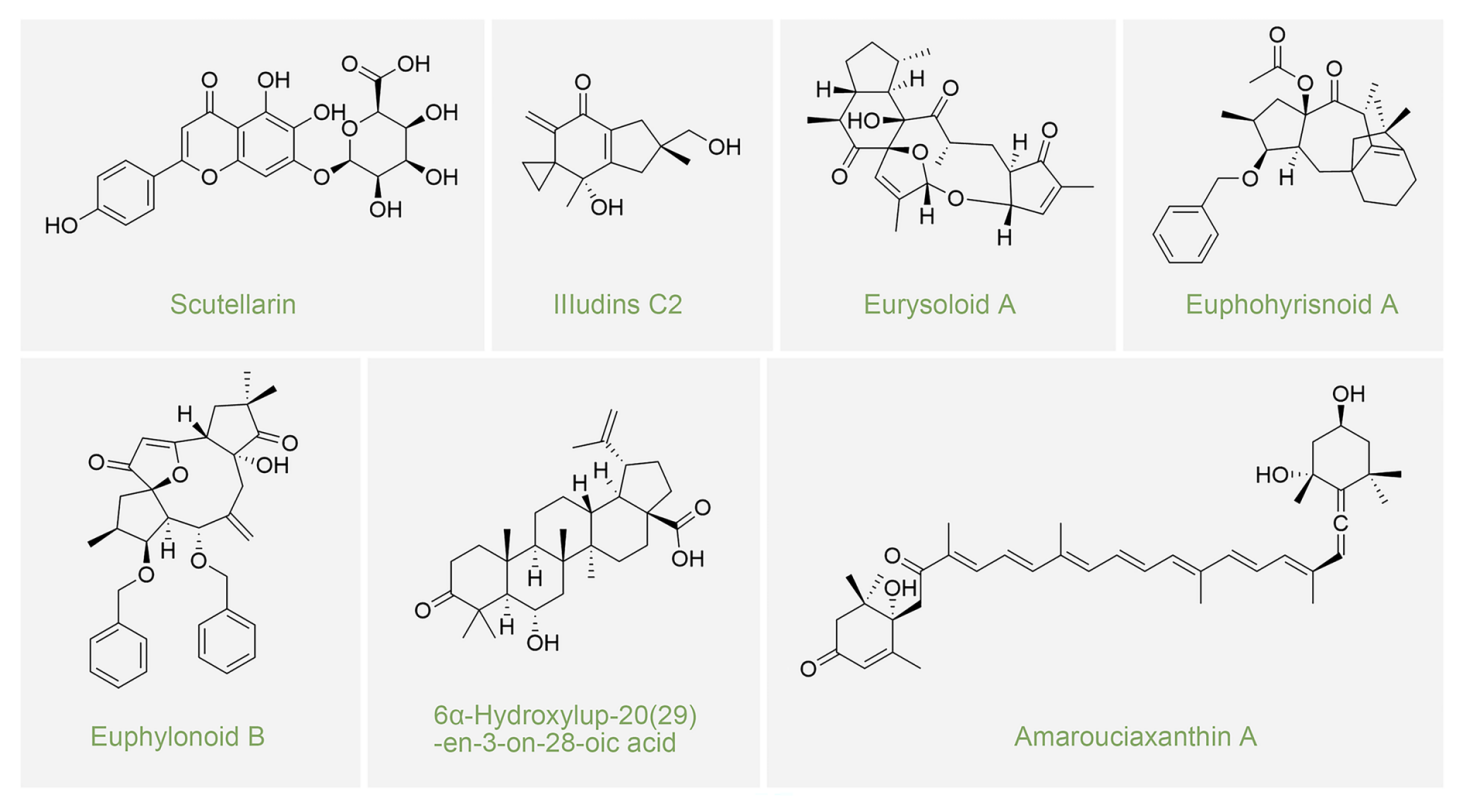
Natural products with inhibitory activity on adipocyte differentiation

Regulation of adipose differentiation is pivotal in obesity prevention. Research has concentrated on natural compounds from plants and endophytic fungi, known for their distinct structures and effects on adipocyte differentiation (Fig. 1) (Cheng et al. 2020; Choi et al. 2014; Haaz et al. 2006; Wang et al. 2022a, b). Alkaloids, polyphenols, and terpenoids show anti-obesity potential by controlling adipogenesis (Wang et al. 2022a, b). Certain alkaloids suppress adipocyte differentiation and lipid accumulation in 3T3-L1 cells (Choi et al. 2014). The genus *Suaeda*, global halophytes, is credited in Chinese folklore for weight loss and hypoglycemic properties, and their early shoots are consumed as greens in mice studies without adverse effects (Chen et al. 2020; Sun et al. 2007). Extensive research reveals bioactive compounds, including alkaloids, terpenoids, and phenylpropanoids from *Suaeda* species, are promising for drug development (Wang et al. 2022a, b). Yet, evidence suggests plant microbes may also produce certain bioactive compounds (Kelecom 2022).

**Fig. 2:**
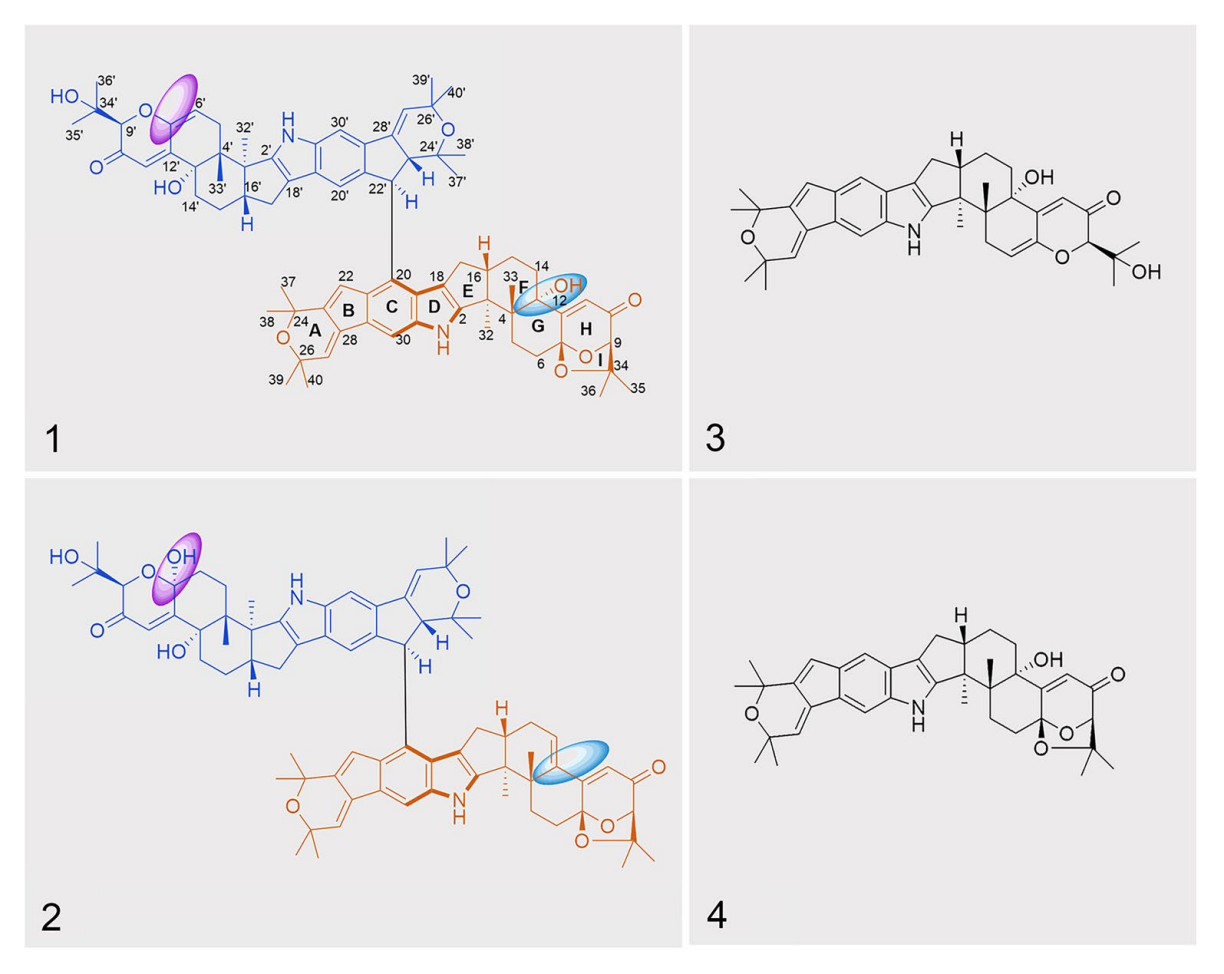
Chemical structures of **1**–**4** from fungal strain *Penicillium* sp. CF-06

In our research of marine natural products from fungi (Cao et al. 2019a, b; Cao et al. 2019a, b; Zhang et al. 2022), *Penicillium* sp. CF-06, isolated from *S. salsa*, showed effects on 3T3-L1 adipocyte differentiation. Bioassay-guided isolation resulted in novel dimeric indole-diterpenoids (Fig. 2) impacting 3T3-L1 adipogenesis. This report details the isolation, structural elucidation, and bioactivities of these indole-diterpenoids.

## Materials and methods

### Experimental Section

#### General experimental procedures

OR data were recorded in CH_3_OH on a JASCO P-2000 spectrometer. UV spectra were recorded on a Shimadzu UV-3600 spectrometer. Electronic circular dichroism (ECD) spectra were acquired on a MOS-450/SFM300 spectrometer. NMR data were measured using a Bruker Avance-III 600 MHz NMR spectrometer. HRESIMS spectra were acquired using a Thermo Scientific LTQ Orbitrap XL spectrometer. Crystallographic data (Cu K*α* radiation) was obtained from a Bruker D8 ADVANCE diffractometer. HPLC separation was done using a Shimadzu LC-6AD system with SPD-20A photodiode array detector and a Waters C_18_ HPLC column (10 mm × 250 mm, 5 µm). Column chromatography was carried out with Sephadex LH-20 18 − 110 µm and Silica gel 200–300 mesh.

#### Fungal material and isolation

The strain *Penicillium* sp. CF-06 was isolated from a piece of fresh tissue obtained from the inner part of *Suaeda salsa* at the seedling stage, identified by Prof. Hongliang Tang, which was collected from the intertidal zone of the Bohai Sea, Huanghua, China, in July 2019. Identification of the strain CF-06 was done according to a molecular biological protocol by amplification and sequencing of the ITS region. The sequence of this strain showed a 99.43% identity with *Penicillium solitum* (GenBank No. MK761050), *P. echinulatum* (GenBank No. KT876710), *P. commune* (GenBank No. MT626053), and *P. discolor* (GenBank No. LR758010). Based on the above blast analysis, the strain CF-06 was identified as *Penicillium* sp. and preserved in the College of Pharmaceutical Sciences, Hebei University, China. The sequence data had been submitted to GenBank with accession number OQ891231. The strain was fermented in optimized PDB media (containing 3.3% CaCl_2_) at 28 °C for 30 days. Each Erlenmeyer flask (1L) contained 400 mL PDB, totaling 20 flasks. The fermentation broth and mycelia were extracted with EtOAc and 95% methanol, respectively, to yield the crude extract. H_2_O and EtOAc were used to partition the crude extract, and the organic phase was concentrated to gain the EtOAc extract (3.2 g). This extract was loaded on silica gel column chromatography (CC) and eluted with an increasing gradient of petroleum ether (PE)/EtOAc (90:10, 50:50, 10:90) to afford the fractions of Fr.1–Fr.3. Among them, Fr.2 was subjected to gel permeation chromatography on Sephadex LH-20, eluted with CH_2_Cl_2_-MeOH (1:1), to give two subfractions Fr.2.1 and Fr.2.2. Among them, Fr.2.1 was purified using silica gel chromatography and further separated by semipreparative HPLC on a Waters C_18_ column using MeOH-H_2_O (80:20, v/v, 2.0 mL/min) to obtain compounds **1** (6.2 mg, 15.2 min) and **2** (5.3 mg, 18.9 min). Fr.2.2 was first fractionated by silica gel chromatography (stepped gradient, EtOAc-PE) and then applied to semipreparative HPLC eluting with 80% MeOH-H_2_O (2.0 mL/min) to give compounds **3** (4.6 mg, 10.4 min) and **4** (10.9 mg, 22.6 min).

*Dipenipenoid A (****1****)*: pale-yellow powder. [*α*]_D_^25^ – 42.3 (*c* 0.10, MeOH); UV (MeOH), λ_max_ (log *ε*) 232 (4.42), 286 (4.98), 385 (0.93) nm; ECD (0.20 mmol/L, MeOH), λ_max_ (Δ*ε*) 232 (– 3.68), 257 (0.83), 294 (– 1.76), 330 (5.93) nm; ^1^H and ^13^C NMR data, see Table S1. HRESIMS *m/z*: 1161.6204 [M − H]^−^, (C_74_H_86_N_2_O_10_, calcd. for 1161.6210).

*Dipenipenoid B (****2****)*: pale-yellow powder. [*α*]_D_^25^ – 13.5 (*c* 0.10, MeOH); UV (MeOH), λ_max_ (log *ε*) 233 (4.10), 284 (5.09), 383 (0.97) nm; ECD (0.20 mmol/L, MeOH), λ_max_ (Δ*ε*) 233 (– 8.96), 249 (0.74), 297 (– 10.62), 349 (2.35) nm; ^1^H and ^13^C NMR data, see Table S2. HRESIMS *m/z*: 1161.6204 [M − H]^−^, (C_74_H_86_N_2_O_10_, calcd. for 1161.6210).

*Penipenoid A (****3****)*: pale-yellow powder. [*α*]_D_^25^ – 6.2 (*c* 0.10, MeOH); UV (MeOH), λ_max_ (log *ε*) 232 (2.81) 286 (3.34), 372 (0.90) nm; ECD (0.20 mmol/L, MeOH), λ_max_ (Δ*ε*) 234 (2.14), 259 (2.05), 334 (3.06); ^1^H and ^13^C NMR data, see Table S3. HRESIMS *m/z*: 580.3065 [M–H]^–^, (C_37_H_43_NO_5_, calcd. for 580.3068).

Crystallographic date for 22,23-dehydro-shearinine A (**4**): C_37_H_43_NO_5_, F.W. = 581.72, colorless block, monoclinic, space group *P*21, *a* = 7.2877(1)Å, *b* = 10.2472(1)Å, *c* = 21.7903(3)Å, *α* = 90°, *β* = 98.319°, *γ* = 90°, *V* = 1610.15(13)Å^3^, Z = 2, Dc = 1.2 mg/cm^3^, *μ*(Cu, K*α*) = 0.627 mm^−1^, *F*(000) = 624.0. Crystal dimensions: 0.13 mm × 0.13 mm × 0.13 mm. A total of 6598 unique reflections [R(int) = 0.0234 (inf-0.9 Å)], which were used in all calculations; the final refinement gave R1 = 0.0444 (*I* > 2σ(*I*)) and wR2 = 0.1116 (all data), and S = 1.051; Flack parameter = 0.0(2). CDCC number: 2258497.

#### Computational section

The MMFF94 force field by the ComputeVOA software was used to searching conformers of the molecules 3*S*,4*R*,7*S*,9*R*,13*S*,16*S*,3'*S*,4'*R*,9'*R*,13'*S*,16'*S*,22'*R*,23'*R* (**1a**), 3*S*,4*R*,7*S*,9*R*,13*S*,16*S*,3'*S*,4'*R*,9'*R*,13'*S*,16'*S*,22'*S*,23'*S* (**1b**), 3*S*,4*S*,7*S*,9*R*,16*S*,3'*S*,4'*R*,7'*S*,9'*R*,13'*S*,16'*S*,22'*R*,23'*R* (**2a**), 3*S*,4*S*,7*S*,9*R*,16*S*,3'*S*,4'*R*,7'*S*,9'*R*,13'*S*,16'*S*,22'*S*,23'*S* (**2b**), and 3*S*,4*R*,9*R*,13*S*,16*S* (**3**), and the enantiomers of them (3*R*,4*S*,7*R*,9*S*,13*R*,16*R*,3'*R*,4'*S*,9'*S*,13'*R*,16'*R*,22'*S*,23'*S* (*ent*-**1a**), 3*R*,4*S*,7*R*,9*S*,13*R*,16*R*,3'*R*,4'*S*,9'*S*,13'*R*,16'*R*,22'*R*,23'*R* (*ent*-**1b**), 3*R*,4*R*,7*R*,9*S*,16*R*,3'*R*,4'*S*,7'*R*,9'*S*,13'*R*,16'*R*,22'*S*,23'*S* (*ent*-**2a**), 3*R*,4*R*,7*R*,9*S*,16*R*,3'*R*,4'*S*,7'*R*,9'*S*,13'*R*,16'*R*,22'*R*,23'*R* (*ent*-**2b**), and 3*R*,4*S*,9*S*,13*R*,16*R* (*ent*-**3**)). A total of 4 stable conformers for **1a** with relative energy within a 10.0 kcal/mol energy window, 4 conformers for *ent*-**1a**, 2 conformers for **1b**, 2 conformers for *ent*-**1b**, 4 conformers for **2a**, 4 conformers for *ent*-**2a**, 2 conformers for **2b**, 2 conformers for *ent*-**2b**, 5 conformers for **3**, and 5 conformers for *ent*-**3** were recorded. Geometries were optimized at the level of B3LYP/6-31G(d) using density functional theory (DFT) calculations. The optimized geometries to be minima (no imaginary frequency) or transition states (TSs, having unique one imaginary frequency) were verified by harmonic frequency analysis. The ECD calculations for the stable conformers were carried out at the B3LYP/6-31G(d) level in the gas phase using Gaussian 09 software (Frisch et al. 2009). Boltzmann statistics were used to combine ECD spectra using SpecDis 1.64 software (Bruhn et al. 2013).

#### Cytotoxicity assay and oil red-O staining

The murine 3T3-L1 cells (ATCC) were grown using DMEM (Gibco) with 10% fetal bovine serum (FBS, Gibco). The cytotoxicity of **1**–**4** and berberine to 3T3-L1 cells was determined by the MTT method (Mosmann 1983). For Oil Red O staining, 3T3-L1 cells, which were grown in 6-well plates to full confluence for 2 days, were stimulated using DMEM/10% FBS culture medium with 0.5 mmol/L dexamethasone (Sigma), 0.5 mmol/L 3-isobutyl-1-methylxanthine (Sigma), and 10 mg/mL insulin (Sigma) for 72 h. After the induction, the medium was replaced by DMEM with 10% FBS for differentiation at 37 °C and 5% CO_2_. The tested compounds of **1**–**4** and berberine were dissolved in DMEM and added to the 6-well plates at the indicated concentration. After spawning, cells were twice washed using PBS and fixed for 30 min with 10% glutaraldehyde. Subsequently, the cells were stained with 0.5% Oil Red-O (Sigma) for 30 min at room temperature (rt). PBS was used to wash the excess Oil Red-O dye. Pictures were taken using an OX.2003 (Euromex) microscope. Isopropanol was used to extract the stained oil droplets in 3T3-L1 cells and then to measure the absorbance at 490 nm (Huang et al. 2006). Berberine was used as a positive control, which was reported as a peroxisome proliferator-activated receptor gamma (PPAR*γ*) inhibitor in a previous study (Huang et al. 2006; Shou and Shaw 2023). Berberine was not cytotoxic at concentrations less than 100.0 μmol/L to 3T3-L1 cells after incubation for 120 h. Berberine could reduce the Oil Red-O staining level of 3T3-L1 cells to 90.9%, 85.0%, 69.8%, and 64.5% at the concentrations of 6.8, 13.5, 27.0, and 53.9 μmol/L, respectively, when compared with the control group.

#### Western blot analysis

The murine 3T3-L1 cells were lysed with cold RIPA buffer (Thermo Fisher) and boiled for five minutes to give total protein. The protein (30 μg/lane) was separated by 10% SDS–PAGE, which was then transferred to a PVDF membrane and blocked using 5% bovine serum albumin (BSA, Sigma) for 2 h at rt. The membrane was incubated with antibody against PPAR*γ* or CCAAT/enhancer binding protein alpha (C/EBP*α*) (Cell Signaling Technology) for 12 h at 4 °C and a secondary antibody of rabbit IgG-conjugated horseradish peroxidase (Cell Signaling Technology) for 6 h at 4 °C. *β*-Actin was used as the control protein (Maeda et al. 2006).

### Molecular docking

PPAR*γ* protein structure (pdb code: 2prg) (Nolte et al. 1998) was used to verify the potential binding site by Autodock 4.2 software (Rizvi et al. 2013). Grid box with size x: 58 Å; y: 68 Å; z: 70 Å, spacing of 0.481 Å, was set in the center of the active site (55.481, –– 41.359, 10.732). Docking results were scored according to the binding free energy. The best fit and top-scoring conformations of compounds and PPAR*γ* were chosen for visualization by using Pymol software.

### Statistical analysis

All the experimental data were obtained from three independent experiments. Statistical significance of differences was analyzed using with one-way analysis of variance (ANOVA), followed by Duncan’s multiple range test. The results were considered to be significant for *p* < 0.05.

## Results and discussion

**Fig. 3:**
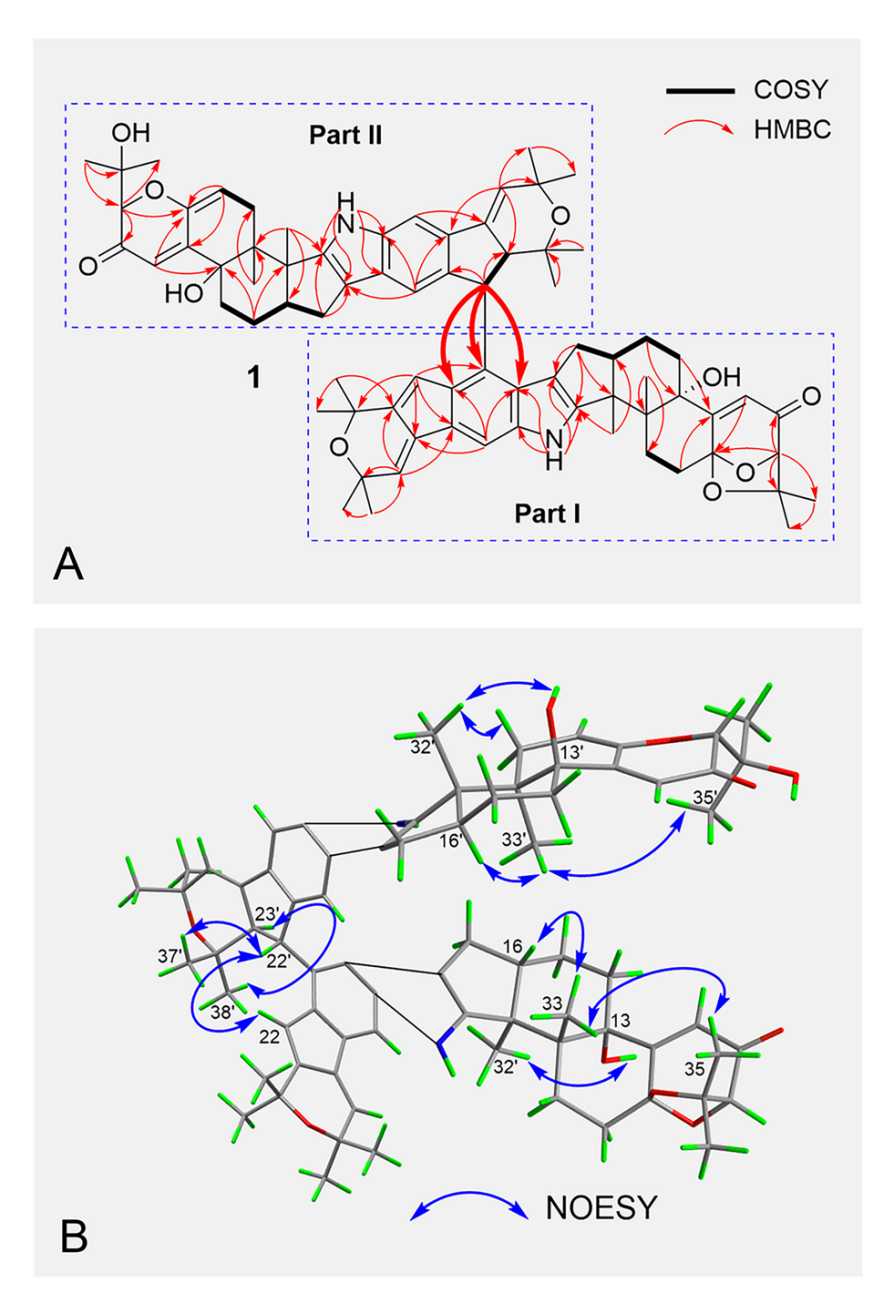
COSY, Key HMBC and Key NOESY correlations of **1**. **A** COSY and Key HMBC correlations of **1**. **B** Key NOESY correlations of **1**

Dipenipenoid A (**1**) was obtained as a pale-yellow powder. The molecular formula C_74_H_86_N_2_O_10_ was determined by the prominent pseudomolecular ion peaks at *m*/*z* 1161.6204 [M − H]^−^ in the HRESIMS spectrum, which could account for 33 degrees of unsaturation of **1**. The NMR data (Table S1), which showed thirty-four C signals, fifteen CH signals, nine CH_2_ signals, and sixteen CH_3_ signals, could be ascribed to two groups (eighteen C, six CH, five CH_2_, and eight CH_3_ *vs.* sixteen C, nine CH, four CH_2_, and eight CH_3_), suggesting that **1** is a dimeric product and consists of two similar units (parts **I** and **II**) (Fig. 2). Detailed analysis of the ^1^H NMR data of part **I**, the characteristic NH signal at *δ*_H_ 7.62, singlet aromatic proton signal at *δ*_H_ 7.40, multiple overlapped CH_2_ signals at *δ*_H_ between 2.79 and 0.63, and eight CH_3_ signals at *δ*_H_ 1.58, 1.56, 1.50, 1.50, 1.44, 1.33, 1.19, and 1.17, suggested a shearinine type indole-diterpenoid skeleton (Belofsky et al. 1995; Kong et al. 2019) for part **I**. Careful comparison of the ^1^H and ^13^C NMR data of part **I** with those of 22,23-dehydro-shearinine A (**4**) (You et al. 2013) revealed part **I** shares the same nine rings system (rings A-I) as **4**. The main difference between part **I** and compound **4** was that the H-20 aromatic proton signal (*δ*_H_ 7.21) present in **4** was absent in part **I**, suggesting the C-20 in part** I** should be connected to part **II**. Then, the remaining unit (part **II**) of **1** was also assigned to a shearinine-type indole-diterpenoid derivative. According to their ^1^H and ^13^C NMR data, there were two main differences between parts **II** and **I**. One of the differences was that the C-6 methene and C-7 acetal tertiary carbon [*δ*_H_ 2.79 (dt, 10.8, 3.6 Hz, H-6a) and 2.05 (m, H-6b);* δ*_C_ 104.5 (C-7) and 28.4 (C-6)] in part **I** was replaced by one double bond [*δ*_H_ 5.72 (d, 6.6 Hz, H-6'); *δ*_C_ 145.0 (C-7') and 111.6 (C-6)'] in part **II**, indicating that the ring in part **I** was disconnected from C-7 and C-34 to form the double bond between C-6' and C-7' in part **II**. This deduction was confirmed by the HMBC correlations from H-6' to C-7'/C-12' and from H-9'/H-11' to C-7' (Fig. 3A). The other difference was that the double bond between C-22 and C-23 [*δ*_H_ 6.60 (s, H-22); *δ*_C_ 142.0 (C-23) and 119.7 (C-22)] in part **I** was replaced by a -CHCH- group [*δ*_H_ 4.60 (d, 7.8 Hz, H-22') and 3.37 (dd, 7.8, 3.0 Hz, H-23'); *δ*_C_ 57.4 (C-23') and 47.7 (C-22')] in part **II**, which was confirmed by the COSY cross-peaks of H-22'/H-23' and the HMBC correlations from H-22'/H-27' to C-23'. Subsequently, two monomeric indole-diterpenoid derivatives (parts **I** and **II**) were connected via the new C−C bond between C-20 and C-22', which was suggested by the key HMBC correlations from H-22' to C-19/C-20/C-21. Thus, the planar structure of **1** was established.

To assign the relative configuration, NOESY data of **1** was measured (Fig. 3B). In part **I**, NOESY showed the correlations from H_3_-33 to H-16/H_3_-35 and from H_3_-32 to 13-OH. Meanwhile, in part **II**, NOESY displayed the correlations from H_3_-33' to H-16'/H_3_-35' and from H_3_-32' to 13'-OH. The above NOESY correlations indicated that both parts **I** and **II** had the same *β*-orientations of H-16, CH_3_-33, H-16', and CH_3_-33' and the same *α*-orientations of 13-OH, H-9, CH_3_-32, 13'-OH, H-9', and CH_3_-32' as those of **4**. Moreover, the NOE relationships between H-22' and H-37', and H-23' and H-38', gave the relative configurations of H-22' and H-23' of part **II** in **1**.

**Fig. 4:**
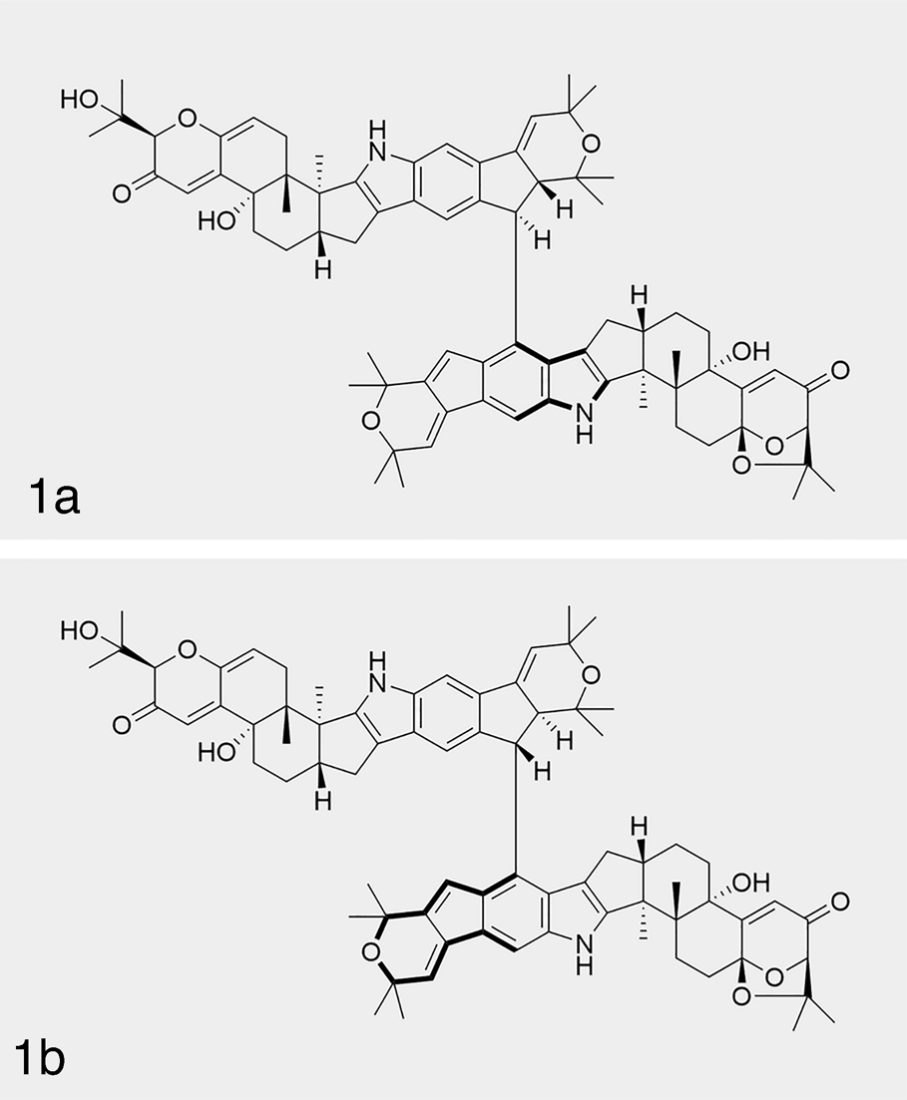
The possible configurations (**1a** and **1b**) of **1**

**Fig. 5:**
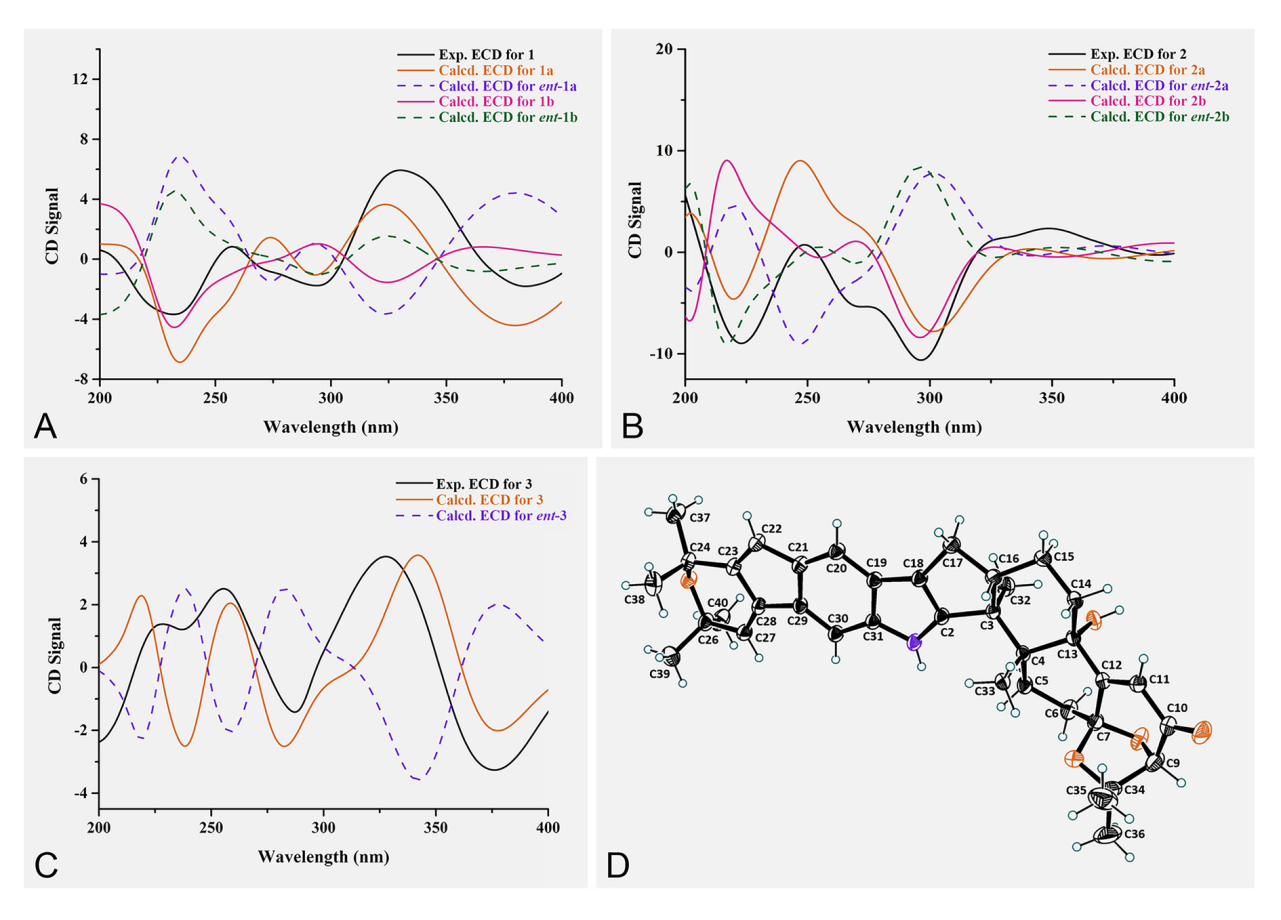
Experimental and calculated ECD spectra of **1‒3** and X-ray structure of **4**. **A** Experimental and calculated ECD spectra of **1**. **B** Experimental and calculated ECD spectra of **2**. **C** Experimental and calculated ECD spectra of **3**. **D** X-ray structure of **4**

With high steric hindrance from two diterpene portions between parts **I** and **II**, compound **1** might possess axial chirality. Conformational search was conducted for the molecule of (3*S*,4*R*,7*S*,9*R*,13*S*,16*S*,3'*S*,4'*R*,9'*R*,13'*S*,16'*S*,22'*R*,23'*R*)-**1** to explore whether the *M* and *P* conformations were tautomeric. The relative Gibbs energy barrier (ΔG) for *M*–*P* conversion is 25.0 kcal/mol, which confirmed the existence of stable *M* or *P* isomers at rt (Fig. S58). The key NOESY correlation between H-22 and H-22' could be used to determine the relative relationship between axial chirality and stereogenic carbons of C-22' and C-23' (M*,22'R*,23'R* or P*,22'S*,23'S*). To assign the absolute configuration of **1**, four possible configurations [*M*, 3*S*,4*R*,7*S*,9*R*,13*S*,16*S*,3'*S*,4'*R*,9'*R*,13'*S*,16'*S*,22'*R*,23'*R* (**1a**), *P*,3*S*,4*R*,7*S*,9*R*,13*S*,16*S*,3'*S*,4'*R*,9'*R*,13'*S*,16'*S*,22'*S*,23'*S* (**1b**), and their enantiomers (*ent*-**1a** and *ent*-**1b**)] of **1** (Fig. 4) were calculated using their ECD spectra and the quantum chemical TDDFT method. As a result, the computational ECD spectrum for **1a** gave better agreement with the experimental data of **1** (Fig. 5A). Thus, the absolute configuration of **1** could be determined as *M*,3*S*,4*R*,7*S*,9*R*,13*S*,16*S*,3'*S*,4'*R*,9'*R*,13'*S*,16'*S*,22'*R*,23'*R*.

Dipenipenoid B (**2**) was also isolated as a dimeric indole-diterpenoid derivative with the same molecular formula C_74_H_86_N_2_O_10_ as **1**. By careful comparison the 1D NMR data of **1** and **2** (Table S2), the main differences were that the 13-OH (*δ*_H_ 3.23) in **1** was dehydrated to form the 13-ene moiety [*δ*_H_ 5.99 (m, H-14); *δ*_C_ 140.7 (C-13) and 132.3 (C-14)] in **2**, while the trisubstituted double bond between C-6' and C-7' in **1** was hydrated to form a C-7 hemiacetal tertiary carbon (*δ*_C_ 94.4) in **2**. This observation was confirmed by the COSY correlations between H-14 and H_2_-15 and the HMBC correlations from H-11/H_2_-15 to C-13 and from H_2_-6'/H-9'/H-11' to C-7' (Fig. S1).

Analysis of the NOESY spectra of **1** and **2** revealed that they showed similar NOE correlations, suggesting that **2** had the similar relative configuration as **1**. The relative configurations of 7'-OH and 13'-OH were assigned by the NOE correlations between 7'-OH and 13'-OH and between 13'-OH and H-32'. In addition, four possible configurations [*M*, 3*S*,4*S*,7*S*,9*R*,16*S*,3'*S*,4'*R*,7'*S*,9'*R*,13'*S*,16'*S*,22'*R*,23'*R* (**2a**), *P*, 3*S*,4*S*,7*S*,9*R*,16*S*,3'*S*,4'*R*,7'*S*,9'*R*,13'*S*,16'*S*,22'*S*,23'*S* (**2b**), and their enantiomers (*ent*-**2a** and *ent*-**2b**)] (Fig. S2) were used for ECD calculations to assign the absolute configuration of **2**. The results showed that the calculated ECD curve of **2a** was consistent with the experimental ECD curve of **2** (Fig. 5B). Thus, the structure of **2** could be elucidated as (*M*, 3*S*,4*S*,7*S*,9*R*,16*S*,3'*S*,4'*R*,7'*S*,9'*R*,13'*S*,16'*S*,22'*R*,23'*R*)-dipenipenoid B.

Penipenoid A (**3**) was obtained as a pale-yellow powder. According to the molecular formula C_37_H_43_NO_5_, combined with 1D NMR data (Table S3), it could be deduced that **3** was a monomeric indole-diterpenoid derivative. In fact, the planar structure of **3** was the precursor of part **II** in **1**, which could be further verified by detailed analysis of its 2D NMR spectra (Fig. S3). Subsequently, the NOESY experiment (Fig. S4) and calculated ECD method (Fig. 5C) were carried out to determine the relative and absolute configuration of **3**, respectively, which was the same as those of part **II** in **1** (3*S*,4*R*,9*R*,13*S*,16*S*).

In addition to the above **1**–**3**, the known 22,23-dehydro-shearinine A (**4**) was also co-isolated, whose structure was further identified by a single-crystal X-ray diffraction method for the first time (Fig. 5D). From the structural analysis, it was the precursor of part **I** in **1**.

Subsequently, the effects of **1**–**4** on the differentiation of murine 3T3-L1 adipocyte cells were investigated. The viability of 3T3-L1 cells treated with **1**–**4** was first measured by the MTT assay. It was found that compounds **1**–**4** were not cytotoxic at concentrations less than 50.0 μmol/L to 3T3-L1 cells after incubation for 120 h. The effects of **1**–**4** on lipid accumulation during 3T3-L1 cell differentiation were then tested using Oil Red-O dye staining, which could indicate intercellular lipid accumulation in 3T3-L1 cells.

**Fig. 6:**
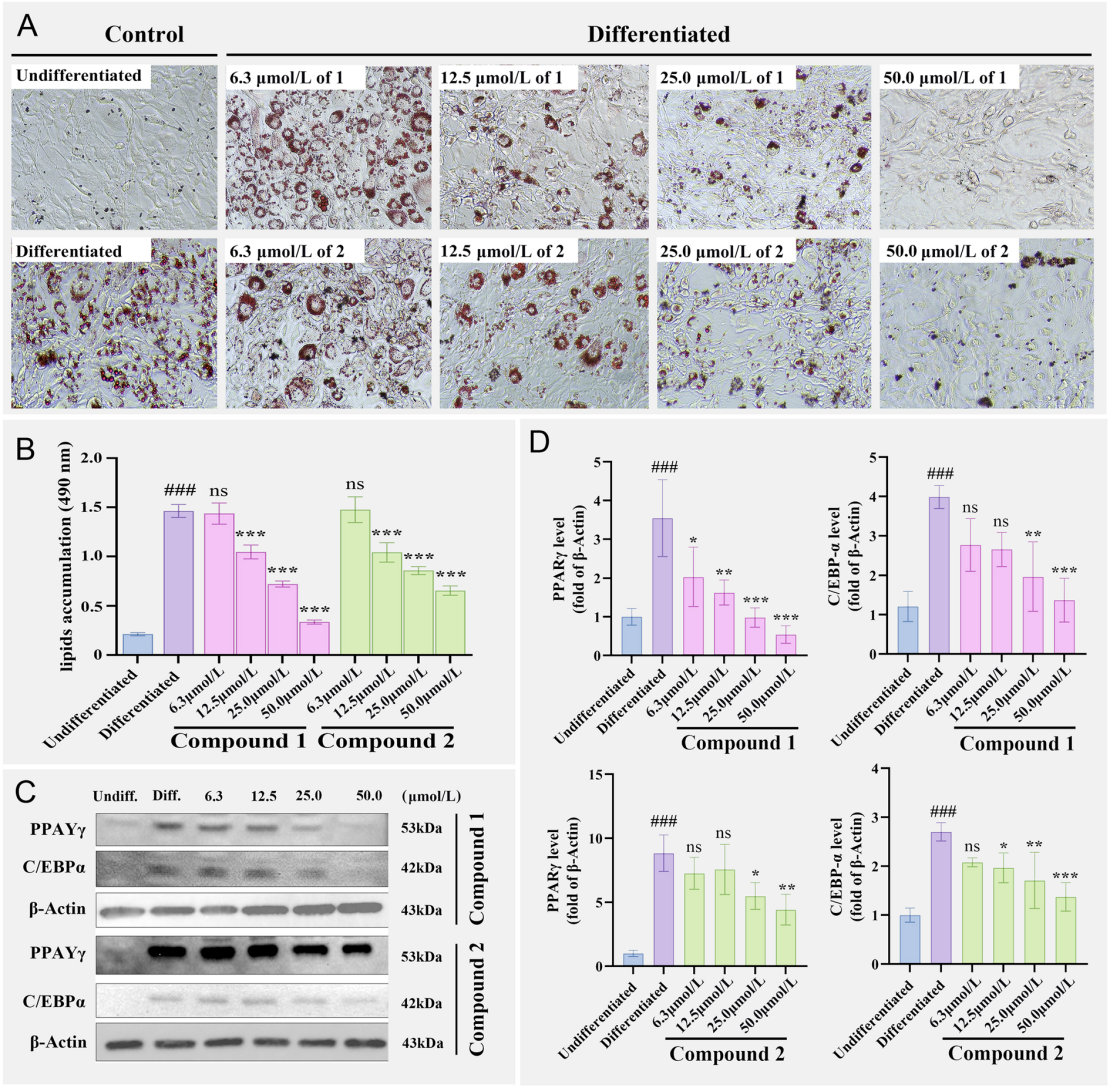
Effects of **1** and **2** on the differentiation of 3T3-L1 cells. **A** 3T3-L1 adipocytes were treated with **1** and **2** at 6.3, 12.5, 25.0, and 50.0 μmol/L, harvested after the initiation of differentiation, and stained with Oil Red-O. **B** Quantification of lipid accumulation by Oil Red-O staining at 490 nm. **C** Protein expression of PPAR*γ* and C/EBP*α* in 3T3-L1 cells treated with **1** and **2** at 6.3, 12.5, 25.0, and 50.0 μmol/L. **D** Quantification of PPAR*γ* and C/EBP*α* according to the reference bands of *β*-actin*.* The data represent the mean ± SEM for three triplicates. ^###^*P* < 0.001 vs undifferentiated group, ***P < 0.001, **P < 0.01, *P < 0.05, ns, no significance. *vs* differentiated group

Compared with the positive control berberine, the dimers (**1** and **2**) showed better inhibition of lipid accumulation in 3T3-L1 cells. Compounds **1** and **2** could significantly attenuate the Oil Red-O staining level of 3T3-L1 cells with IC_50_ values of 19.5 and 28.1 μmol/L, respectively. Particularly, compound **1** could reduce the Oil Red-O staining level to 9.9%, which was almost completely suppressed (Fig. 6A, B). To investigate the mechanisms underlying **1** and **2** suppression of adipocyte differentiation, the expression of PPAR*γ* and C/EBP*α* proteins, which are known as key station proteins expressed in the adipocyte differentiation of 3T3-L1 cells, was analyzed by Western blotting. It was found that PPAR*γ* and C/EBP*α* were markedly reduced during adipocyte differentiation. It was noteworthy that PPAR*γ* and C/EBP*α* levels were down-regulated in 3T3-L1 cells treated with **1** and **2** after incubation (Fig. 6C, D). At the concentration of 6.3, 12.5, 25.0, and 50.0 μmol/L of **1**, the PPAR*γ* expression level decreased to 57.2%, 45.9%, 27.7%, and 15.2% that of control cells differentiated, respectively. While, at the concentration of 25.0 and 50.0 μmol/L of **2**, the PPAR*γ* expression level decreased to 62.2% and 50.1% that of control cells differentiated, respectively.

**Fig.7:**
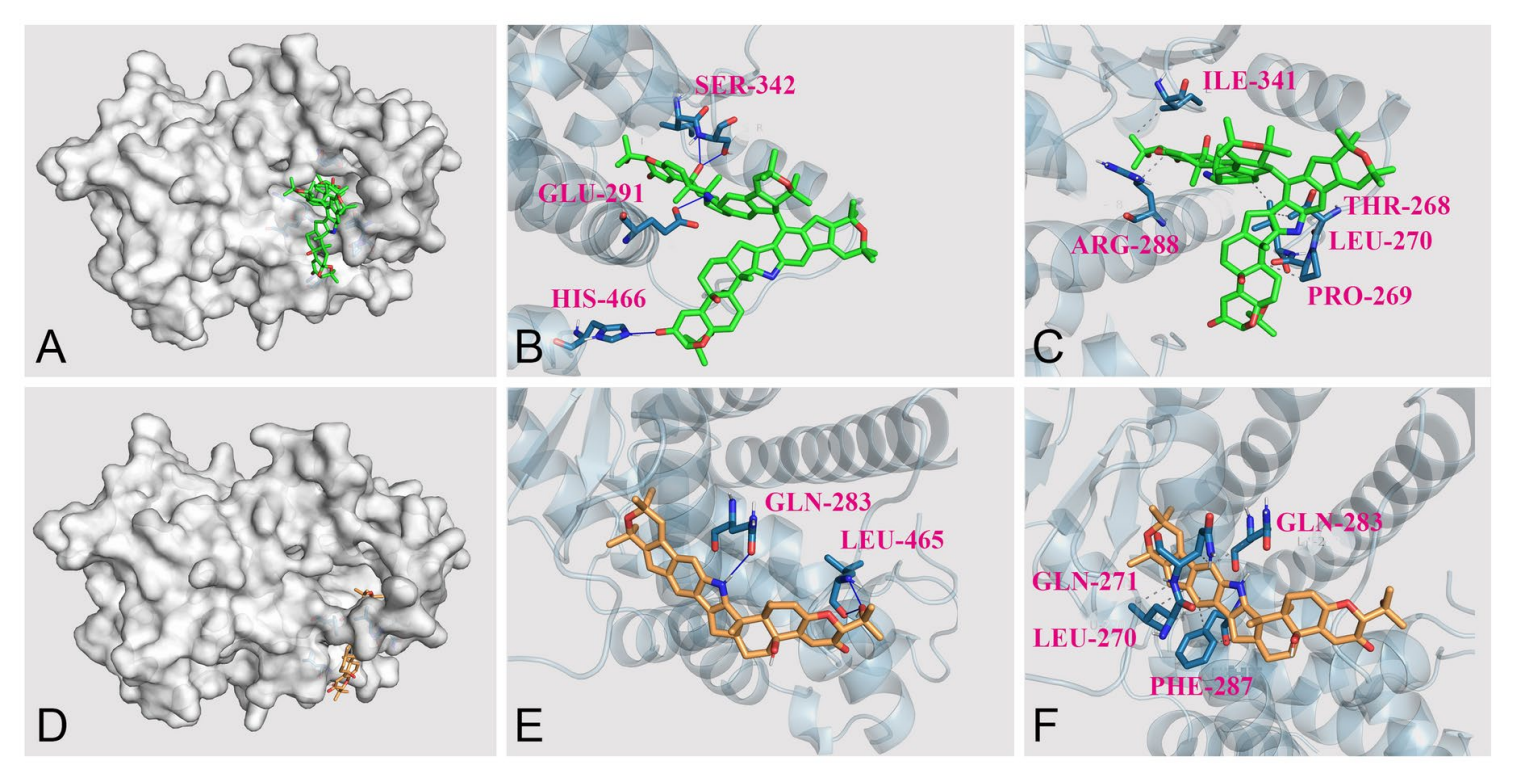
Docking results of PPAR*γ* (PDB: 2prg) with compounds via AutodockTools. **A** Docking simulations of **1** (colored in green) with PPAR*γ*. **B** H-bonds between **1** and protein PPAR*γ*. **C** Hydrophobic interactions between **1** and protein PPAR*γ*. **D** Docking simulations of **1** (colored in orange) with PPAR*γ*. **E** H-bonds between **1** and protein PPAR*γ*. **F** Hydrophobic interactions between **1** and protein PPAR*γ*

It was interesting that the dimeric indole-diterpenoids **1** and **2** showed effects on 3T3-L1 adipogenesis, but their monomers **3** and **4** exhibited no activity. To further explore the differences of effects between dimers and their monomers on 3T3-L1 adipogenesis, compounds **1** and **3** were selected and used to investigate the binding mode to the PPAR*γ* protein using the molecular docking method. As shown in Fig. 7A, D, **1** and **3** were docked to the same pocket of the PPAR*γ* protein. It was found that four hydrogen bonds were formed between **1** and residues of Glu291, Ser342, His466, and Ile341 (Fig. 7B) and five hydrophobic interactions between **1** and residues of Thr268, Pro269, Leu270, Arg288, and Ile341 in the binding pocket (Fig. 7C). However, three hydrogen bonds and eight hydrophobic interactions were found between **3** and PPAR*γ* protein (Fig. 7E, F). The above analysis confirmed the interaction between **1** and PPAR*γ* was stronger than that between **3** and PPAR*γ*, which was consistent with the experimental results.

The cause of obesity resides in the disequilibrium between caloric intake and expenditure, culminating in the sequestration of surplus energy as lipid deposits within adipocytes. There is an imperative need to explore novel therapeutic targets that directly modulate adipocytic activity. An array of metabolic biomarkers, comprising PRDM16, IL-27, TMBIM1, NF-*κ*B, and BMAL1, exhibit potential as intervention points for obesity therapies (Wang et al. 2021; Zhao et al. 2021, 2023). For example, it has been found a robust inverse correlation between androgen receptor (AR) expression and PRDM16 levels, implicating androgens in the suppressive control of PRDM16. This insight paved the way for promising pharmacological interventions targeting PRDM16 in obesity and metabolic disorder management (Zhao et al. 2023). It was reported that IL-27 emerged as a direct modulator of adipocytes, stimulating thermogenic processes, and facilitating weight reduction through enhanced lipid and energy catabolism. Its demonstrated efficacy in ameliorating type 2 diabetes highlighted IL-27 as a novel strategic target for addressing obesity and its concomitant metabolic disruptions (Wang et al. 2021). Further, TMBIM1 has been validated as a new inhibitor of adipogenesis, playing a pivotal negative regulatory part in adipose tissue accretion and obesity-linked metabolic anomalies. This characterized TMBIM1 as a compelling molecular candidate for therapeutic intervention in obesity-related metabolic dysfunctions (Zhao et al. 2021). Collectively, these findings carried substantial import, advancing our understanding of obesity pathophysiology, and guiding the discovery of efficacious therapeutic targets.

The hallmark of obesity lies in the volumetric expansion of adipose tissue, a complex assembly comprising mature adipocytes, preadipocytes, immune cells, vascular endothelia, and extracellular matrix constituents. Mature adipocytes, distinguished by their voluminous lipid repositories in triglyceride form, encapsulated within a small nuclear framework, function primarily as energy depots. While, preadipocytes, which derive from mesodermal lineage, bear a slender fusiform morphology, centrally nucleated, and lack intraluminal lipid vacuoles. Endowed with pluripotent differentiation capabilities, they can be stimulated under defined conditions to activate adipogenic gene cascades, transitioning into mature adipocytes (Yagi et al. 2004). The pathogenesis of obesity ensues from a positive energy balance, where caloric intake surpasses expenditure, fostering lipid accretion in mature adipocytes and concurrently spurring excessive preadipocyte proliferation and differentiation, thereby amplifying adipose tissue mass and obesity development (Oussaada et al. 2019). As preadipocytes represent a renewable source for mature adipocytes, modulating their proliferative and differentiative capacities emerges as a pivotal strategy in obesity's interception and mitigation (Oussaada et al. 2019; Yagi et al. 2004). A multitude of factors influence preadipocyte proliferation and differentiation, with pivotal transcriptional regulators—PPAR*γ*, C/EBP*α*, and C/EBP*β*—central to adipogenesis orchestration (Zuo et al. 2006). Initiation of preadipocyte differentiation is marked by a sequential elevation in C/EBP*β* expression, which in turn triggers C/EBP*α* and PPAR*γ* transcription. This hierarchical activation cascade not only halts cellular mitosis but also augments lipid biosynthesis, orchestrating the transformation of preadipocytes into mature adipocytes (Hemmrich et al. 2005).

Dimeric natural compounds, which are a class of molecules with diverse chemical structures and significant pharmacological activities, are widely found in natural organisms such as fungi, bacteria, and plants (Hong et al. 2022). Due to the possibility of simultaneously binding two distinct binding sites of a target protein, a number of dimeric natural compounds exhibited stronger pharmacological activities than their monomers (Lv et al. 2023). Among them, dimeric alkaloid natural products, which were usually formed through two monomers via C–C, C–O, C–N, or N–N bonds, have become a hot topic as clinical agents or have inspired the design of structural mimics (Lombe et al. 2019; Meng et al. 2021). For these dimeric alkaloids, most of the coupling positions between the two monomers are distributed in the aromatic ring (biaryl coupling) (Bracher et al. 2004; Meng et al. 2021). However, some dimeric alkaloids of non-biaryl coupling are also noteworthy, which mainly occurred in structures containing indole fragments (Beniddir et al. 2016; Meng et al. 2021).

Indole-diterpenoids, which represent a class of metabolites with a core structure consisting of a cyclic diterpene fused with an indole moiety, have been demonstrated to be an important source of structurally new and biologically active indole-alkaloids. These indole-diterpenoids have usually been obtained as monomers from various fungi (Meng et al. 2021). In our study, indole-diterpenoids (**3** and **4**) represent a class of fungal alkaloids, which were typically obtained as monomers from various fungi. Compounds **1** and **2** represent the first example of dimeric indole-diterpenoids with a rare C-20–C-22′ linkage. Of interest lies the observation that the monomeric indole-diterpenoids often bear cytotoxic properties (Table 1); however, the dimeric derivatives isolated in our study exhibited a remarkable absence of such toxicity. This finding underscored a pivotal shift: the dimerization of these compounds served not only to mitigate their toxic potential but also to illuminate a path towards their pharmaceutical exploitation in the realm of non-oncological ailments. Furthermore, to confirm that dimeric alkaloids of **1** and **2** were a natural source, compounds **3** and **4** were mixed and stirred for three weeks in a variety of organic solvents (0.5 mg of **3** and **4** in 1.0 mL of solvents), including PE, EtOAc, CH_2_Cl_2_, and MeOH, which have been used in the separation of **1**–**4**. Upon analysis, neither **1** nor **2** was detected by HPLC in the reaction mixture, which greatly ruled out the possibility that **1** and **2** were artifacts formed spontaneously in the fermentation.

**Table 1:** Monomeric indole-diterpenoids with cytotoxicity

Compd	Source	Cytotoxicity	References
Asperindole A	*Aspergillus* sp. KMM 4676	22Rv1 cells IC_50_ = 4.9 μmol/L	Ivanets et al. (2018)
Shearilicine	*Penicillium* sp. ZO-R1-1	L5178Y cells IC_50_ = 3.6 μmol/L A2780 cell IC_50_ = 8.7 μmol/L	Ariantari et al. (2019)
Ascandinine D	*Aspergillus candidus* HDN15-152	HL-60 cells IC_50_ = 7.8 μmol/L	Zhou et al. (2021)
Epipaxilline	*Penicillium* sp. KFD28	BeL-7402 cells IC_50_ = 5.3 μmol/L	Dai et al. (2021)
Anthcolorin B	*Aspergillus versicolor* OUPS-N136	P388 cells IC_50_ = 8.5 μmol/L	Nakanishi et al. (2013)
Anthcolorin C	*Aspergillus versicolor* OUPS-N136	P388 cells IC_50_ = 2.2 μmol/L	Nakanishi et al. (2013)
Anthcolorin D	*Aspergillus versicolor* OUPS-N136	P388 cells IC_50_ = 5.5 μmol/L	Nakanishi et al. (2013)
Penicilindole A	*Eupenicillium* sp. HJ002	A549 cells IC_50_ = 5.5 μmol/L HepG2 cells IC_50_ = 1.5 μmol/L	Zheng et al. (2018)

In summary, the first examples of dimeric indole-diterpenoids (**1** and **2**) and their monomers (**3** and **4**) were obtained from the marine-derived fungus *Penicillium* sp. Compounds **1** and **2** displayed significant inhibitory effects on the differentiation of 3T3-L1 adipocyte. The novel structures and remarkable biological activities of **1** and **2** may encourage further pharmaceutical research on new drug discovery.

## Supplementary Information

Below is the link to the electronic supplementary material.

Supporting Information COSY and Key HMBC correlations and the possible configurations (2a and 2b) of 2; COSY, Key HMBC and Key NOESY correlations of 3; full spectroscopic data and quantum mechanical calculation data of 1‒3; 1H NMR, 13C NMR and MS spectra of 4; the ΔG of the M and P conformations of (3S,4R,7S,9R,13S,16S,3'S,4'R,9'R,13'S,16'S,22'R,23'R)-1 were included. The Supporting Information is available free of charge at DOI:

## Data Availability

The data that support the findings of this study are included in this published article (and its supplementary information files).
