# Supplementary material for: The first dimeric indole-diterpenoids from a marine-derived Penicillium sp. fungus and their potential for anti-obesity drugs
Source: Mar Life Sci Technol. 2024 Oct 8;7(1):120–31. doi: 10.1007/s42995-024-00253-x (PMC11871200; doi:10.1007/s42995-024-00253-x)
Supplement: Supplementary file 1 — Supporting Information COSY and Key HMBC correlations and the possible configurations (2a and 2b) of 2; COSY, Key HMBC and Key NOESY correlations of 3; full spectroscopic data and quantum mechanical calculation data of 1‒3; 1H NMR, 13C NMR and MS spectra of 4; the ΔG of the M and P conformations of (3S,4R,7S,9R,13S,16S,3'S,4'R,9'R,13'S,16'S,22'R,23'R)-1 were included. The Supporting Information is available free of charge at DOI: [file 42995_2024_253_MOESM1_ESM.docx]

**Supporting Information**

**The First** **Dimeric Indole-Diterpenoids from a Marine-Derived *Penicillium* sp. Fungus** **and Their Potential for Anti-Obesity Drugs**

Hui-Fang Du,^1^ Lei Li,^1^ Ya-Hui Zhang,^1^ Xu Wang,^1^ Cheng-Yan Zhou,^1^ Hua-Jie Zhu,^3^ Charles U. Pittman JR,^4^ Jia-Wen Shou,^2,^* and Fei Cao,^1,2,^*

^1^College of Pharmaceutical Sciences, Key Laboratory of Medicinal Chemistry and Molecular Diagnostics of Education Ministry of China, Key Laboratory of Pharmaceutical Quality Control of Hebei Province, Hebei University, Baoding 071002, China.

^2^School of Life Sciences, The Chinese University of Hong Kong, Hong Kong, China.

^3^School of Chemistry and Pharmaceutical Engineering, Hebei University of Science and Technology, Shijiazhuang 050018, China.

^4^Department of Chemistry, Mississippi State University, Mississippi, 39762, MS, USA.

**Corresponding Authors**

*E-mail: shoujiawen@163.com (J-.W. Shou); caofei542927001@163.com (F. Cao).

**List of Supporting Information**

**Figure S1.** COSY and Key HMBC correlations of **2**‧‧‧‧‧‧‧‧‧‧‧‧‧‧‧‧‧‧‧‧‧‧‧‧‧‧‧‧‧‧‧‧‧‧‧‧‧‧‧‧‧‧‧‧‧‧‧‧‧‧‧‧‧‧‧‧‧‧‧‧‧‧‧‧‧‧‧‧‧‧‧‧S5

**Figure S2.** The possible configurations (**2a** and **2b**) of **2**‧‧‧‧‧‧‧‧‧‧‧‧‧‧‧‧‧‧‧‧‧‧‧‧‧‧‧‧‧‧‧‧‧‧‧‧‧‧‧‧‧‧‧‧‧‧‧‧‧‧‧‧‧‧‧‧‧‧‧‧‧‧‧‧‧‧S5

**Figure S3.** COSY and Key HMBC correlations of **3**‧‧‧‧‧‧‧‧‧‧‧‧‧‧‧‧‧‧‧‧‧‧‧‧‧‧‧‧‧‧‧‧‧‧‧‧‧‧‧‧‧‧‧‧‧‧‧‧‧‧‧‧‧‧‧‧‧‧‧‧‧‧‧‧‧‧‧‧‧‧‧‧S5

**Figure S4.** Key NOESY correlations of **3**‧‧‧‧‧‧‧‧‧‧‧‧‧‧‧‧‧‧‧‧‧‧‧‧‧‧‧‧‧‧‧‧‧‧‧‧‧‧‧‧‧‧‧‧‧‧‧‧‧‧‧‧‧‧‧‧‧‧‧‧‧‧‧‧‧‧‧‧‧‧‧‧‧‧‧‧‧‧‧‧‧‧‧‧‧‧‧‧‧S5

**Figure S5.** ^1^H NMR (600 MHz, CDCl_3_) spectrum of compound **1**‧‧‧‧‧‧‧‧‧‧‧‧‧‧‧‧‧‧‧‧‧‧‧‧‧‧‧‧‧‧‧‧‧‧‧‧‧‧‧‧‧‧‧‧‧‧‧‧‧‧‧S6

**Figure S6.** Partial ^1^H NMR (600 MHz, CDCl_3_) spectrum of compound **1**‧‧‧‧‧‧‧‧‧‧‧‧‧‧‧‧‧‧‧‧‧‧‧‧‧‧‧‧‧‧‧‧‧‧‧‧‧‧‧‧S6

**Figure S7.** Partial ^1^H NMR (600 MHz, CDCl_3_) spectrum of compound **1**‧‧‧‧‧‧‧‧‧‧‧‧‧‧‧‧‧‧‧‧‧‧‧‧‧‧‧‧‧‧‧‧‧‧‧‧‧‧‧‧S7

**Figure S8.** Partial ^1^H NMR (600 MHz, CDCl_3_) spectrum of compound **1**‧‧‧‧‧‧‧‧‧‧‧‧‧‧‧‧‧‧‧‧‧‧‧‧‧‧‧‧‧‧‧‧‧‧‧‧‧‧‧‧S7

**Figure S9.** ^13^C NMR (150 MHz, CDCl_3_) spectrum of compound **1**‧‧‧‧‧‧‧‧‧‧‧‧‧‧‧‧‧‧‧‧‧‧‧‧‧‧‧‧‧‧‧‧‧‧‧‧‧‧‧‧‧‧‧‧‧‧‧‧‧‧S8

**Figure S10.** Partial ^13^C NMR (150 MHz, CDCl_3_) spectrum of compound **1**‧‧‧‧‧‧‧‧‧‧‧‧‧‧‧‧‧‧‧‧‧‧‧‧‧‧‧‧‧‧‧‧‧‧‧‧‧S8

**Figure S11.** Partial ^13^C NMR (150 MHz, CDCl_3_) spectrum of compound **1**‧‧‧‧‧‧‧‧‧‧‧‧‧‧‧‧‧‧‧‧‧‧‧‧‧‧‧‧‧‧‧‧‧‧‧‧‧S9

**Figure S12.** HSQC (CDCl_3_) spectrum of compound **1**‧‧‧‧‧‧‧‧‧‧‧‧‧‧‧‧‧‧‧‧‧‧‧‧‧‧‧‧‧‧‧‧‧‧‧‧‧‧‧‧‧‧‧‧‧‧‧‧‧‧‧‧‧‧‧‧‧‧‧‧‧‧‧‧‧‧‧‧‧S9

**Figure S13.** Partial HSQC (CDCl_3_) spectrum of compound **1**‧‧‧‧‧‧‧‧‧‧‧‧‧‧‧‧‧‧‧‧‧‧‧‧‧‧‧‧‧‧‧‧‧‧‧‧‧‧‧‧‧‧‧‧‧‧‧‧‧‧‧‧‧‧‧‧S10

**Figure S14.** Partial HSQC (CDCl_3_) spectrum of compound **1**‧‧‧‧‧‧‧‧‧‧‧‧‧‧‧‧‧‧‧‧‧‧‧‧‧‧‧‧‧‧‧‧‧‧‧‧‧‧‧‧‧‧‧‧‧‧‧‧‧‧‧‧‧‧‧‧S10

**Figure S15.** ^1^H-^1^H COSY (CDCl_3_) spectrum of compound **1**‧‧‧‧‧‧‧‧‧‧‧‧‧‧‧‧‧‧‧‧‧‧‧‧‧‧‧‧‧‧‧‧‧‧‧‧‧‧‧‧‧‧‧‧‧‧‧‧‧‧‧‧‧‧‧‧‧S11

**Figure S16.** Partial ^1^H-^1^H COSY (CDCl_3_) spectrum of compound **1**‧‧‧‧‧‧‧‧‧‧‧‧‧‧‧‧‧‧‧‧‧‧‧‧‧‧‧‧‧‧‧‧‧‧‧‧‧‧‧‧‧‧‧‧‧‧S11

**Figure S17.** HMBC (CDCl_3_) spectrum of compound **1**‧‧‧‧‧‧‧‧‧‧‧‧‧‧‧‧‧‧‧‧‧‧‧‧‧‧‧‧‧‧‧‧‧‧‧‧‧‧‧‧‧‧‧‧‧‧‧‧‧‧‧‧‧‧‧‧‧‧‧‧‧‧‧‧‧‧S12

**Figure S18.** Partial HMBC (CDCl_3_) spectrum of compound **1**‧‧‧‧‧‧‧‧‧‧‧‧‧‧‧‧‧‧‧‧‧‧‧‧‧‧‧‧‧‧‧‧‧‧‧‧‧‧‧‧‧‧‧‧‧‧‧‧‧‧‧‧‧‧‧S12

**Figure S19.** Partial HMBC (CDCl_3_) spectrum of compound **1**‧‧‧‧‧‧‧‧‧‧‧‧‧‧‧‧‧‧‧‧‧‧‧‧‧‧‧‧‧‧‧‧‧‧‧‧‧‧‧‧‧‧‧‧‧‧‧‧‧‧‧‧‧‧‧S13

**Figure S20.** Partial HMBC (CDCl_3_) spectrum of compound **1**‧‧‧‧‧‧‧‧‧‧‧‧‧‧‧‧‧‧‧‧‧‧‧‧‧‧‧‧‧‧‧‧‧‧‧‧‧‧‧‧‧‧‧‧‧‧‧‧‧‧‧‧‧‧‧S13

**Figure S21.** NOESY (CDCl_3_) spectrum of compound **1**‧‧‧‧‧‧‧‧‧‧‧‧‧‧‧‧‧‧‧‧‧‧‧‧‧‧‧‧‧‧‧‧‧‧‧‧‧‧‧‧‧‧‧‧‧‧‧‧‧‧‧‧‧‧‧‧‧‧‧‧‧‧‧‧S14

**Figure S22.** Partial NOESY (CDCl_3_) spectrum of compound **1**‧‧‧‧‧‧‧‧‧‧‧‧‧‧‧‧‧‧‧‧‧‧‧‧‧‧‧‧‧‧‧‧‧‧‧‧‧‧‧‧‧‧‧‧‧‧‧‧‧‧‧‧‧S14

**Figure S23.** HRESIMS spectrum of compound **1**‧‧‧‧‧‧‧‧‧‧‧‧‧‧‧‧‧‧‧‧‧‧‧‧‧‧‧‧‧‧‧‧‧‧‧‧‧‧‧‧‧‧‧‧‧‧‧‧‧‧‧‧‧‧‧‧‧‧‧‧‧‧‧‧‧‧‧‧‧‧‧‧‧‧S15

**Figure S24.** ^1^H NMR (600 MHz, CDCl_3_) spectrum of compound **2**‧‧‧‧‧‧‧‧‧‧‧‧‧‧‧‧‧‧‧‧‧‧‧‧‧‧‧‧‧‧‧‧‧‧‧‧‧‧‧‧‧‧‧‧‧‧‧S15

**Figure S25.** Partial ^1^H NMR (600 MHz, CDCl_3_) spectrum of compound **2**‧‧‧‧‧‧‧‧‧‧‧‧‧‧‧‧‧‧‧‧‧‧‧‧‧‧‧‧‧‧‧‧‧‧‧‧S16

**Figure S26.** Partial ^1^H NMR (600 MHz, CDCl_3_) spectrum of compound **2**‧‧‧‧‧‧‧‧‧‧‧‧‧‧‧‧‧‧‧‧‧‧‧‧‧‧‧‧‧‧‧‧‧‧‧‧S16

**Figure S27.** Partial ^1^H NMR (600 MHz, CDCl_3_) spectrum of compound **2**‧‧‧‧‧‧‧‧‧‧‧‧‧‧‧‧‧‧‧‧‧‧‧‧‧‧‧‧‧‧‧‧‧‧‧‧S17

**Figure S28.** ^13^C NMR (150 MHz, CDCl_3_) spectrum of compound **2**‧‧‧‧‧‧‧‧‧‧‧‧‧‧‧‧‧‧‧‧‧‧‧‧‧‧‧‧‧‧‧‧‧‧‧‧‧‧‧‧‧‧‧‧‧‧S17

**Figure S29.** Partial ^13^C NMR (150 MHz, CDCl_3_) spectrum of compound **2**‧‧‧‧‧‧‧‧‧‧‧‧‧‧‧‧‧‧‧‧‧‧‧‧‧‧‧‧‧‧‧‧‧‧‧S18

**Figure S30.** Partial ^13^C NMR (150 MHz, CDCl_3_) spectrum of compound **2**‧‧‧‧‧‧‧‧‧‧‧‧‧‧‧‧‧‧‧‧‧‧‧‧‧‧‧‧‧‧‧‧‧‧‧S18

**Figure S31.** HSQC (CDCl_3_) spectrum of compound **2**‧‧‧‧‧‧‧‧‧‧‧‧‧‧‧‧‧‧‧‧‧‧‧‧‧‧‧‧‧‧‧‧‧‧‧‧‧‧‧‧‧‧‧‧‧‧‧‧‧‧‧‧‧‧‧‧‧‧‧‧‧‧‧‧‧‧‧S19

**Figure S32.** Partial HSQC (CDCl_3_) spectrum of compound **2**‧‧‧‧‧‧‧‧‧‧‧‧‧‧‧‧‧‧‧‧‧‧‧‧‧‧‧‧‧‧‧‧‧‧‧‧‧‧‧‧‧‧‧‧‧‧‧‧‧‧‧‧‧‧‧‧S19

**Figure S33.** Partial HSQC (CDCl_3_) spectrum of compound **2**‧‧‧‧‧‧‧‧‧‧‧‧‧‧‧‧‧‧‧‧‧‧‧‧‧‧‧‧‧‧‧‧‧‧‧‧‧‧‧‧‧‧‧‧‧‧‧‧‧‧‧‧‧‧‧‧S20

**Figure S34.** ^1^H-^1^H COSY (CDCl_3_) spectrum of compound **2**‧‧‧‧‧‧‧‧‧‧‧‧‧‧‧‧‧‧‧‧‧‧‧‧‧‧‧‧‧‧‧‧‧‧‧‧‧‧‧‧‧‧‧‧‧‧‧‧‧‧‧‧‧‧‧‧‧S20

**Figure S35.** Partial ^1^H-^1^H COSY (CDCl_3_) spectrum of compound **2**‧‧‧‧‧‧‧‧‧‧‧‧‧‧‧‧‧‧‧‧‧‧‧‧‧‧‧‧‧‧‧‧‧‧‧‧‧‧‧‧‧‧‧‧‧‧S21

**Figure S36.** HMBC (CDCl_3_) spectrum of compound **2**‧‧‧‧‧‧‧‧‧‧‧‧‧‧‧‧‧‧‧‧‧‧‧‧‧‧‧‧‧‧‧‧‧‧‧‧‧‧‧‧‧‧‧‧‧‧‧‧‧‧‧‧‧‧‧‧‧‧‧‧‧‧‧‧‧‧S21

**Figure S37.** Partial HMBC (CDCl_3_) spectrum of compound **2**‧‧‧‧‧‧‧‧‧‧‧‧‧‧‧‧‧‧‧‧‧‧‧‧‧‧‧‧‧‧‧‧‧‧‧‧‧‧‧‧‧‧‧‧‧‧‧‧‧‧‧‧‧‧‧S22

**Figure S38.** Partial HMBC (CDCl_3_) spectrum of compound **2**‧‧‧‧‧‧‧‧‧‧‧‧‧‧‧‧‧‧‧‧‧‧‧‧‧‧‧‧‧‧‧‧‧‧‧‧‧‧‧‧‧‧‧‧‧‧‧‧‧‧‧‧‧‧‧S22

**Figure S39.** Partial HMBC (CDCl_3_) spectrum of compound **2**‧‧‧‧‧‧‧‧‧‧‧‧‧‧‧‧‧‧‧‧‧‧‧‧‧‧‧‧‧‧‧‧‧‧‧‧‧‧‧‧‧‧‧‧‧‧‧‧‧‧‧‧‧‧‧S23

**Figure S40.** NOESY (CDCl_3_) spectrum of compound **2**‧‧‧‧‧‧‧‧‧‧‧‧‧‧‧‧‧‧‧‧‧‧‧‧‧‧‧‧‧‧‧‧‧‧‧‧‧‧‧‧‧‧‧‧‧‧‧‧‧‧‧‧‧‧‧‧‧‧‧‧‧‧‧‧S23

**Figure S41.** Partial NOESY (CDCl_3_) spectrum of compound **2**‧‧‧‧‧‧‧‧‧‧‧‧‧‧‧‧‧‧‧‧‧‧‧‧‧‧‧‧‧‧‧‧‧‧‧‧‧‧‧‧‧‧‧‧‧‧‧‧‧‧‧‧‧‧S24

**Figure S42.** HRESIMS spectrum of compound **2**‧‧‧‧‧‧‧‧‧‧‧‧‧‧‧‧‧‧‧‧‧‧‧‧‧‧‧‧‧‧‧‧‧‧‧‧‧‧‧‧‧‧‧‧‧‧‧‧‧‧‧‧‧‧‧‧‧‧‧‧‧‧‧‧‧‧‧‧‧‧‧‧‧‧S24

**Figure S43.** ^1^H NMR (600 MHz, CDCl_3_) spectrum of compound **3**‧‧‧‧‧‧‧‧‧‧‧‧‧‧‧‧‧‧‧‧‧‧‧‧‧‧‧‧‧‧‧‧‧‧‧‧‧‧‧‧‧‧‧‧‧‧‧S25

**Figure S44.** Partial ^1^H NMR (600 MHz, CDCl_3_) spectrum of compound **3**‧‧‧‧‧‧‧‧‧‧‧‧‧‧‧‧‧‧‧‧‧‧‧‧‧‧‧‧‧‧‧‧‧‧‧‧S25

**Figure S45.** ^13^C NMR (150 MHz, CDCl_3_) spectrum of compound **3**‧‧‧‧‧‧‧‧‧‧‧‧‧‧‧‧‧‧‧‧‧‧‧‧‧‧‧‧‧‧‧‧‧‧‧‧‧‧‧‧‧‧‧‧‧‧S26

**Figure S46.** Partial ^13^C NMR (150 MHz, CDCl_3_) spectrum of compound **3**‧‧‧‧‧‧‧‧‧‧‧‧‧‧‧‧‧‧‧‧‧‧‧‧‧‧‧‧‧‧‧‧‧‧‧S26

**Figure S47.** HSQC (CDCl_3_) spectrum of compound **3**‧‧‧‧‧‧‧‧‧‧‧‧‧‧‧‧‧‧‧‧‧‧‧‧‧‧‧‧‧‧‧‧‧‧‧‧‧‧‧‧‧‧‧‧‧‧‧‧‧‧‧‧‧‧‧‧‧‧‧‧‧‧‧‧‧‧‧S27

**Figure S48.** Partial HSQC (CDCl_3_) spectrum of compound **3**‧‧‧‧‧‧‧‧‧‧‧‧‧‧‧‧‧‧‧‧‧‧‧‧‧‧‧‧‧‧‧‧‧‧‧‧‧‧‧‧‧‧‧‧‧‧‧‧‧‧‧‧‧‧‧‧S27

**Figure S49.** ^1^H-^1^H COSY (CDCl_3_) spectrum of compound **3**‧‧‧‧‧‧‧‧‧‧‧‧‧‧‧‧‧‧‧‧‧‧‧‧‧‧‧‧‧‧‧‧‧‧‧‧‧‧‧‧‧‧‧‧‧‧‧‧‧‧‧‧‧‧‧‧‧S28

**Figure S50.** Partial ^1^H-^1^H COSY (CDCl_3_) spectrum of compound **3**‧‧‧‧‧‧‧‧‧‧‧‧‧‧‧‧‧‧‧‧‧‧‧‧‧‧‧‧‧‧‧‧‧‧‧‧‧‧‧‧‧‧‧‧‧‧S28

**Figure S51.** HMBC (CDCl_3_) spectrum of compound **3**‧‧‧‧‧‧‧‧‧‧‧‧‧‧‧‧‧‧‧‧‧‧‧‧‧‧‧‧‧‧‧‧‧‧‧‧‧‧‧‧‧‧‧‧‧‧‧‧‧‧‧‧‧‧‧‧‧‧‧‧‧‧‧‧‧‧S29

**Figure S52.** Partial HMBC (CDCl_3_) spectrum of compound **3**‧‧‧‧‧‧‧‧‧‧‧‧‧‧‧‧‧‧‧‧‧‧‧‧‧‧‧‧‧‧‧‧‧‧‧‧‧‧‧‧‧‧‧‧‧‧‧‧‧‧‧‧‧‧‧S29

**Figure S53.** Partial HMBC (CDCl_3_) spectrum of compound **3**‧‧‧‧‧‧‧‧‧‧‧‧‧‧‧‧‧‧‧‧‧‧‧‧‧‧‧‧‧‧‧‧‧‧‧‧‧‧‧‧‧‧‧‧‧‧‧‧‧‧‧‧‧‧‧S30

**Figure S54.** NOESY (CDCl_3_) spectrum of compound **3**‧‧‧‧‧‧‧‧‧‧‧‧‧‧‧‧‧‧‧‧‧‧‧‧‧‧‧‧‧‧‧‧‧‧‧‧‧‧‧‧‧‧‧‧‧‧‧‧‧‧‧‧‧‧‧‧‧‧‧‧‧‧‧‧S30

**Figure S55.** Partial NOESY (CDCl_3_) spectrum of compound **3**‧‧‧‧‧‧‧‧‧‧‧‧‧‧‧‧‧‧‧‧‧‧‧‧‧‧‧‧‧‧‧‧‧‧‧‧‧‧‧‧‧‧‧‧‧‧‧‧‧‧‧‧‧‧S31

**Figure S56.** HRESIMS spectrum of compound **3**‧‧‧‧‧‧‧‧‧‧‧‧‧‧‧‧‧‧‧‧‧‧‧‧‧‧‧‧‧‧‧‧‧‧‧‧‧‧‧‧‧‧‧‧‧‧‧‧‧‧‧‧‧‧‧‧‧‧‧‧‧‧‧‧‧‧‧‧‧‧‧‧‧‧S31

**Figure S57.** ^1^H NMR (600 MHz, CDCl_3_) spectrum of compound **4**‧‧‧‧‧‧‧‧‧‧‧‧‧‧‧‧‧‧‧‧‧‧‧‧‧‧‧‧‧‧‧‧‧‧‧‧‧‧‧‧‧‧‧‧‧‧‧S32

**Figure S58.** ^13^C NMR (150 MHz, CDCl_3_) spectrum of compound **4**‧‧‧‧‧‧‧‧‧‧‧‧‧‧‧‧‧‧‧‧‧‧‧‧‧‧‧‧‧‧‧‧‧‧‧‧‧‧‧‧‧‧‧‧‧‧S32

**Figure S59.** HRESIMS spectrum of compound **4**‧‧‧‧‧‧‧‧‧‧‧‧‧‧‧‧‧‧‧‧‧‧‧‧‧‧‧‧‧‧‧‧‧‧‧‧‧‧‧‧‧‧‧‧‧‧‧‧‧‧‧‧‧‧‧‧‧‧‧‧‧‧‧‧‧‧‧‧‧‧‧‧‧‧S33

**Figure S60.** The ΔG of the *M* and *P* conformations of (3*S*,4*R*,7*S*,9*R*,13*S*,16*S*,3'*S*,4'*R*,9'*R*,13'*S*,16'*S*,22'*R*,23'*R*)-**1**‧‧‧‧‧‧‧‧‧‧‧‧‧‧‧‧‧‧‧‧‧‧‧‧‧‧‧‧‧‧‧‧‧‧‧‧‧‧‧‧‧‧‧‧‧‧‧‧‧‧‧‧‧‧‧‧‧‧‧‧‧‧S34

**Table S1.** ^1^H and ^13^C NMR Data of **1** (600 MHz, CDCl_3_, *J* in Hz) ‧‧‧‧‧‧‧‧‧‧‧‧‧‧‧‧‧‧‧‧‧‧‧‧‧‧‧‧‧‧‧‧‧‧‧‧‧‧‧‧‧‧‧‧‧‧‧‧‧S35

**Table S2.** ^1^H and ^13^C NMR Data of **2** (600 MHz, CDCl_3_, *J* in Hz) ‧‧‧‧‧‧‧‧‧‧‧‧‧‧‧‧‧‧‧‧‧‧‧‧‧‧‧‧‧‧‧‧‧‧‧‧‧‧‧‧‧‧‧‧‧‧‧‧‧S36

**Table S3.** ^1^H and ^13^C NMR Data of **3** (600 MHz, CDCl_3_, *J* in Hz) ‧‧‧‧‧‧‧‧‧‧‧‧‧‧‧‧‧‧‧‧‧‧‧‧‧‧‧‧‧‧‧‧‧‧‧‧‧‧‧‧‧‧‧‧‧‧‧‧‧S37

**Table S4.** The coordinates for the lowest-energy conformer of **1a** in ECD calculation‧‧‧‧‧‧‧‧‧‧‧‧‧‧‧‧‧‧‧S38

**Table S5.** The coordinates for the lowest-energy conformer of *ent*-**1a** in ECD calculation‧‧‧‧‧‧‧‧‧‧‧‧S41

**Table S6.** The coordinates for the lowest-energy conformer of **1b** in ECD calculation‧‧‧‧‧‧‧‧‧‧‧‧‧‧‧‧‧‧S44

**Table S7.** The coordinates for the lowest-energy conformer of *ent*-**1b** in ECD calculation‧‧‧‧‧‧‧‧‧‧‧‧S47

**Table S8.** The coordinates for the lowest-energy conformer of **2a** in ECD calculation‧‧‧‧‧‧‧‧‧‧‧‧‧‧‧‧‧‧‧S50

**Table S9.** The coordinates for the lowest-energy conformer of *ent*-**2a** in ECD calculation‧‧‧‧‧‧‧‧‧‧‧‧S53

**Table S10.** The coordinates for the lowest-energy conformer of **2b** in ECD calculation‧‧‧‧‧‧‧‧‧‧‧‧‧‧‧‧S56

**Table S11.** The coordinates for the lowest-energy conformer of *ent*-**2b** in ECD calculation‧‧‧‧‧‧‧‧‧‧S59

**Table S12.** The coordinates for the lowest-energy conformer of **3** in ECD calculation‧‧‧‧‧‧‧‧‧‧‧‧‧‧‧‧‧‧‧S62

**Table S13.** The coordinates for the lowest-energy conformer of *ent*-**3** in ECD calculation‧‧‧‧‧‧‧‧‧‧‧‧S64

**Figure S1.** COSY and Key HMBC correlations of **2**

**Figure S2.** The possible configurations (**2a** and **2b**) of **2**

**Figure S3.** COSY and Key HMBC correlations of **3**

**Figure S4.** Key NOESY correlations of **3**


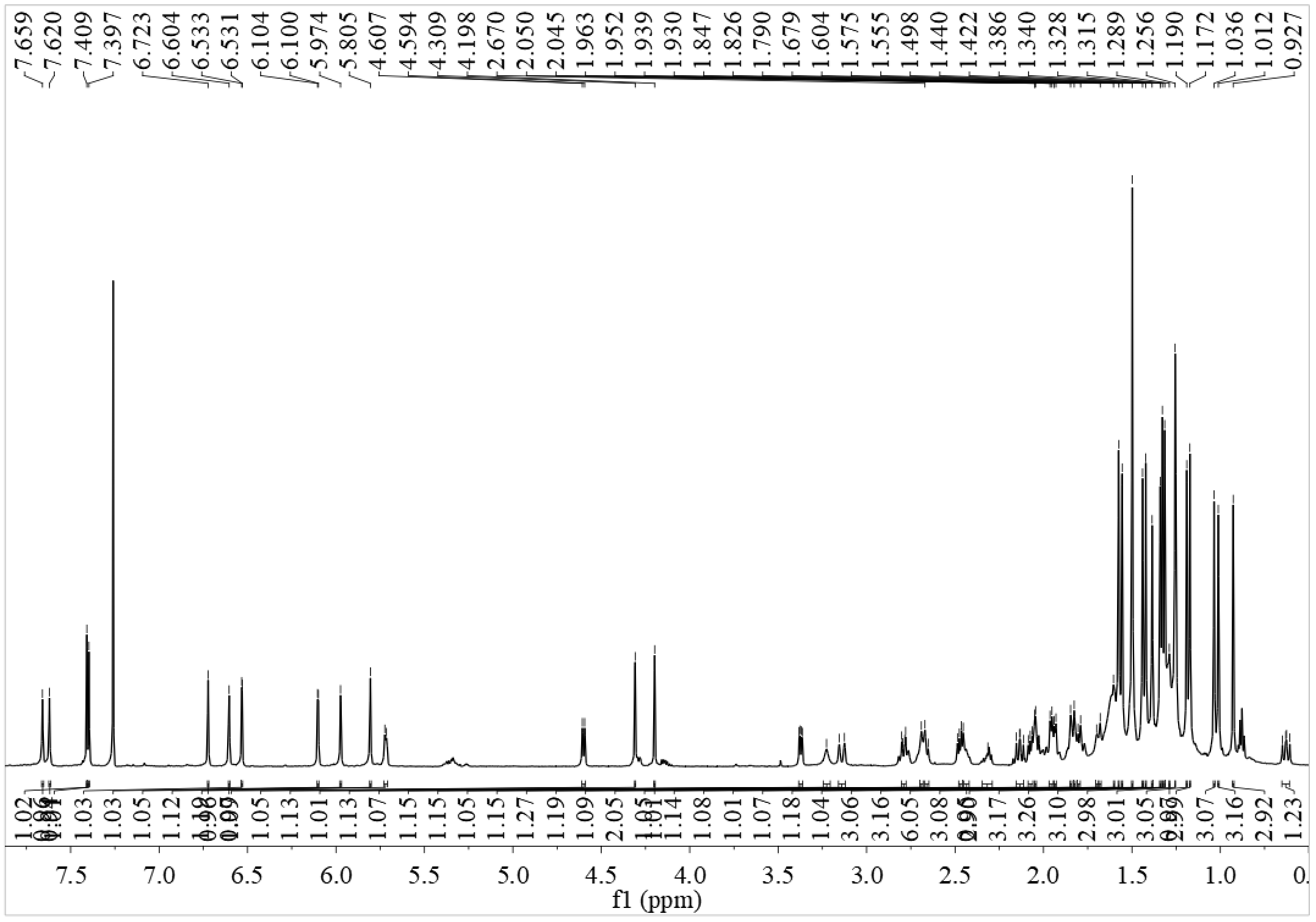


**Figure S5.** ^1^H NMR (600 MHz, CDCl_3_) spectrum of compound **1**


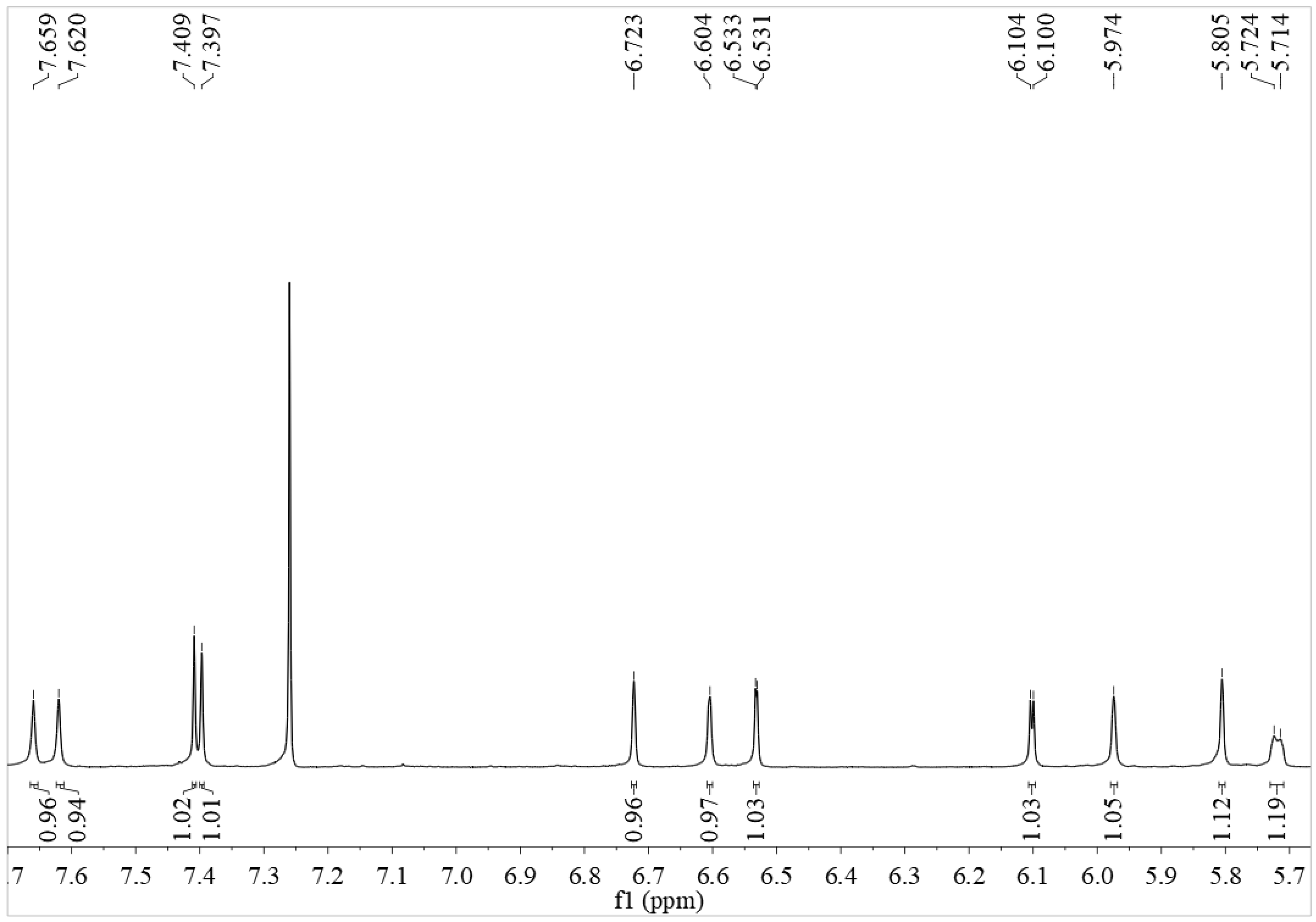


**Figure S6.** Partial ^1^H NMR (600 MHz, CDCl_3_) spectrum of compound **1**


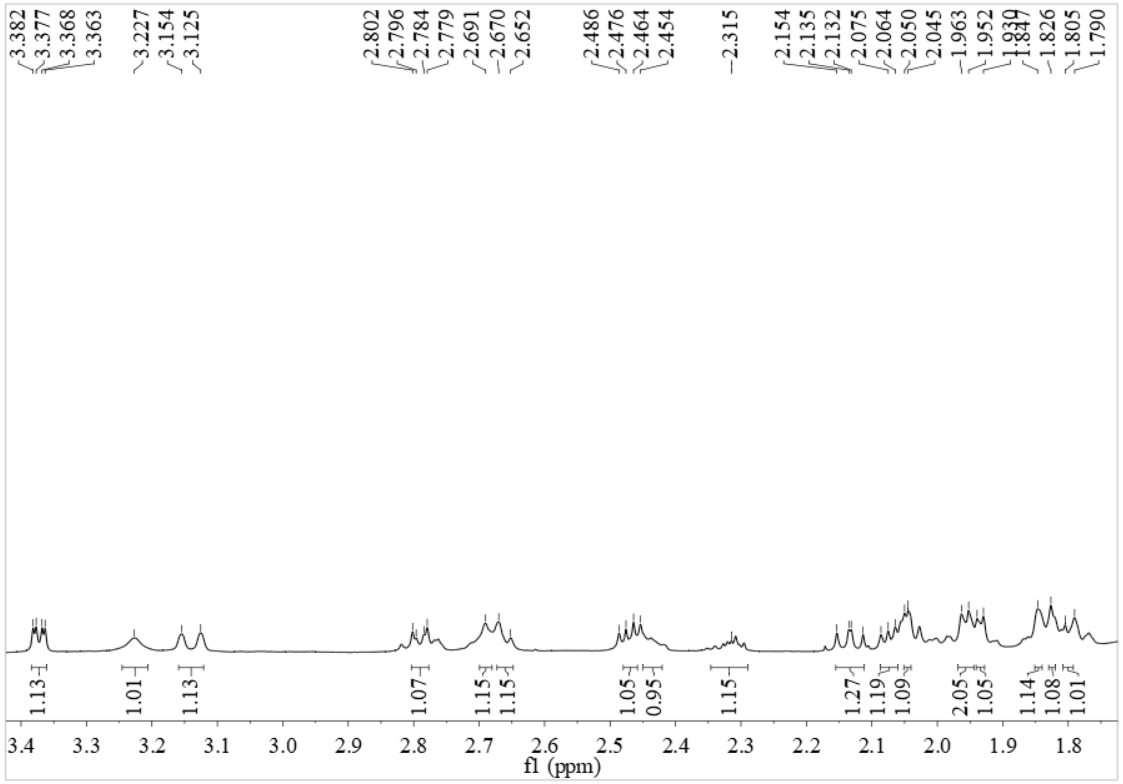


**Figure S7.** Partial ^1^H NMR (600 MHz, CDCl_3_) spectrum of compound **1**


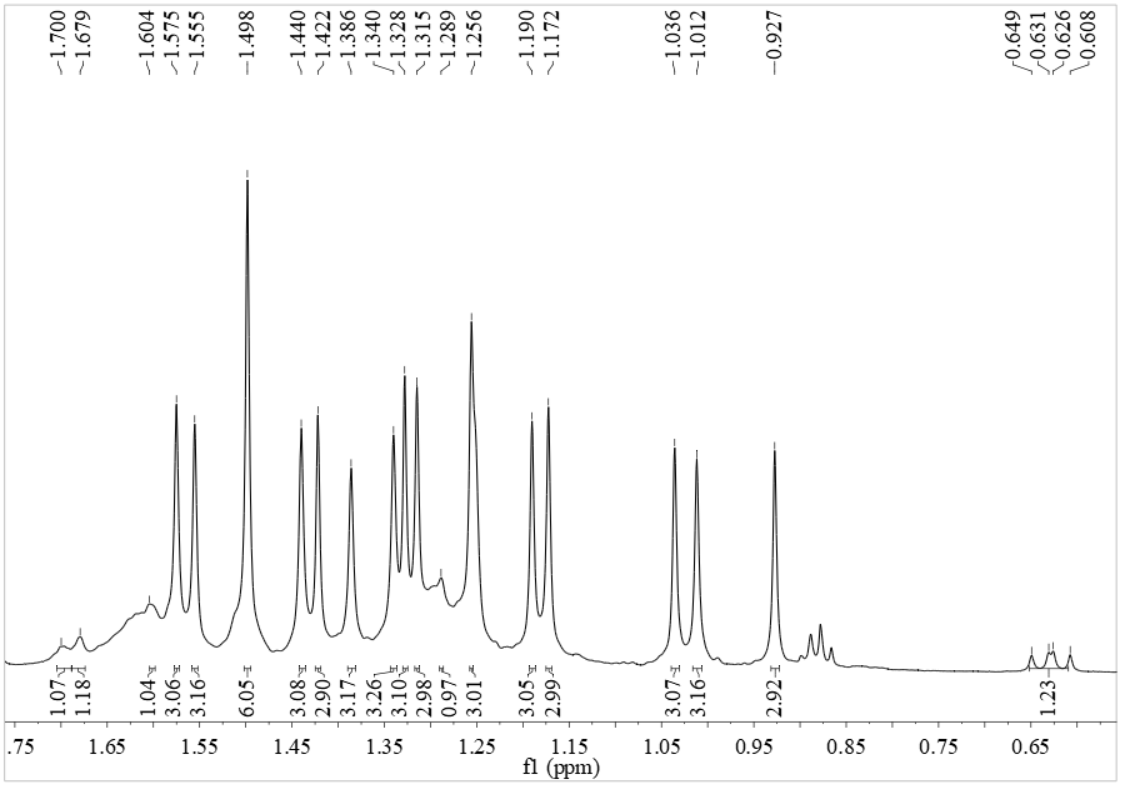


**Figure S8.** Partial ^1^H NMR (600 MHz, CDCl_3_) spectrum of compound **1**


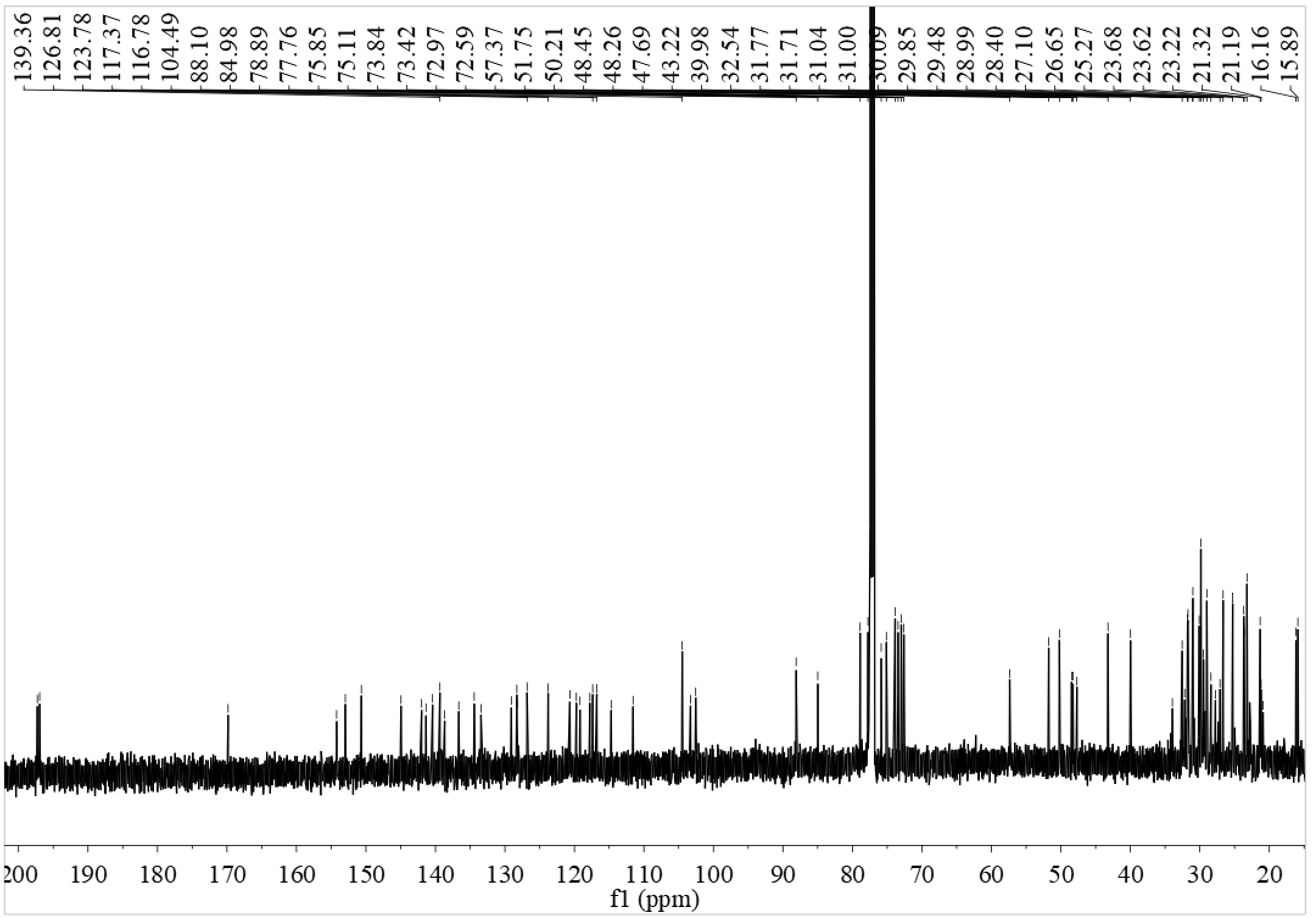


**Figure S9.** ^13^C NMR (150 MHz, CDCl_3_) spectrum of compound **1**


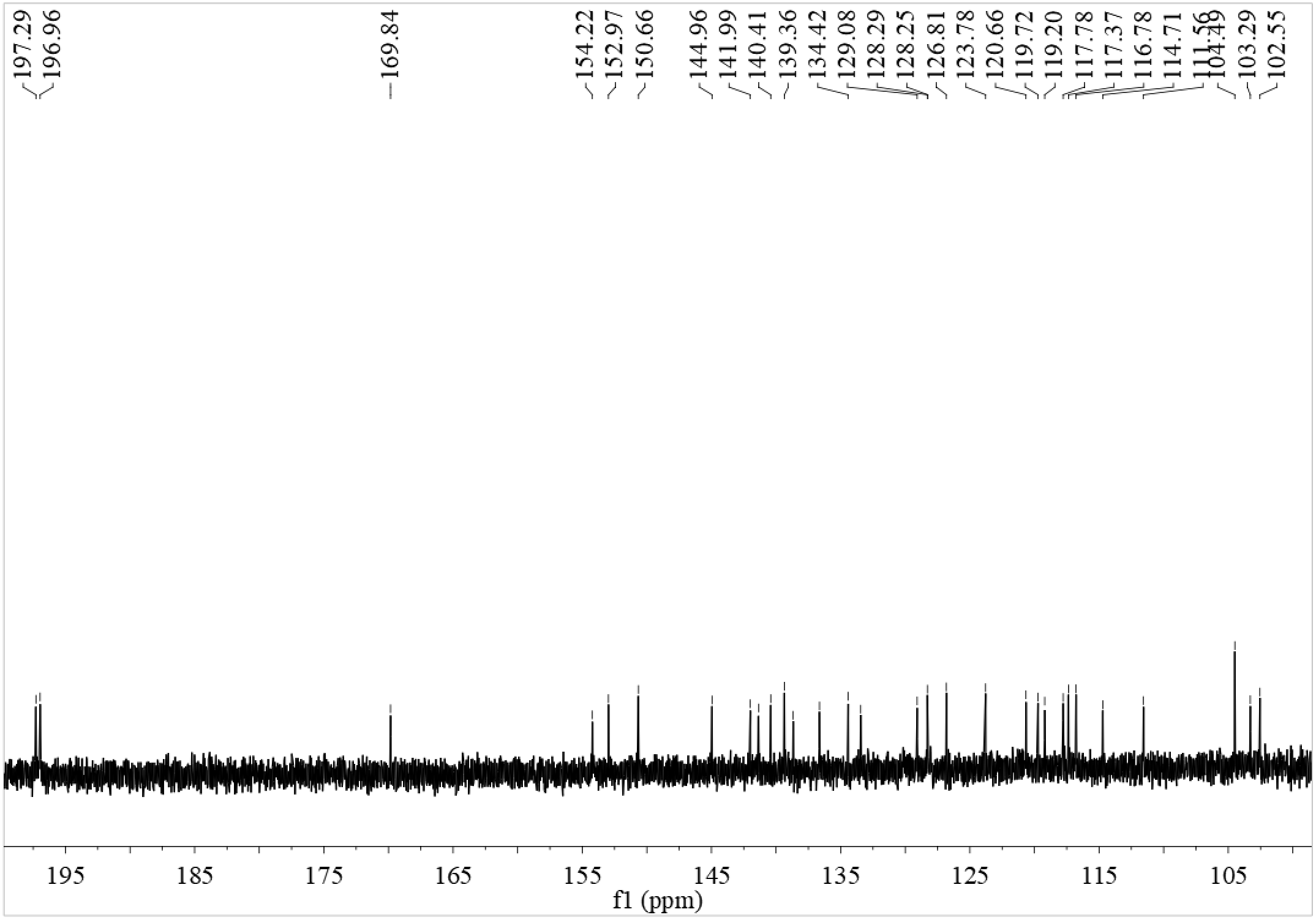


**Figure S10.** Partial ^13^C NMR (150 MHz, CDCl_3_) spectrum of compound **1**


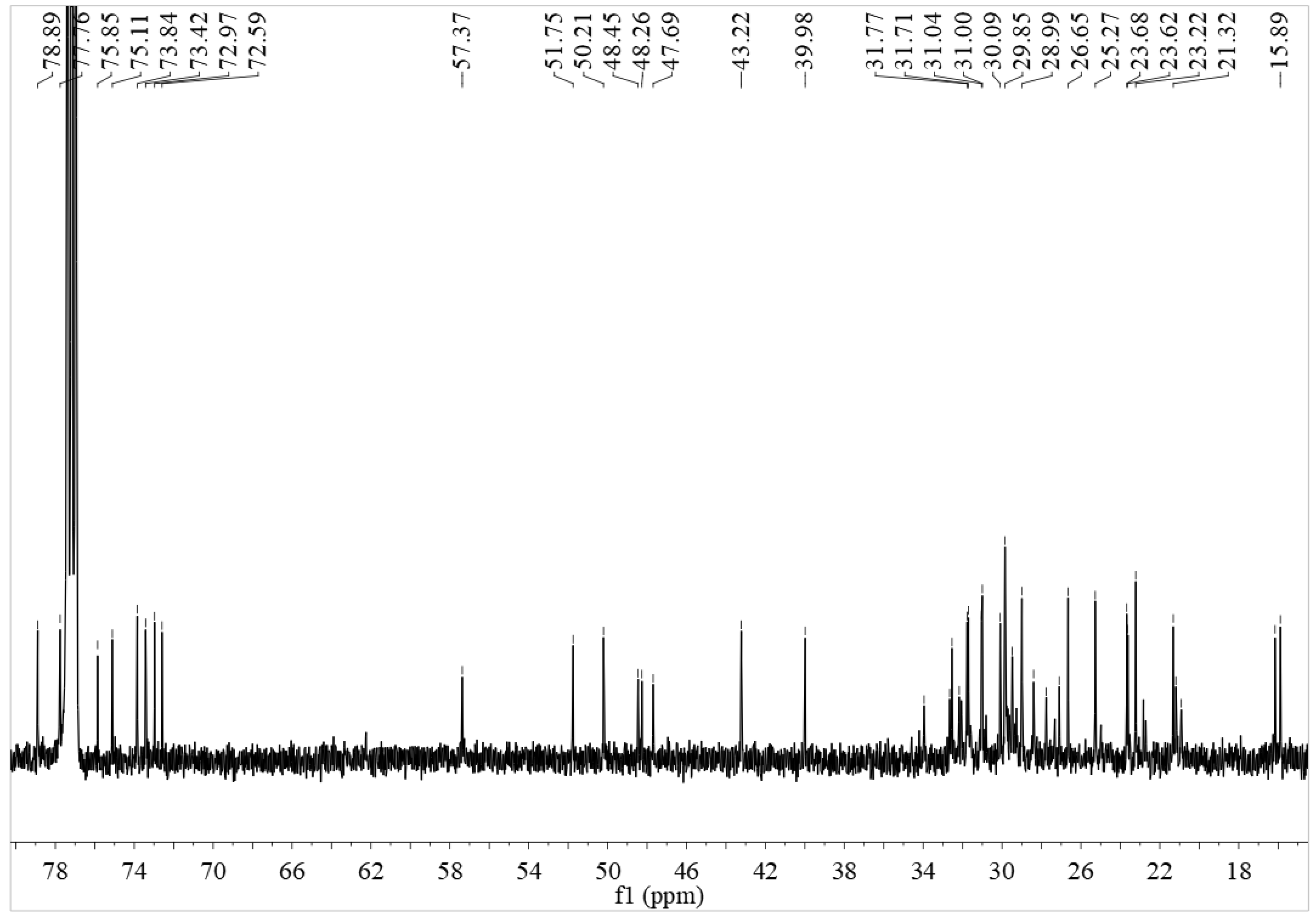


**Figure S11.** Partial ^13^C NMR (150 MHz, CDCl_3_) spectrum of compound **1**


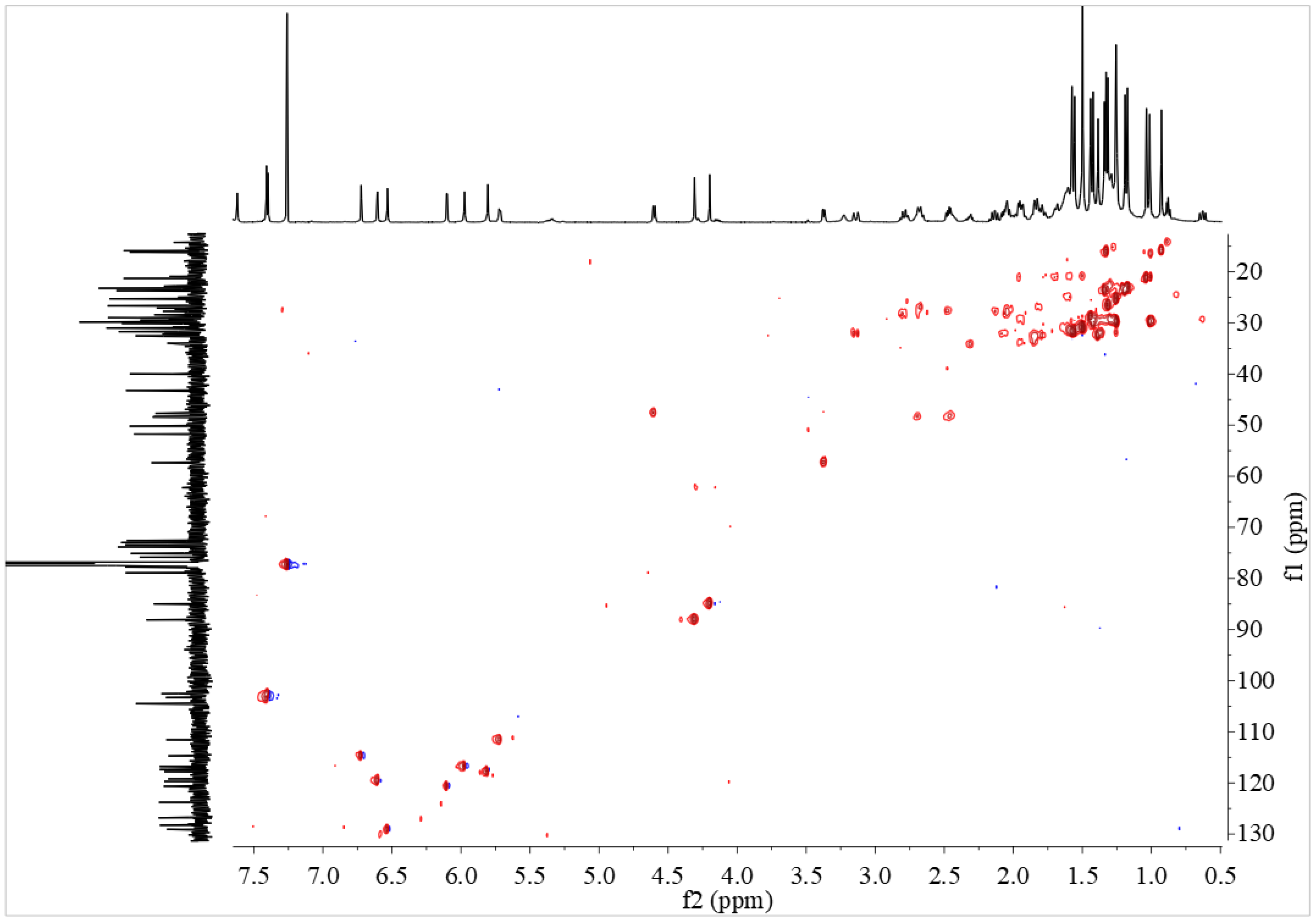


**Figure S12.** HSQC (CDCl_3_) spectrum of compound **1**


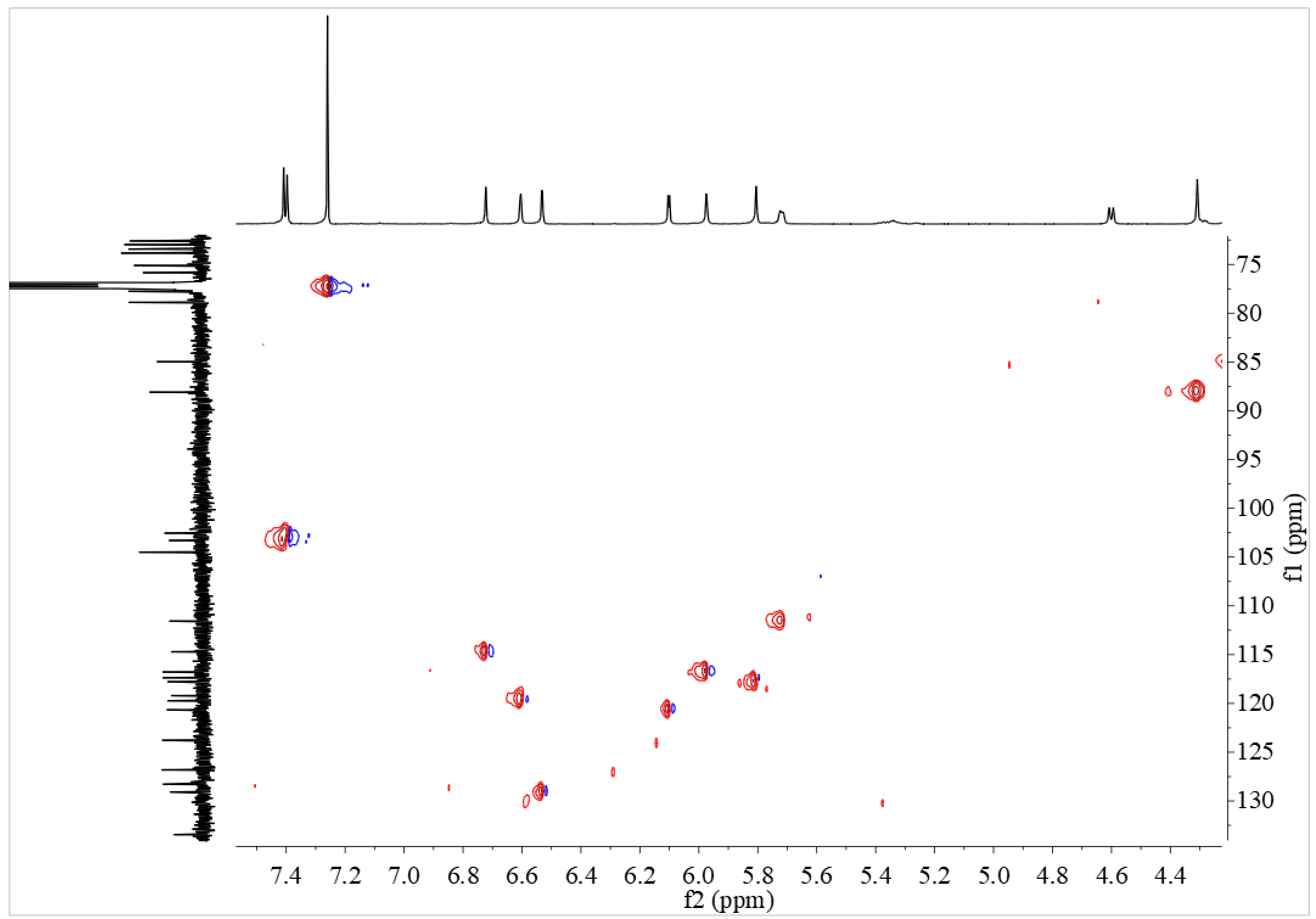


**Figure S13.** Partial HSQC (CDCl_3_) spectrum of compound **1**


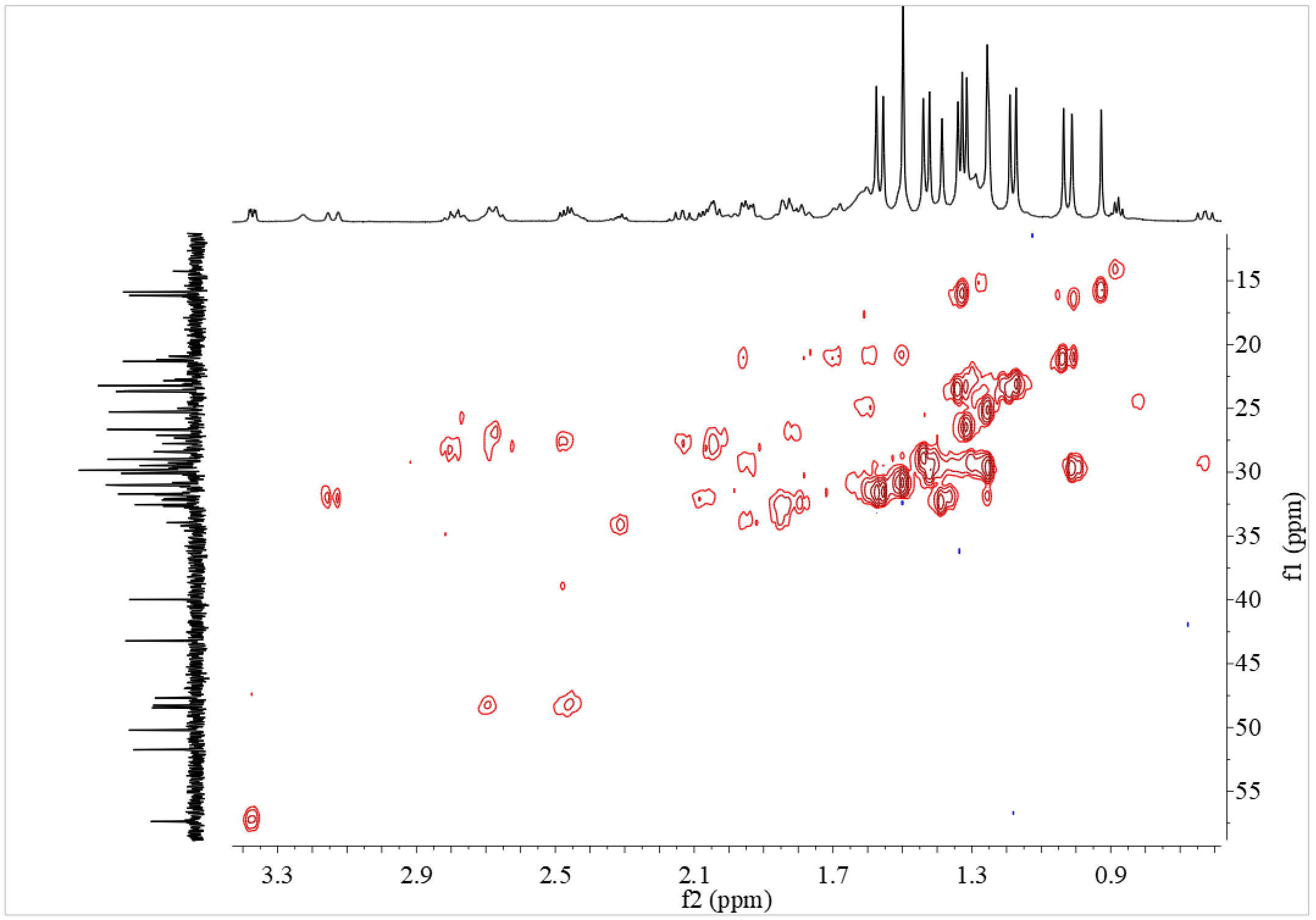


**Figure S14.** Partial HSQC (CDCl_3_) spectrum of compound **1**


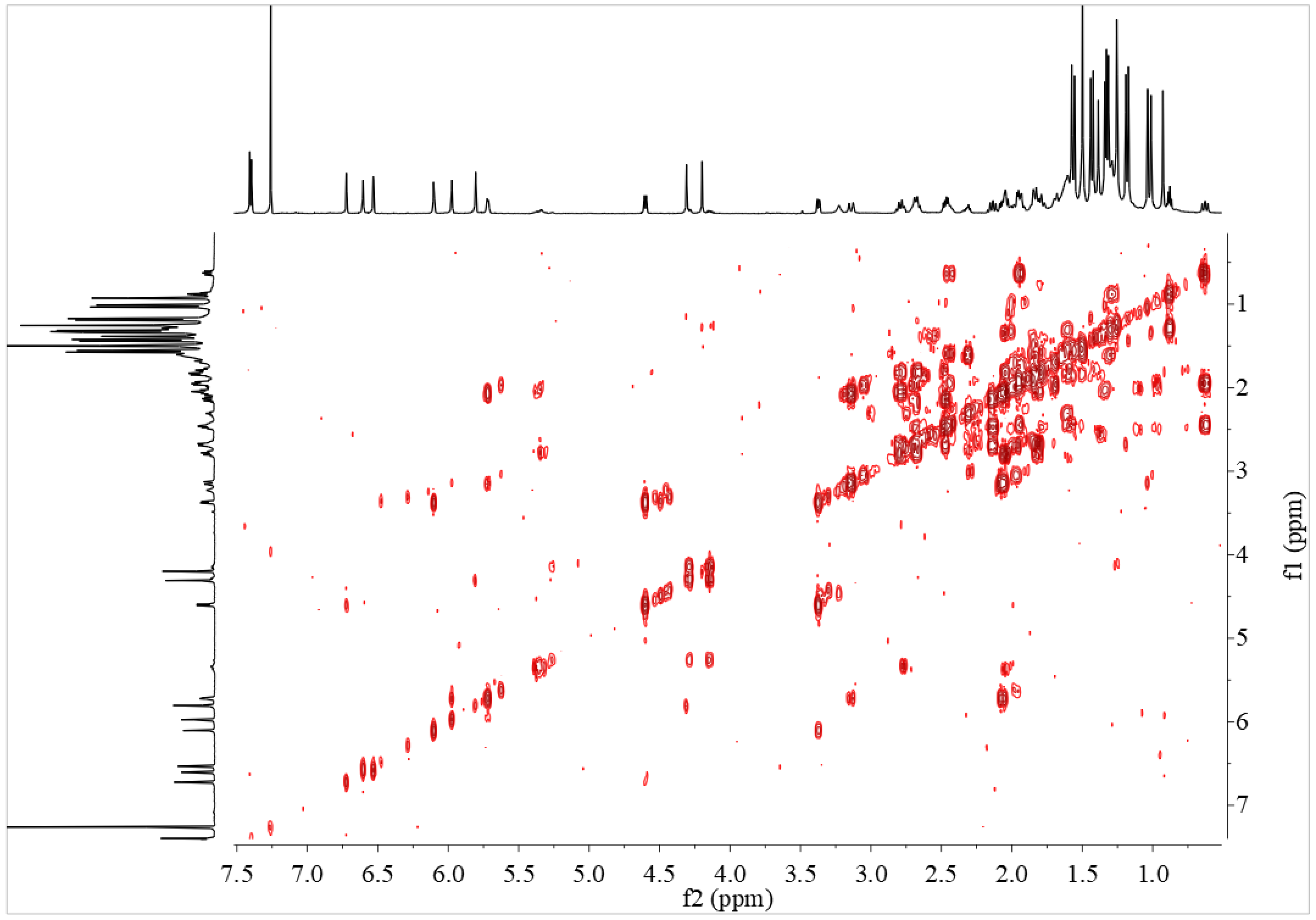


**Figure S15.** ^1^H-^1^H COSY (CDCl_3_) spectrum of compound **1**


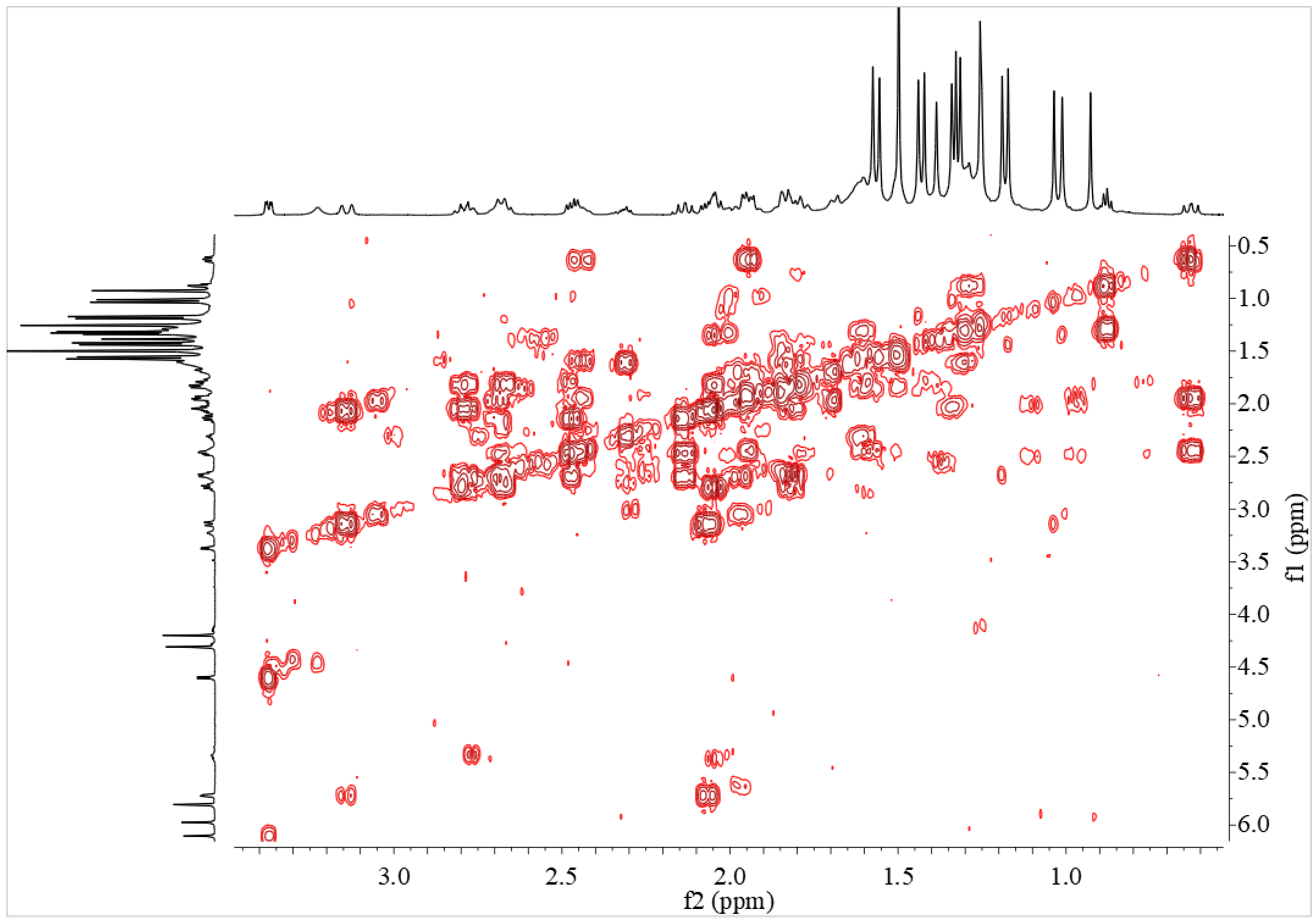


**Figure S16.** Partial ^1^H-^1^H COSY (CDCl_3_) spectrum of compound **1**


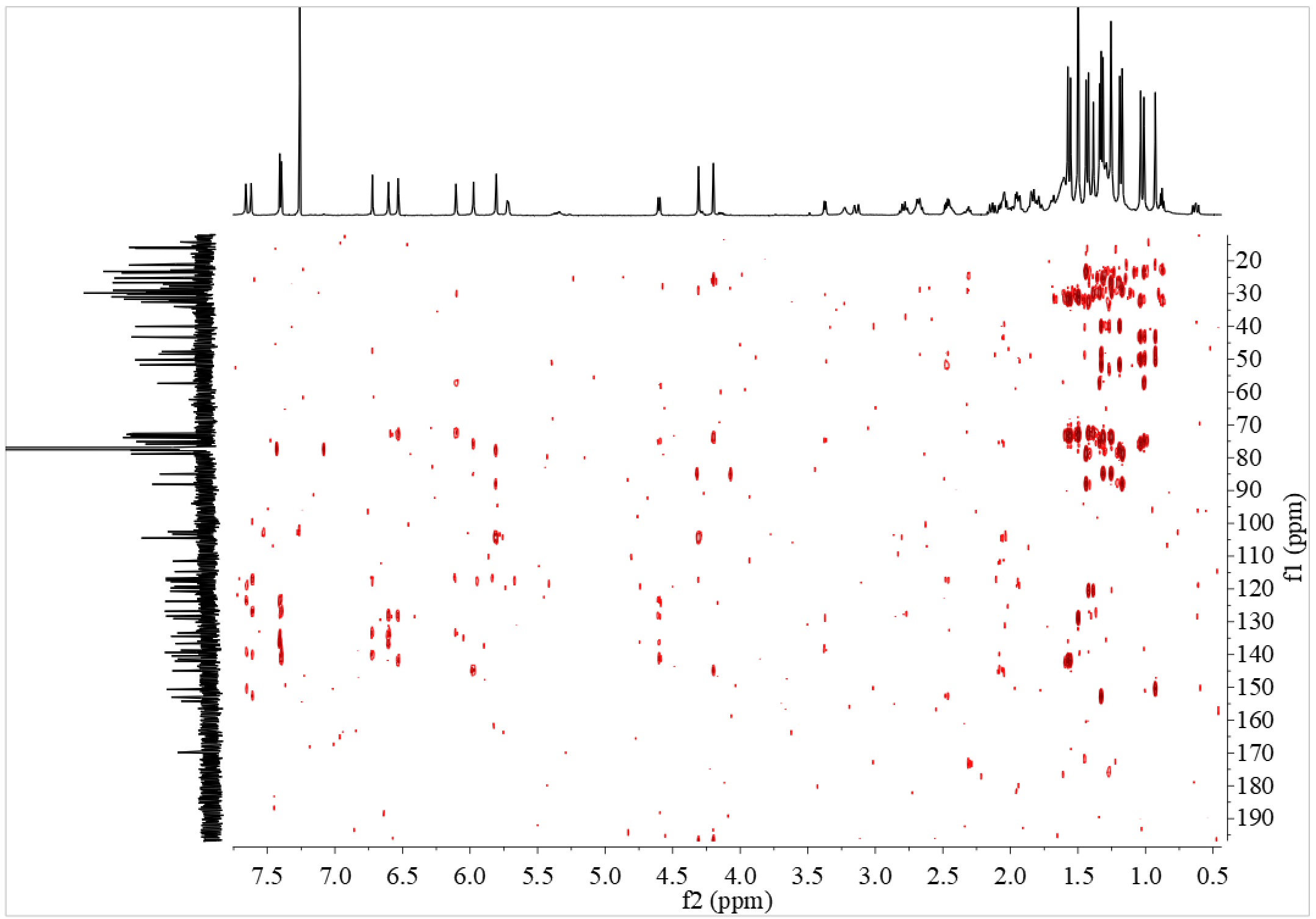


**Figure S17.** HMBC (CDCl_3_) spectrum of compound **1**


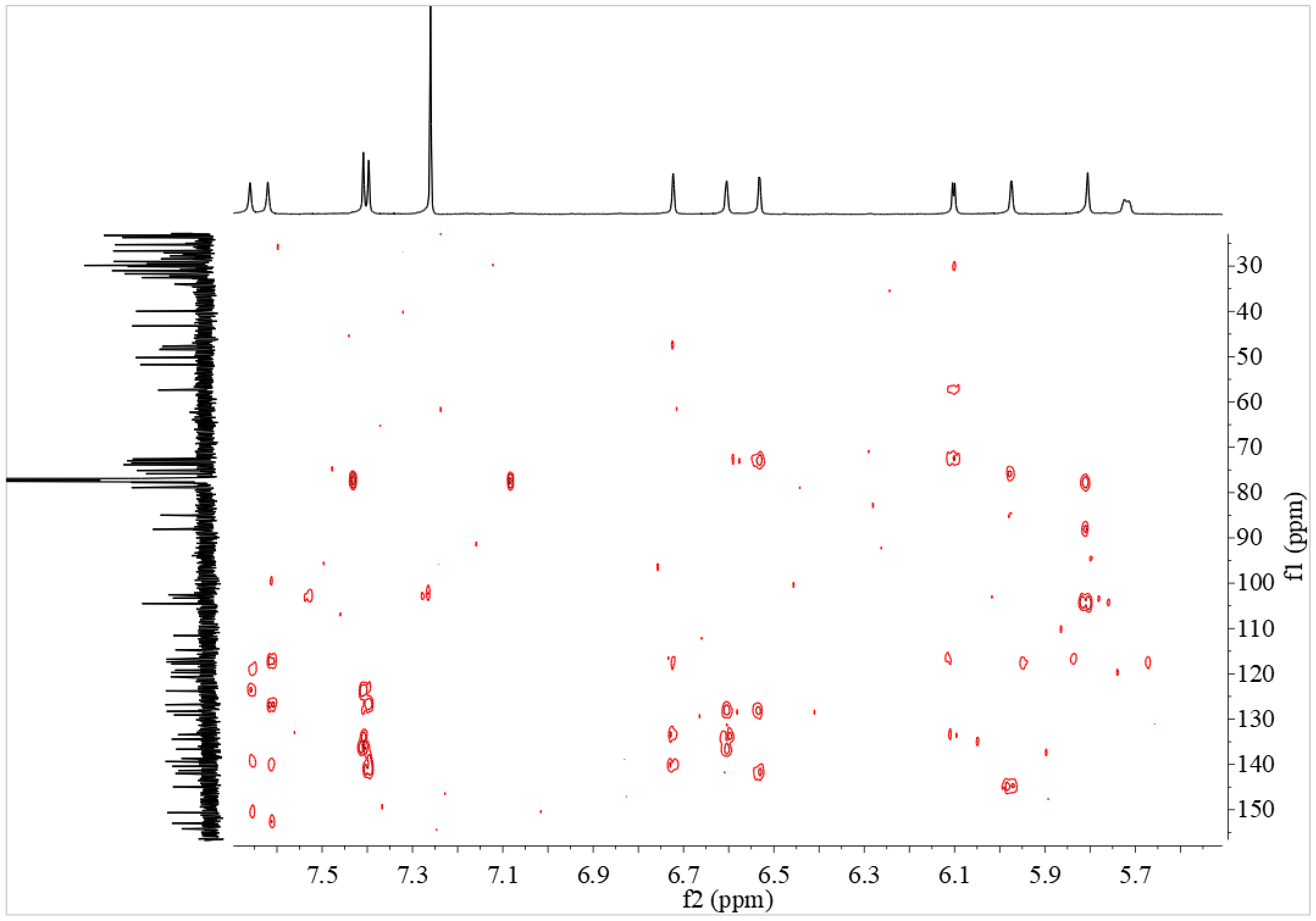


**Figure S18.** Partial HMBC (CDCl_3_) spectrum of compound **1**


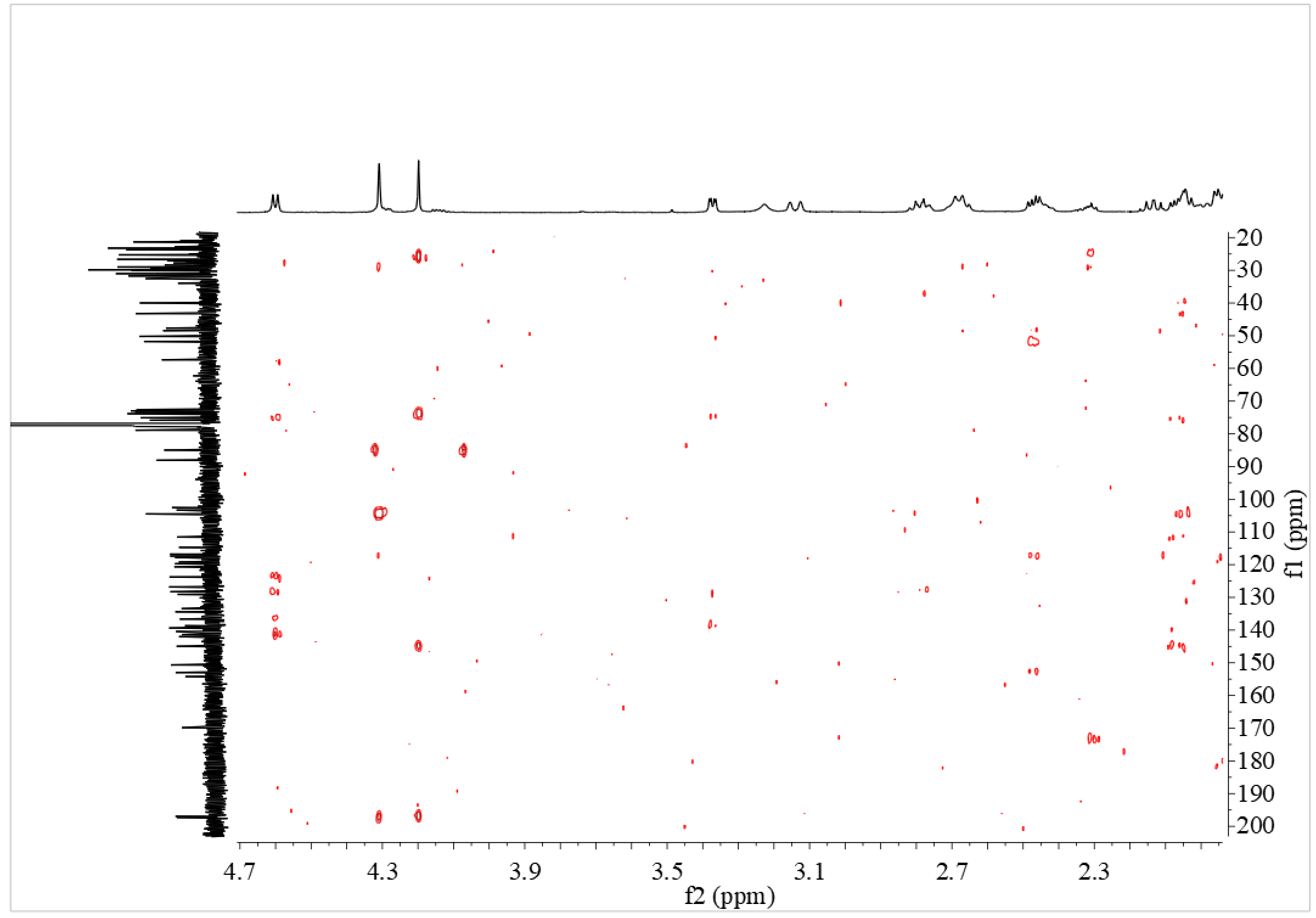


**Figure S19.** Partial HMBC (CDCl_3_) spectrum of compound **1**


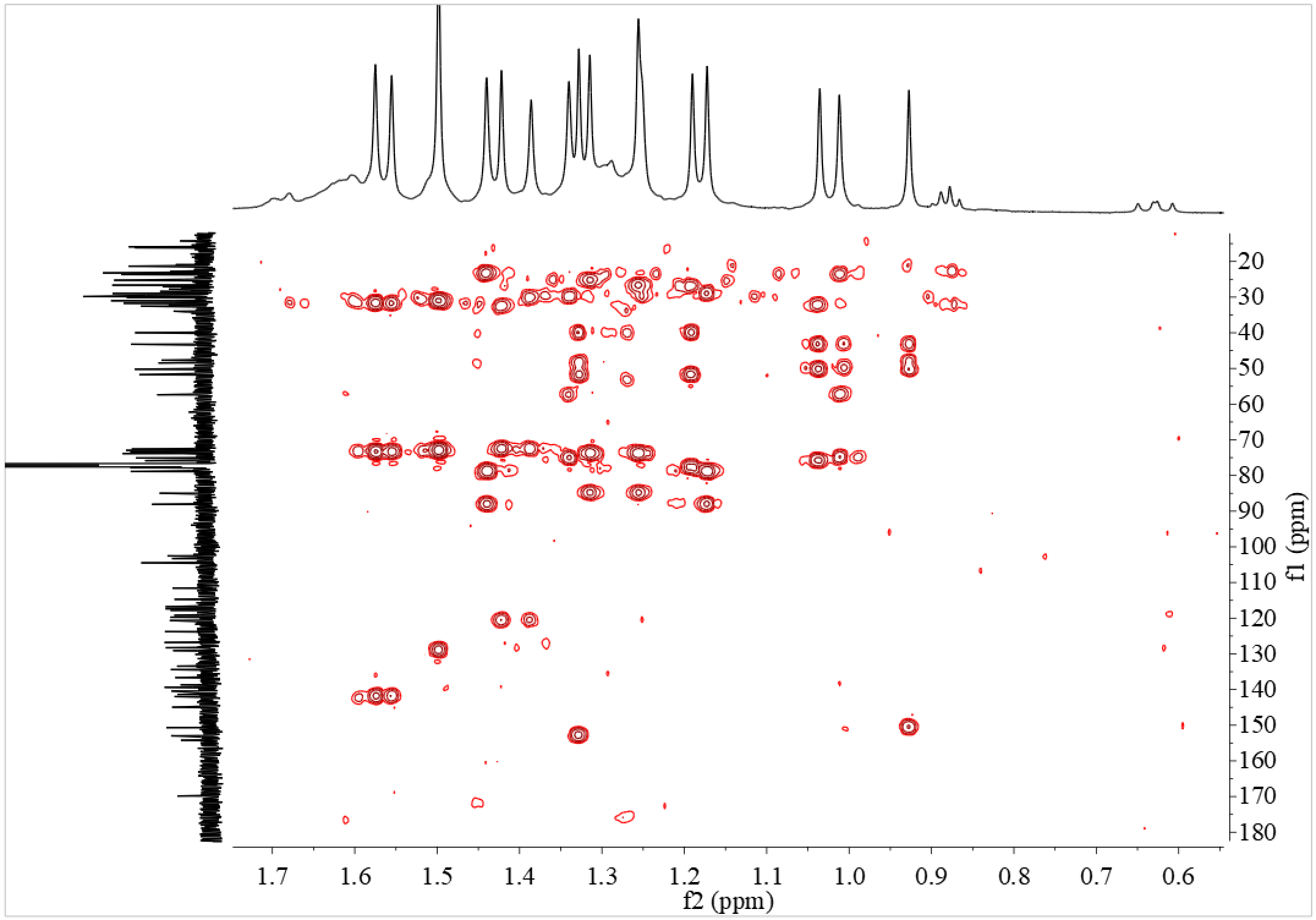


**Figure S20.** Partial HMBC (CDCl_3_) spectrum of compound **1**


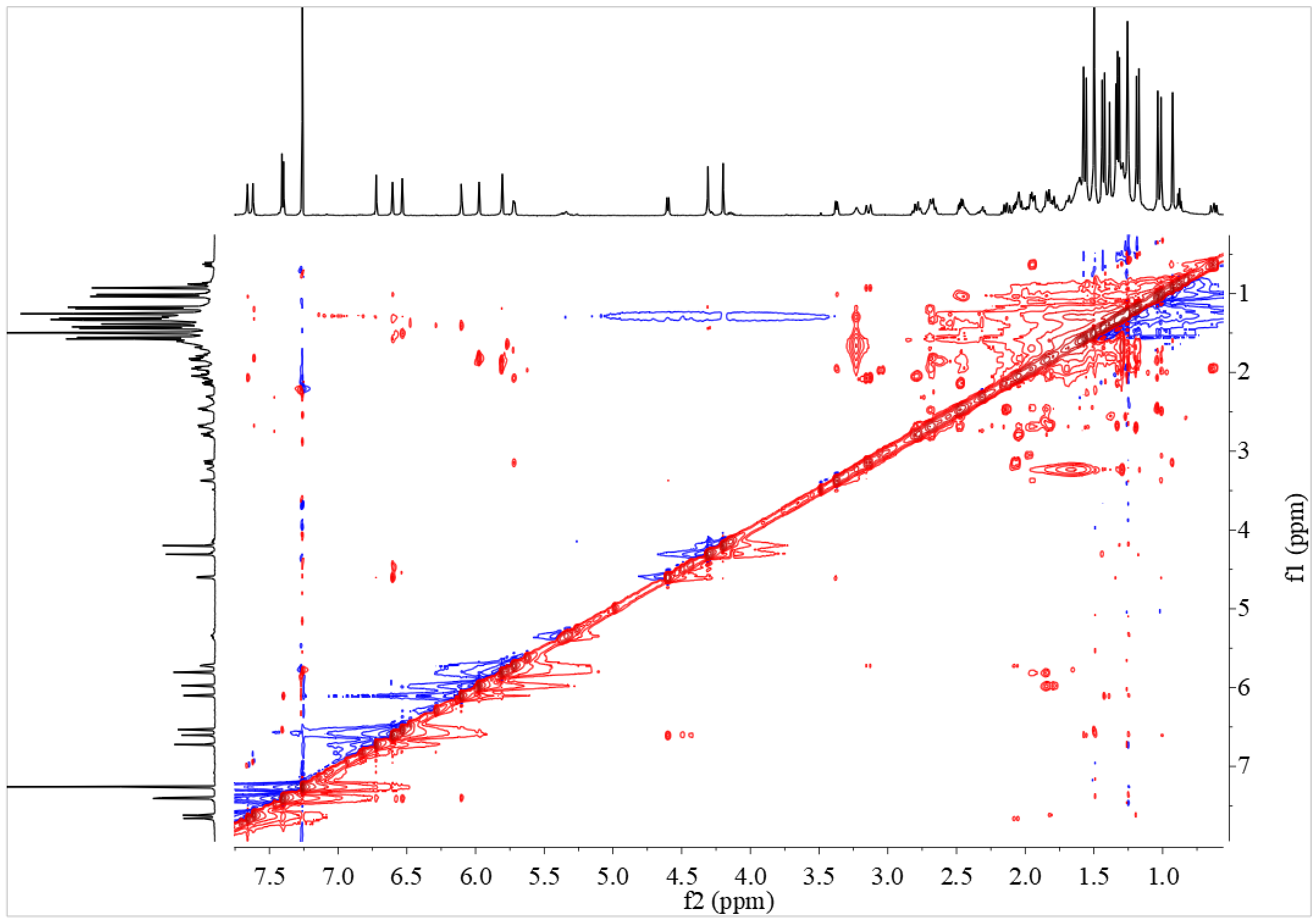


**Figure S21.** NOESY (CDCl_3_) spectrum of compound **1**


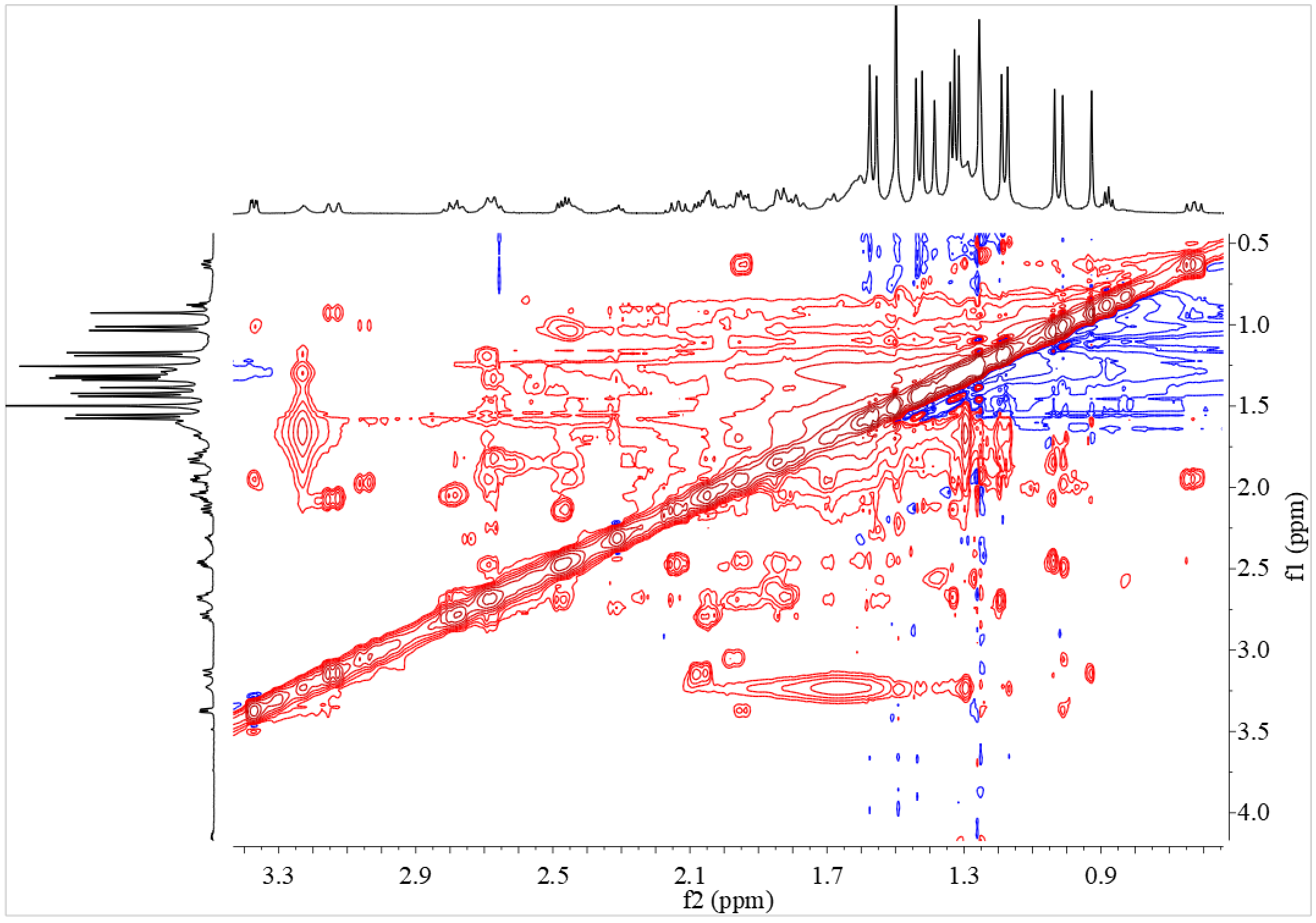


**Figure S22.** Partial NOESY (CDCl_3_) spectrum of compound **1**


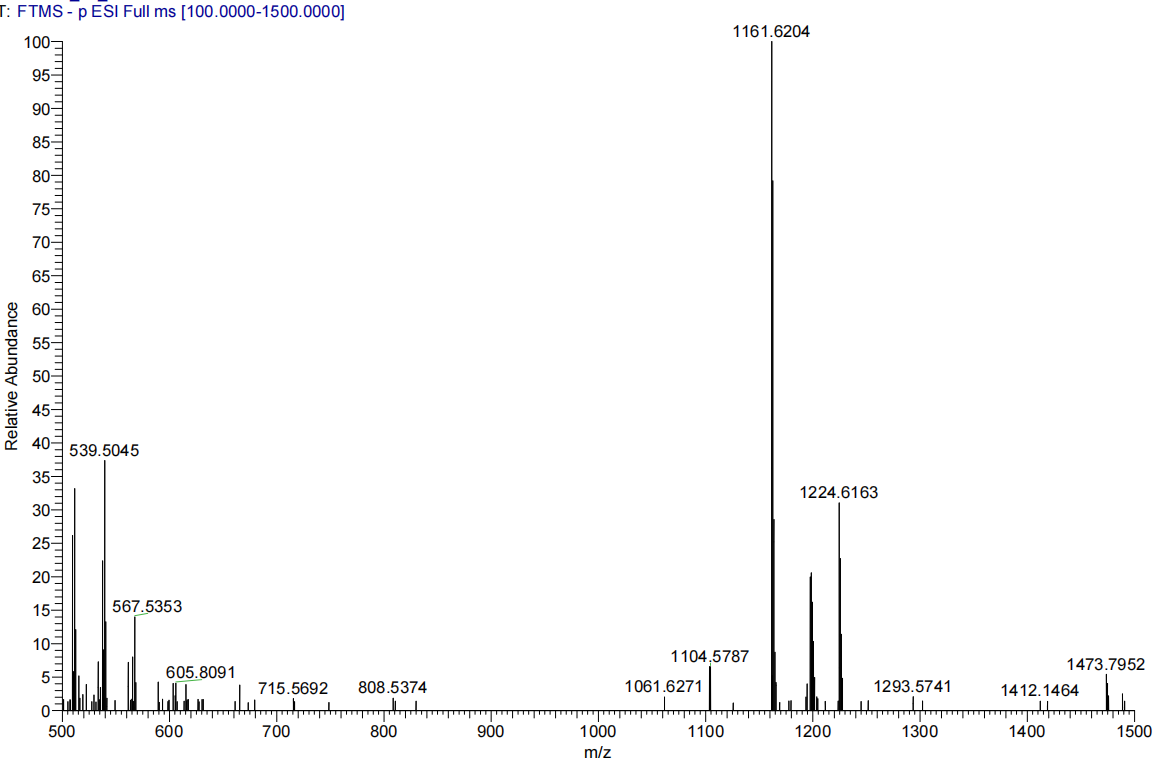


**Figure S23.** HRESIMS spectrum of compound **1**


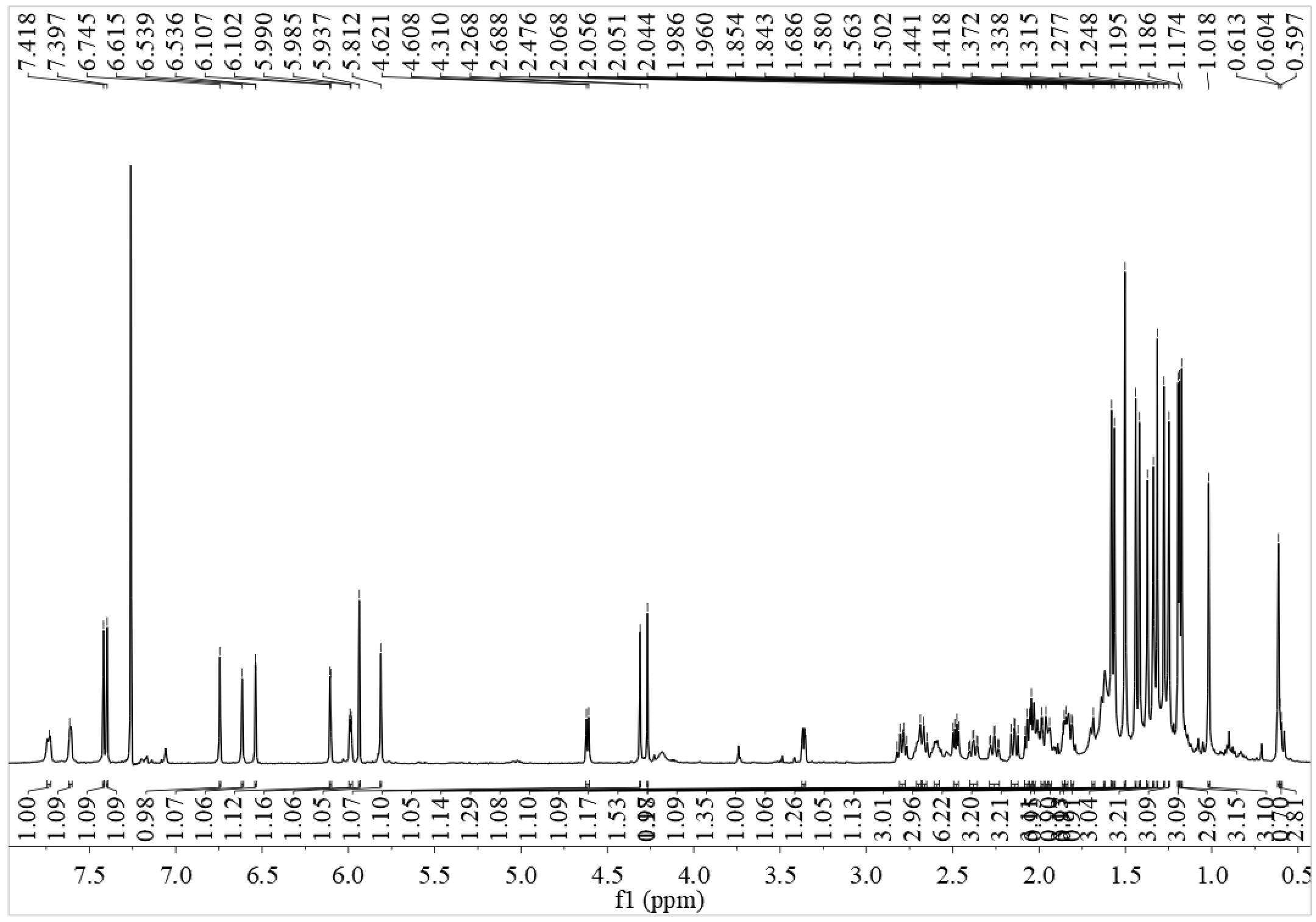


**Figure S24.** ^1^H NMR (600 MHz, CDCl_3_) spectrum of compound **2**


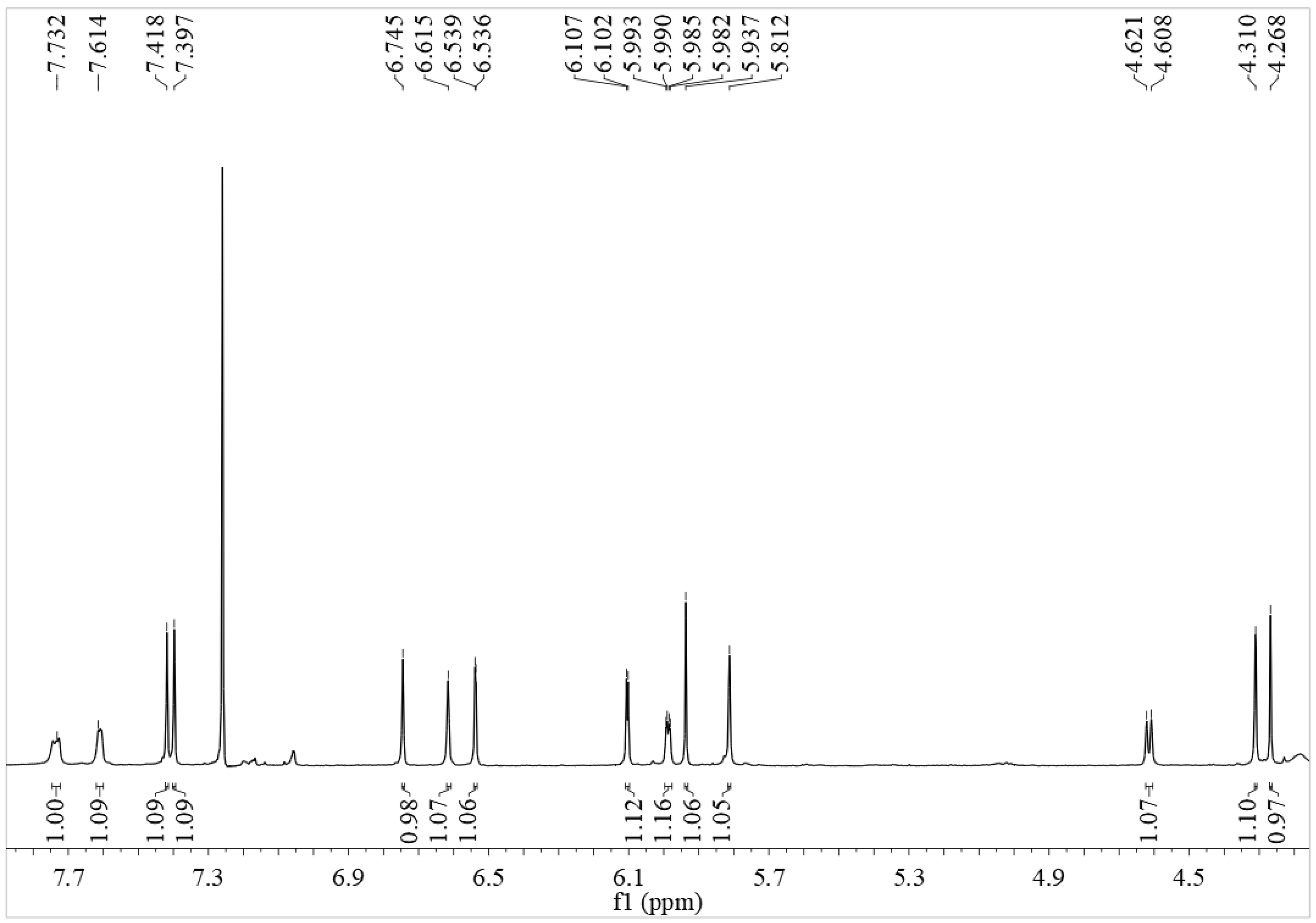


**Figure S25.** Partial ^1^H NMR (600 MHz, CDCl_3_) spectrum of compound **2**


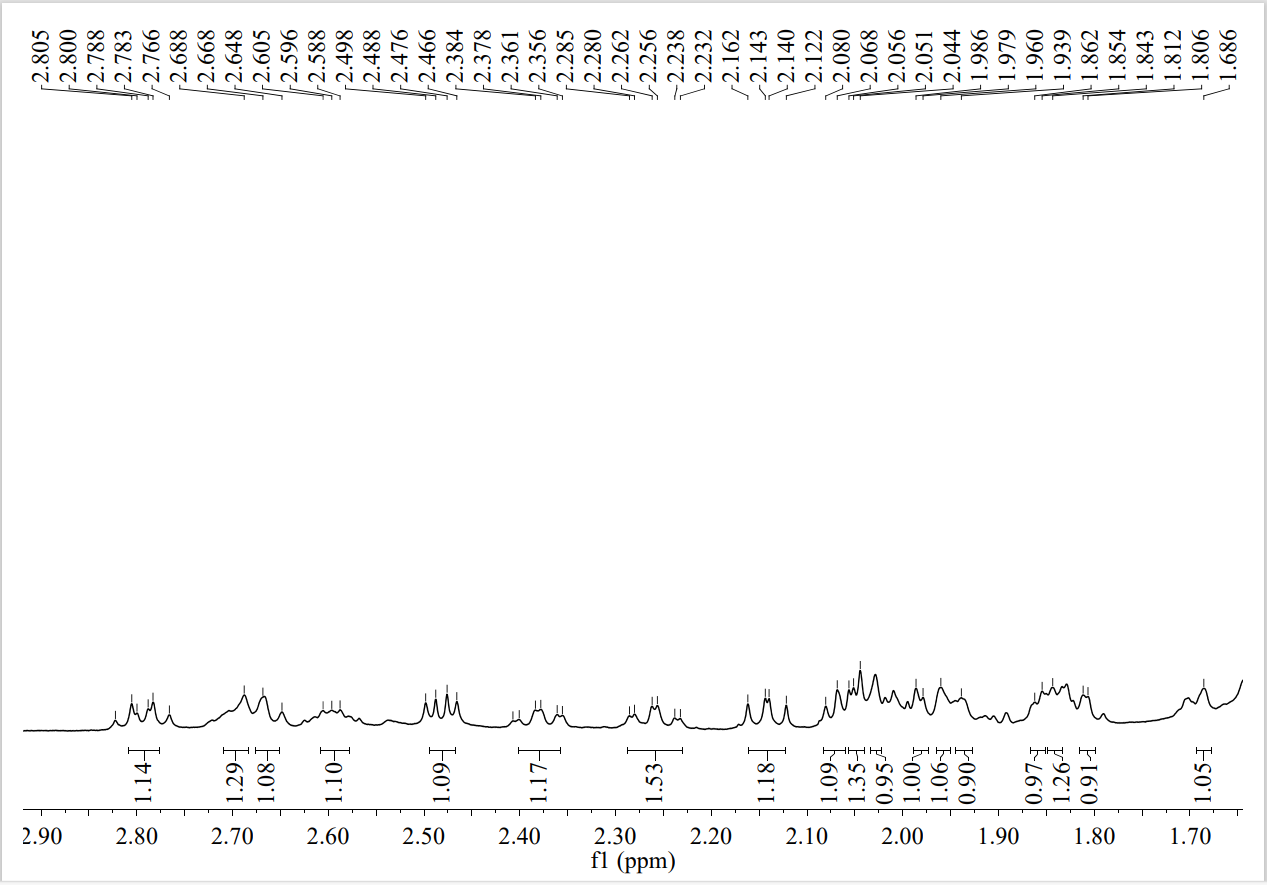


**Figure S26.** Partial ^1^H NMR (600 MHz, CDCl_3_) spectrum of compound **2**


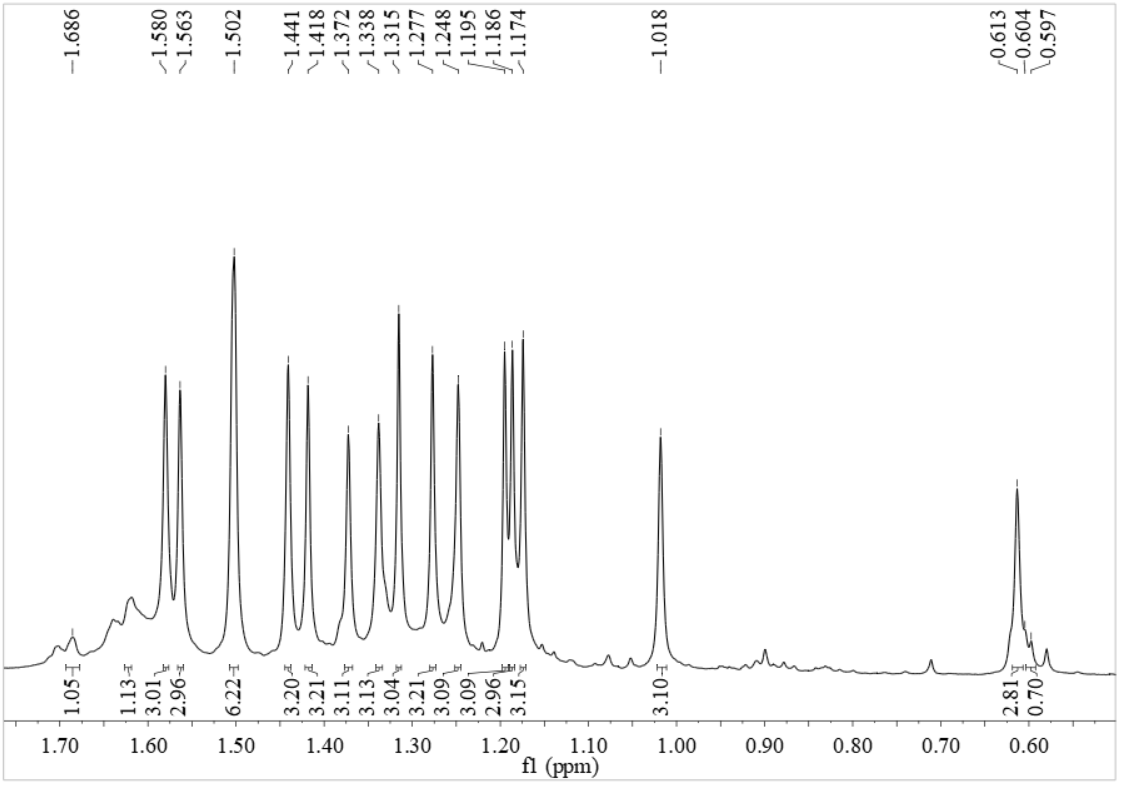


**Figure S27.** Partial ^1^H NMR (600 MHz, CDCl_3_) spectrum of compound **2**


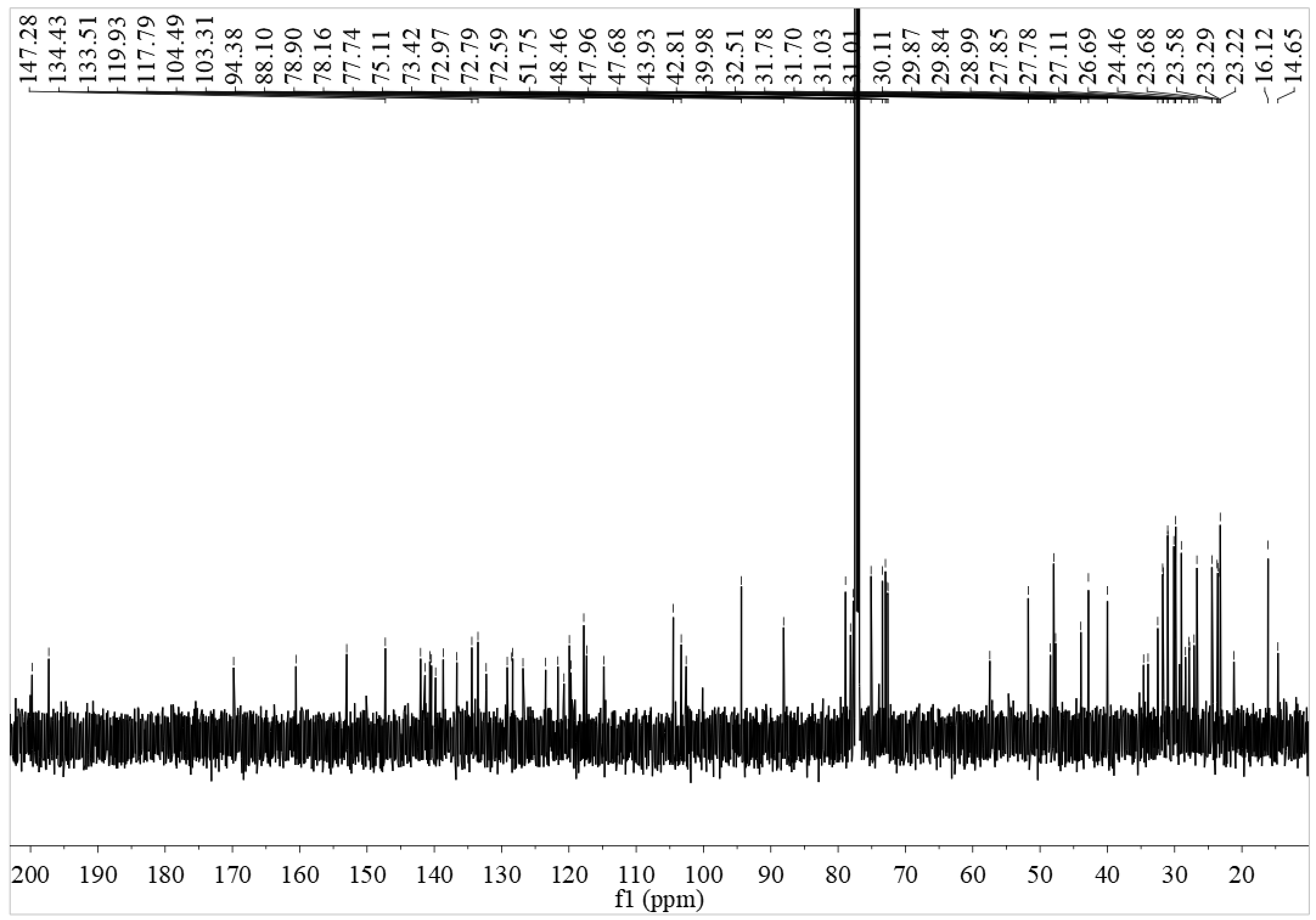


**Figure S28.** ^13^C NMR (150 MHz, CDCl_3_) spectrum of compound **2**


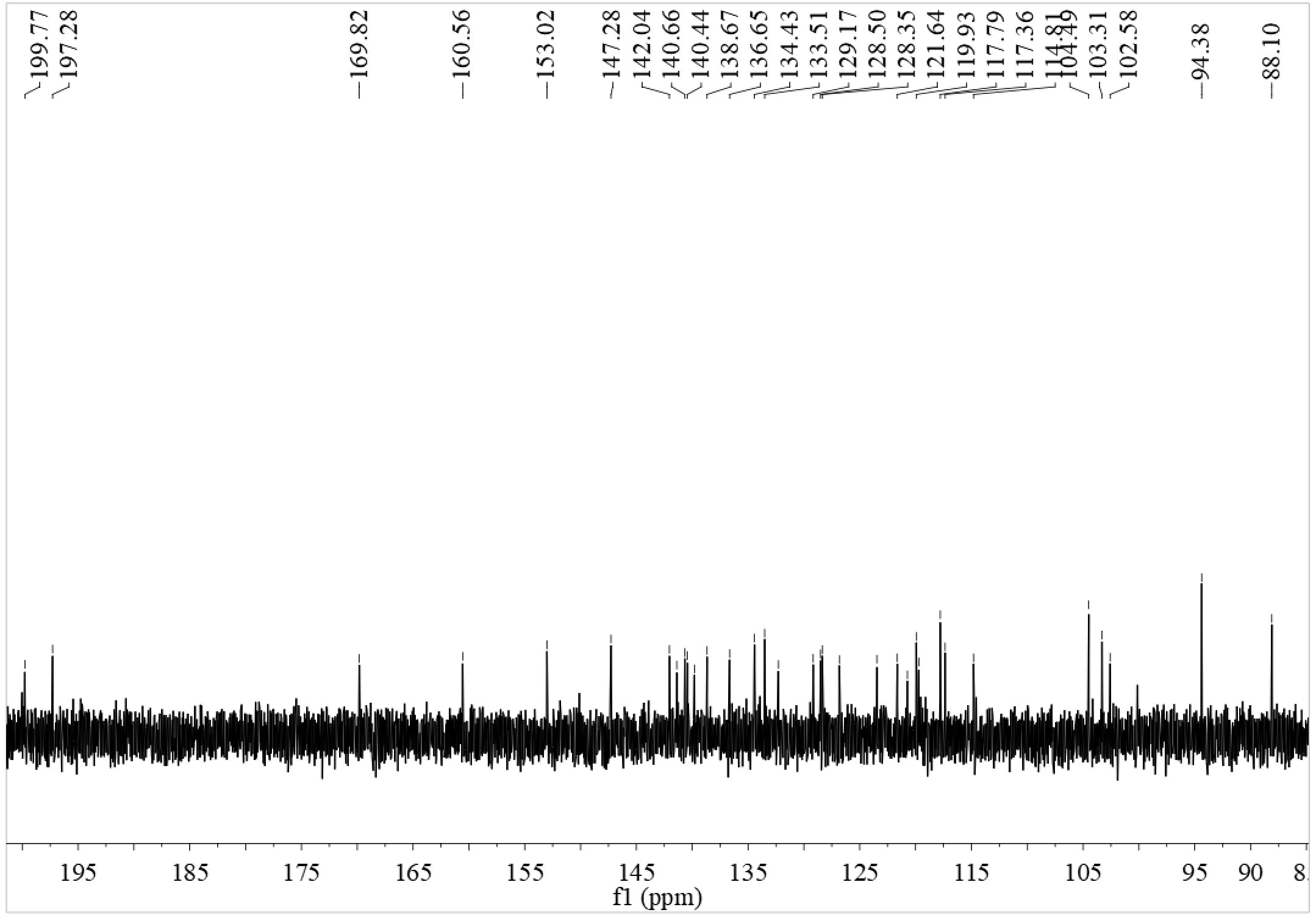


**Figure S29.** Partial ^13^C NMR (150 MHz, CDCl_3_) spectrum of compound **2**


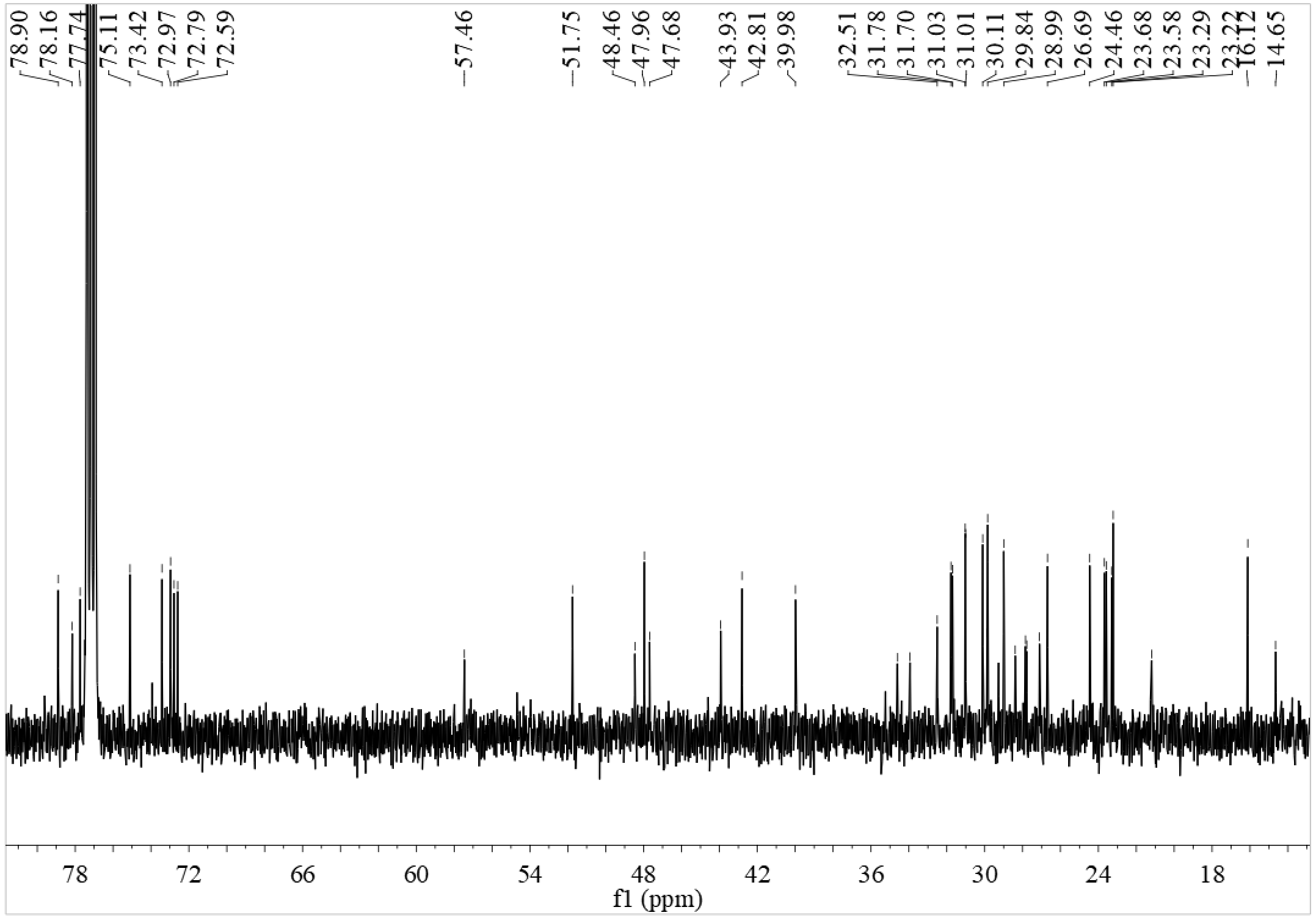


**Figure S30.** Partial ^13^C NMR (150 MHz, CDCl_3_) spectrum of compound **2**


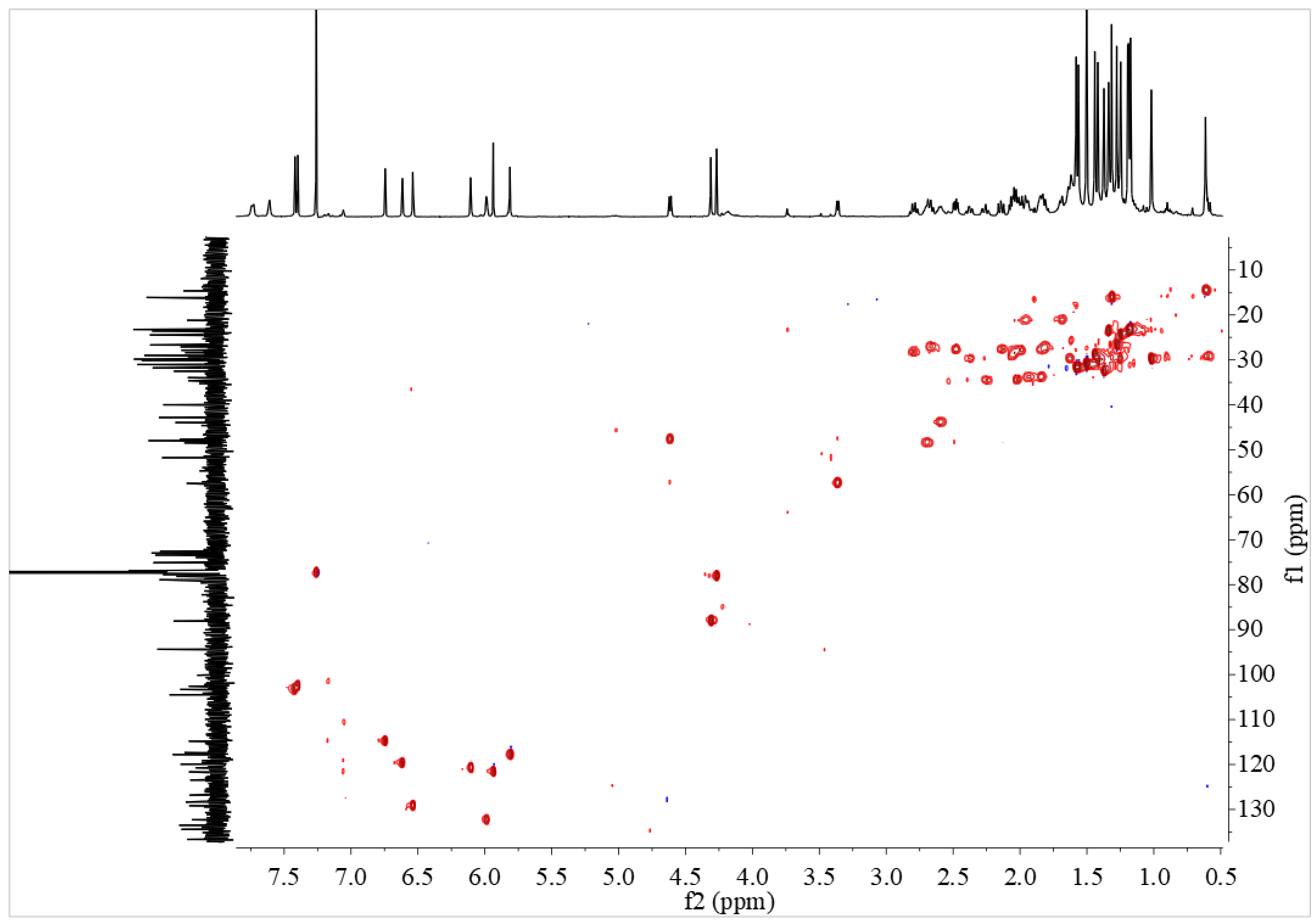


**Figure S31.** HSQC (CDCl_3_) spectrum of compound **2**


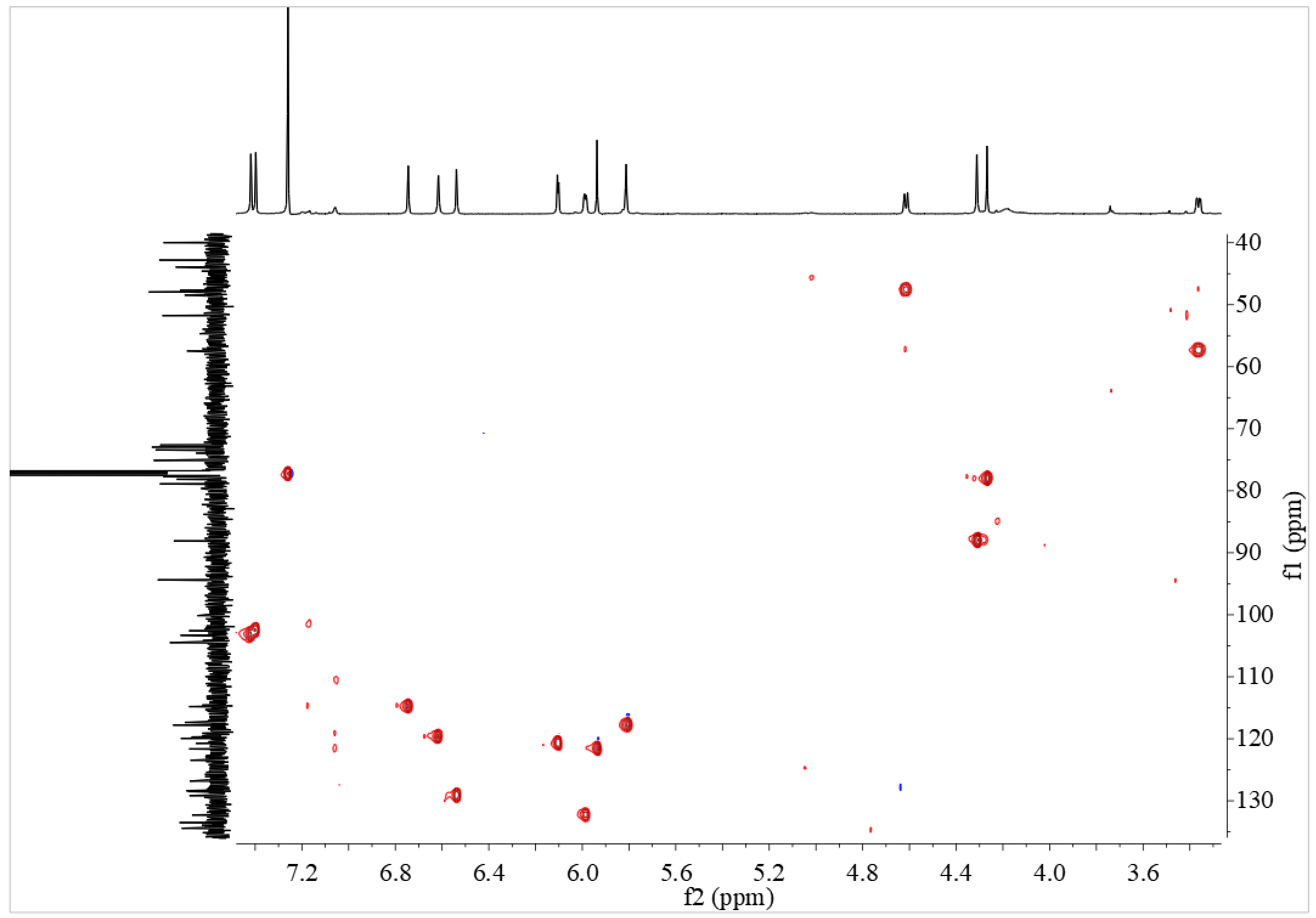


**Figure S32.** Partial HSQC (CDCl_3_) spectrum of compound **2**


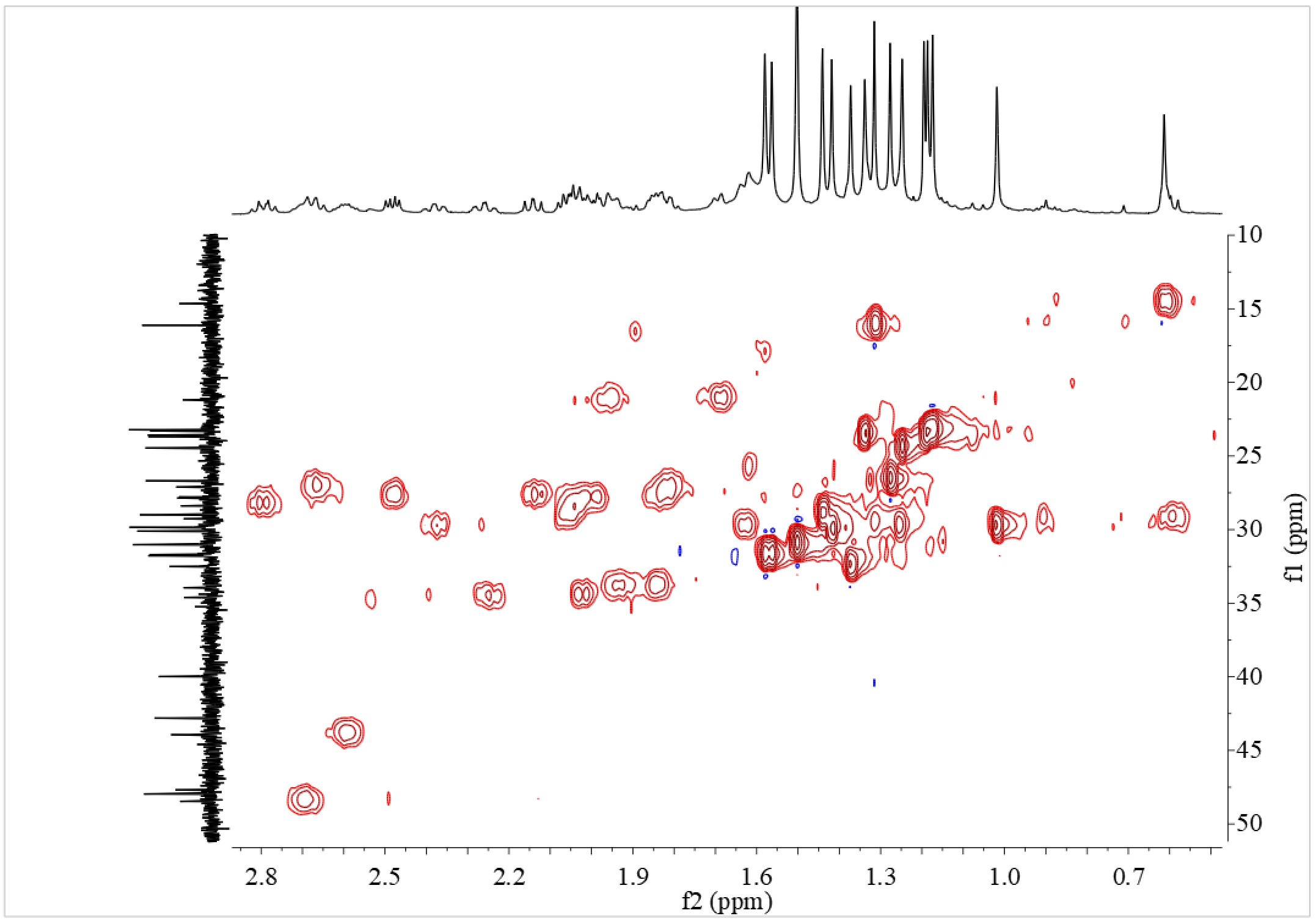


**Figure S33.** Partial HSQC (CDCl_3_) spectrum of compound **2**


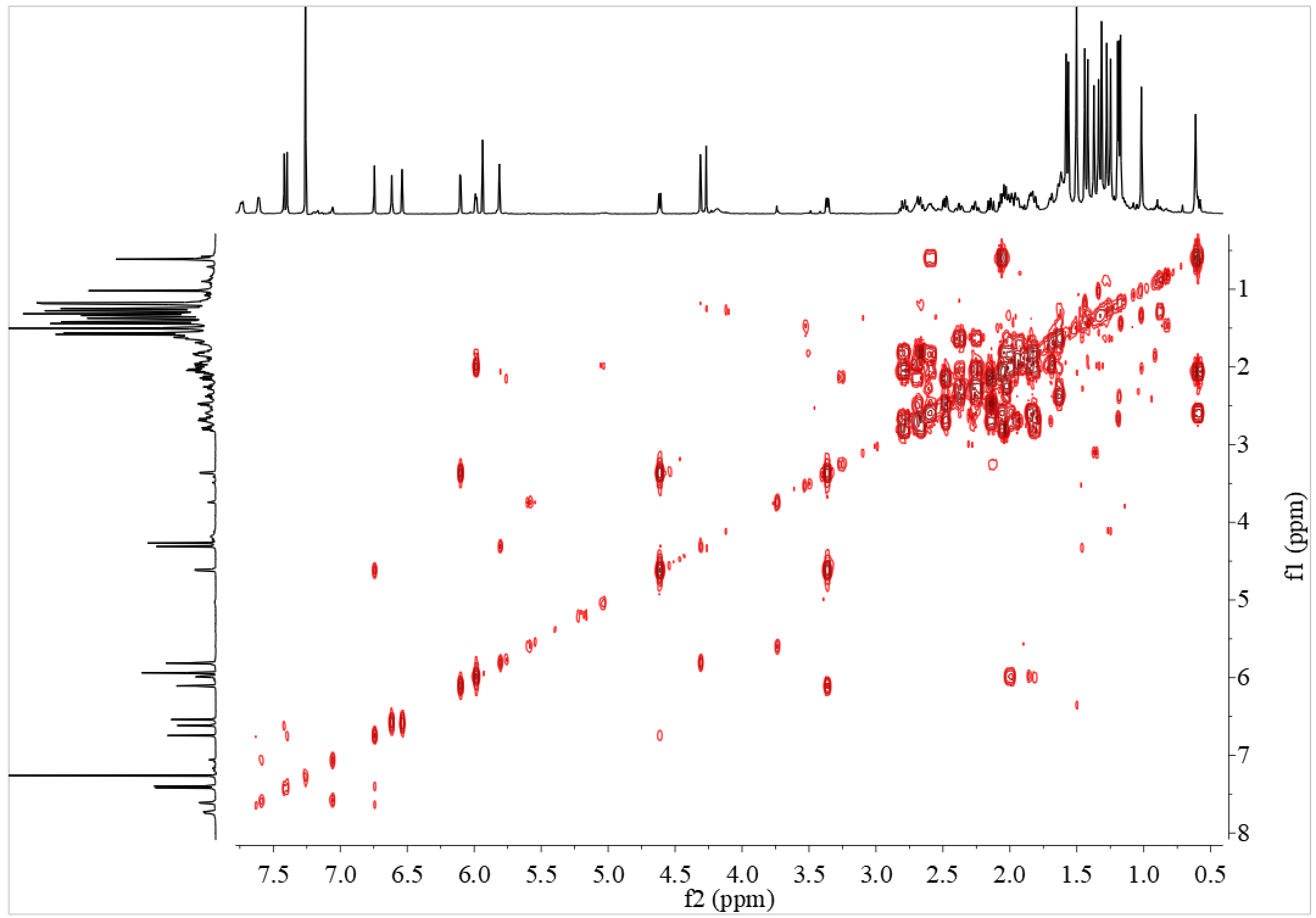


**Figure S34.** ^1^H-^1^H COSY (CDCl_3_) spectrum of compound **2**


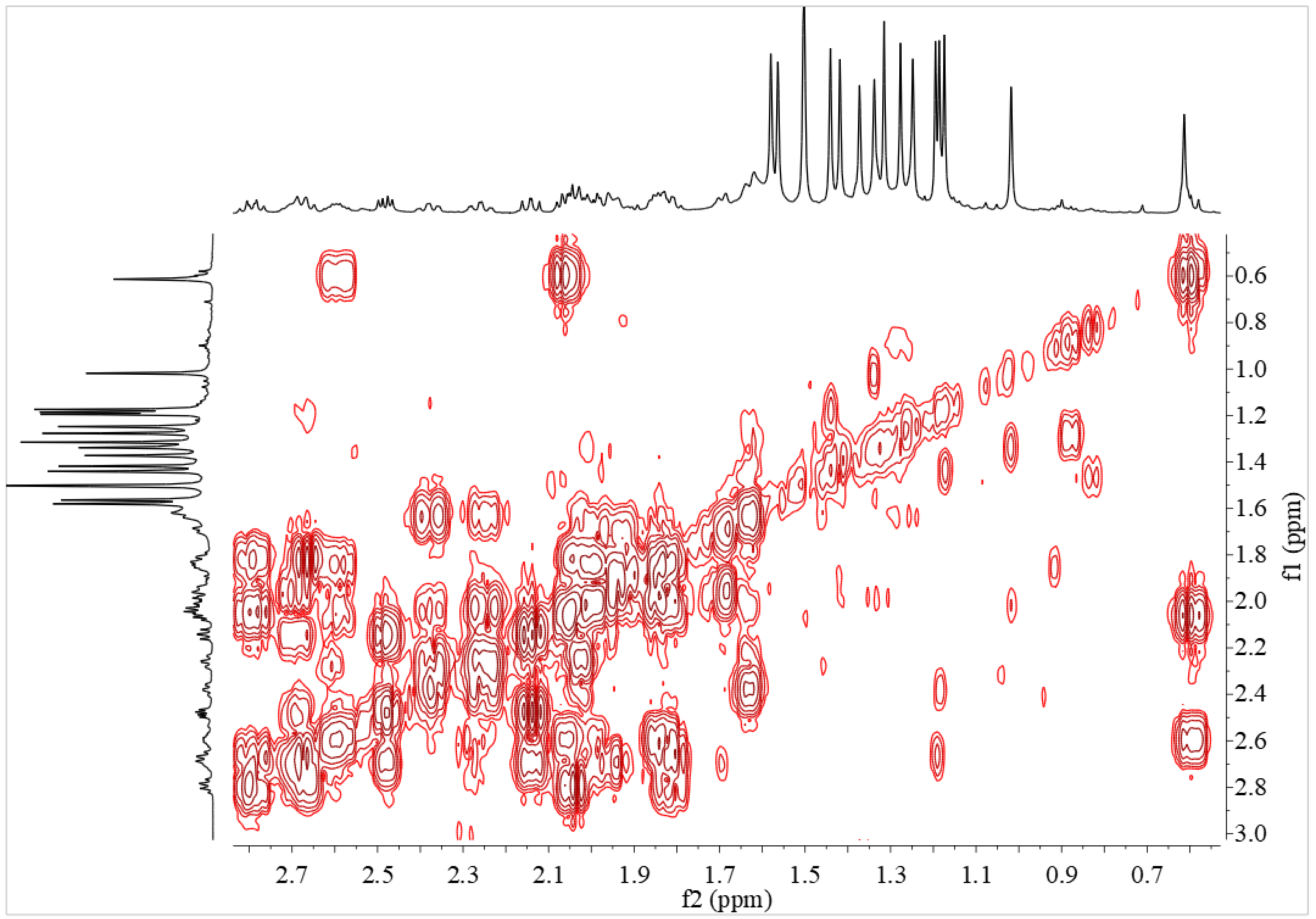


**Figure S35.** Partial ^1^H-^1^H COSY (CDCl_3_) spectrum of compound **2**


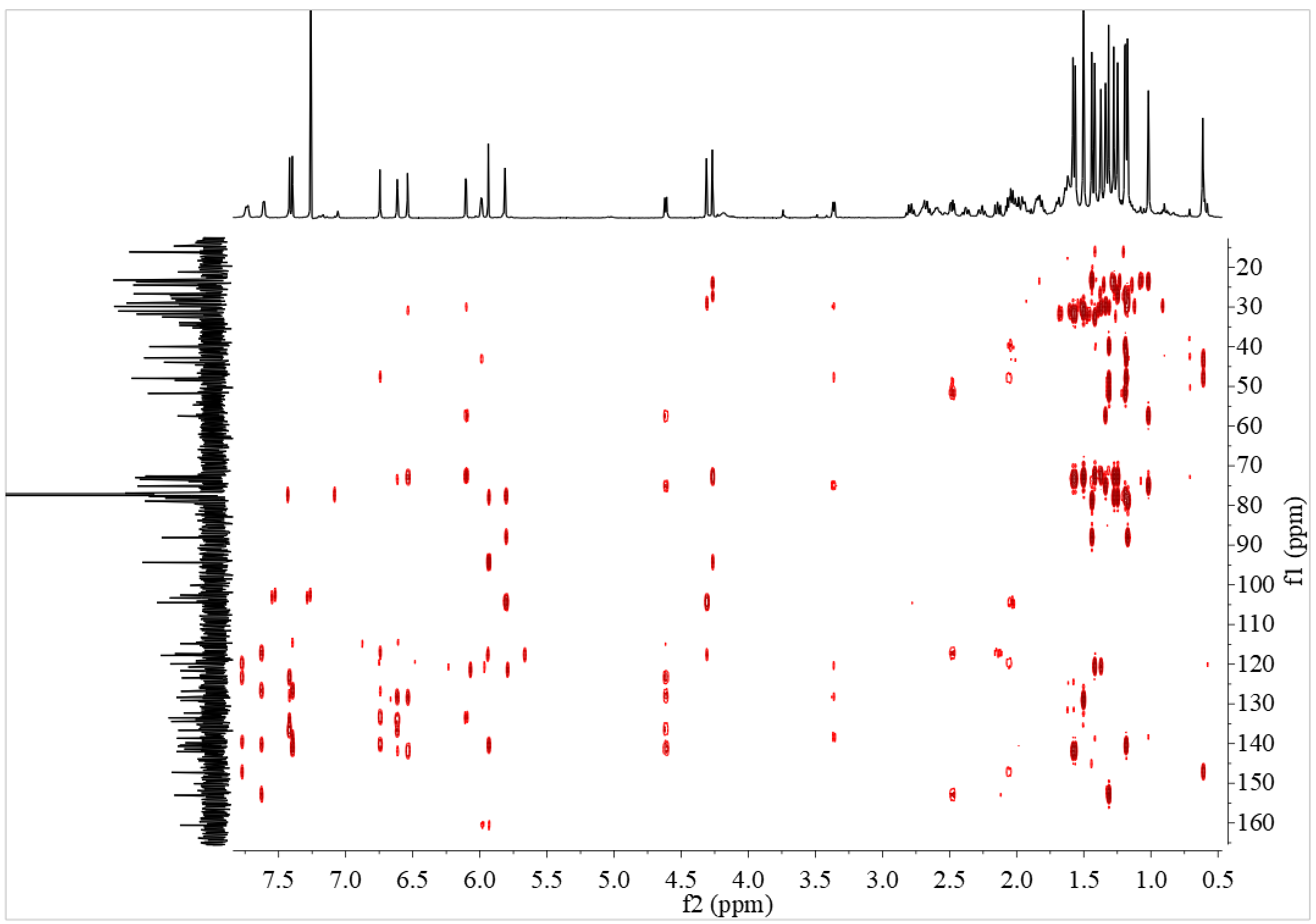


**Figure S36.** HMBC (CDCl_3_) spectrum of compound **2**


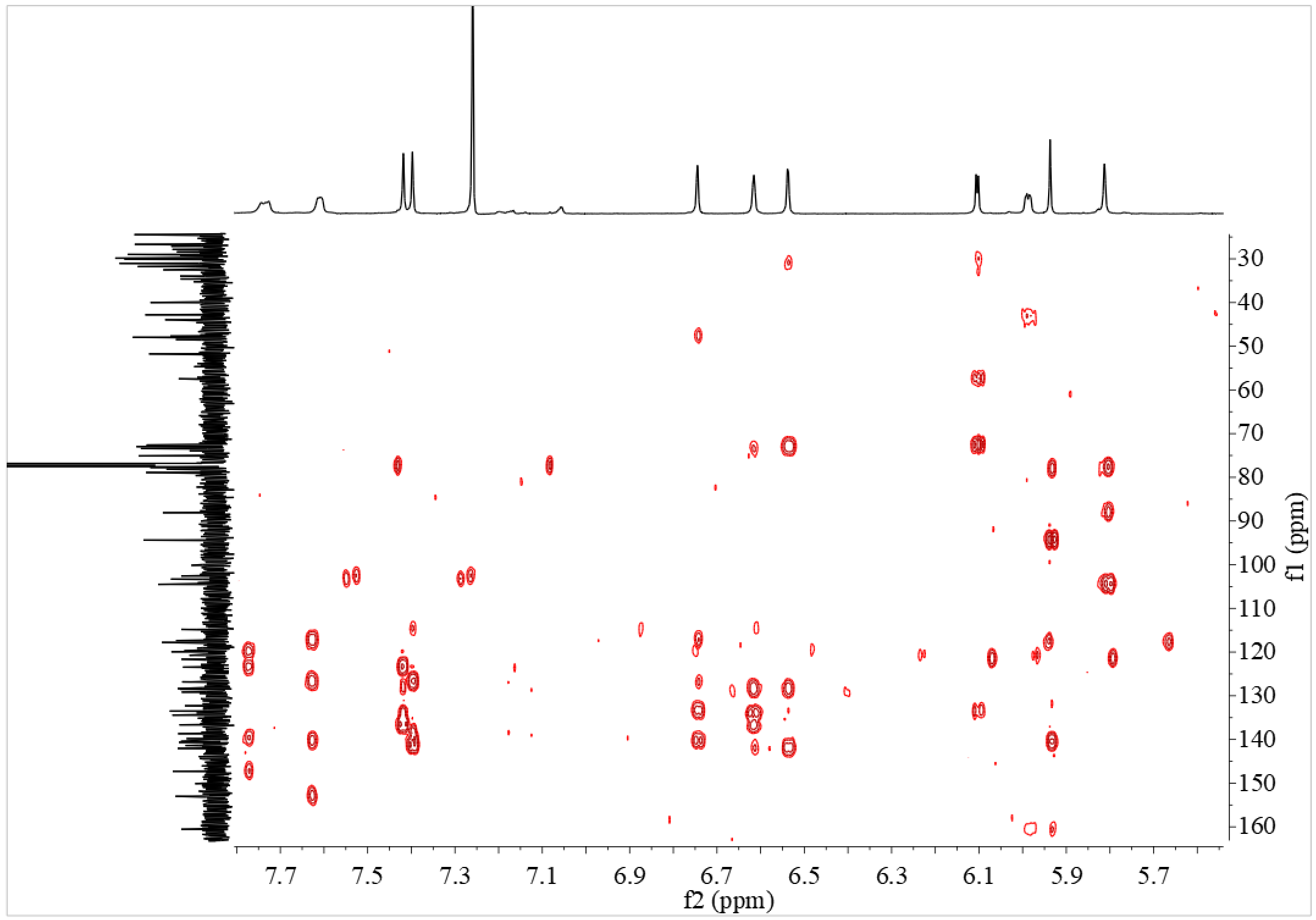


**Figure S37.** Partial HMBC (CDCl_3_) spectrum of compound **2**


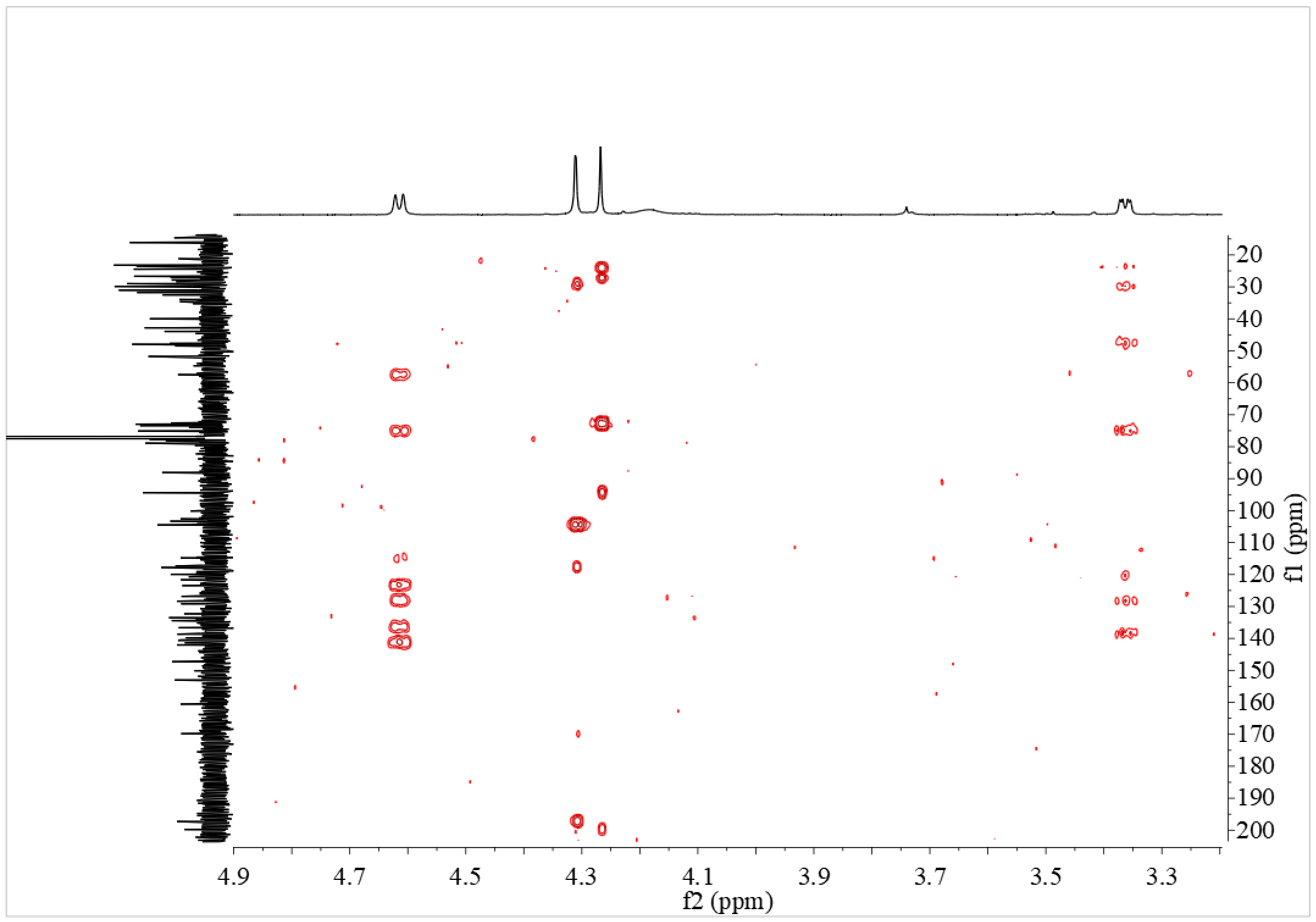


**Figure S38.** Partial HMBC (CDCl_3_) spectrum of compound **2**


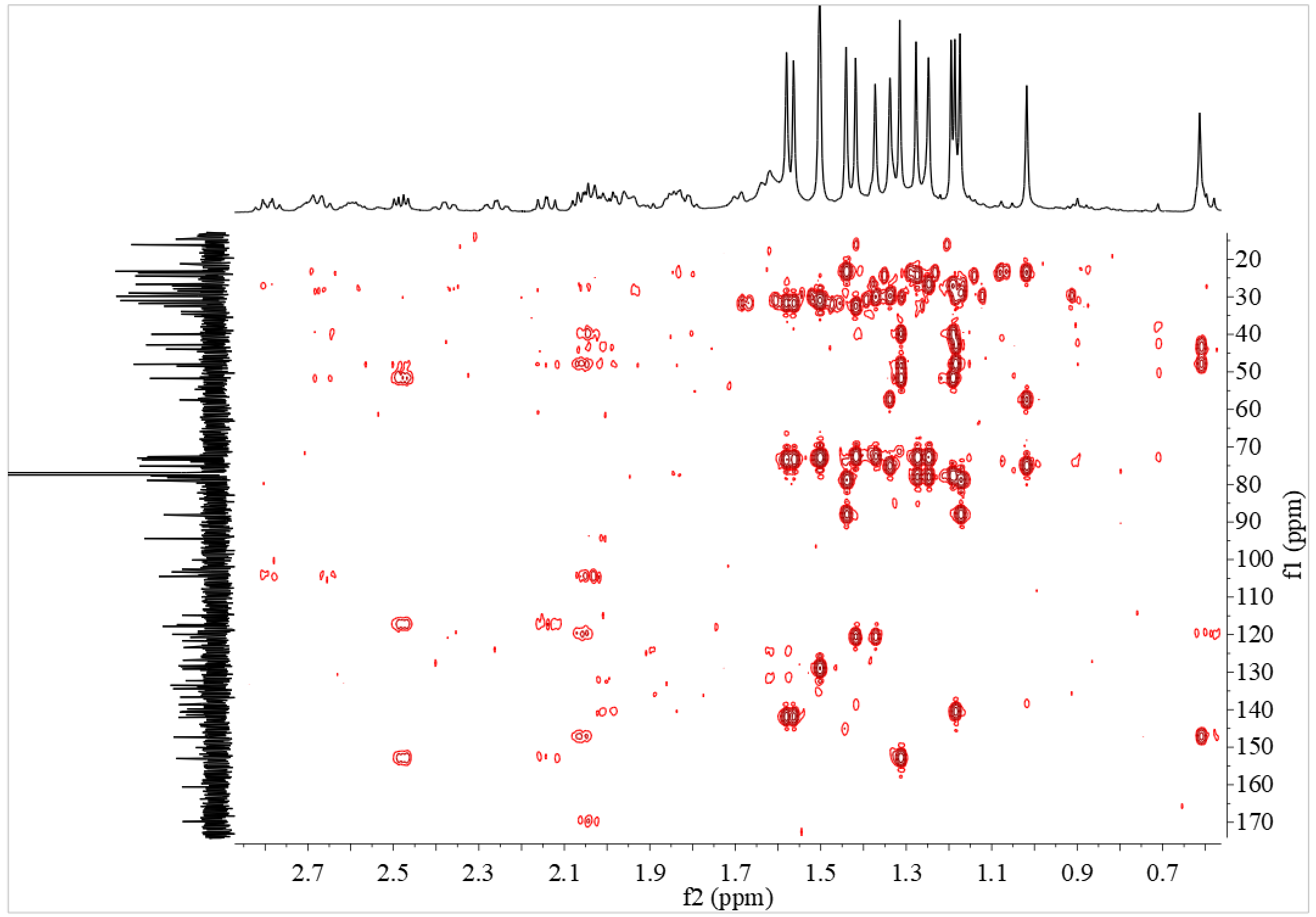


**Figure S39.** Partial HMBC (CDCl_3_) spectrum of compound **2**


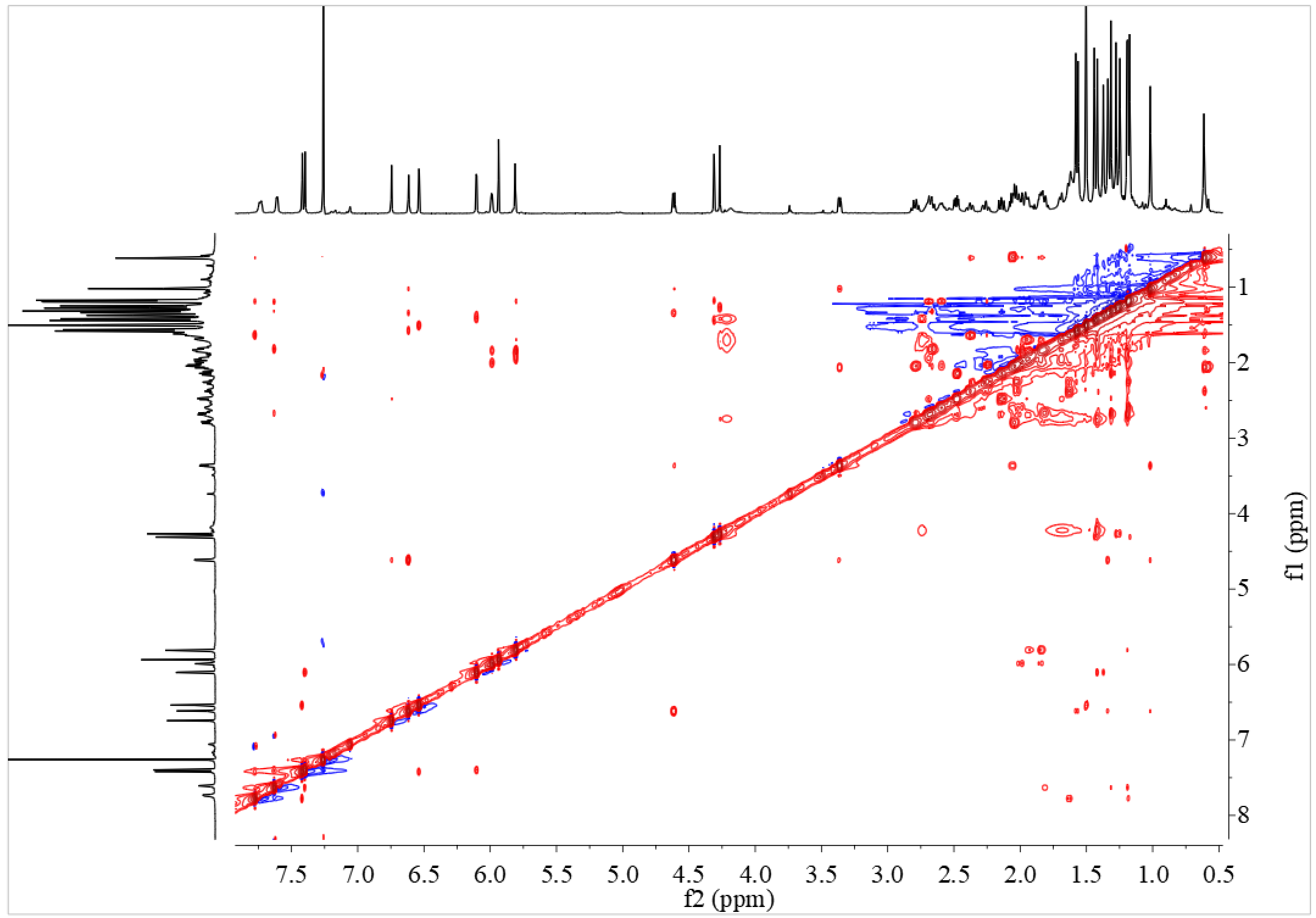


**Figure S40.** NOESY (CDCl_3_) spectrum of compound **2**


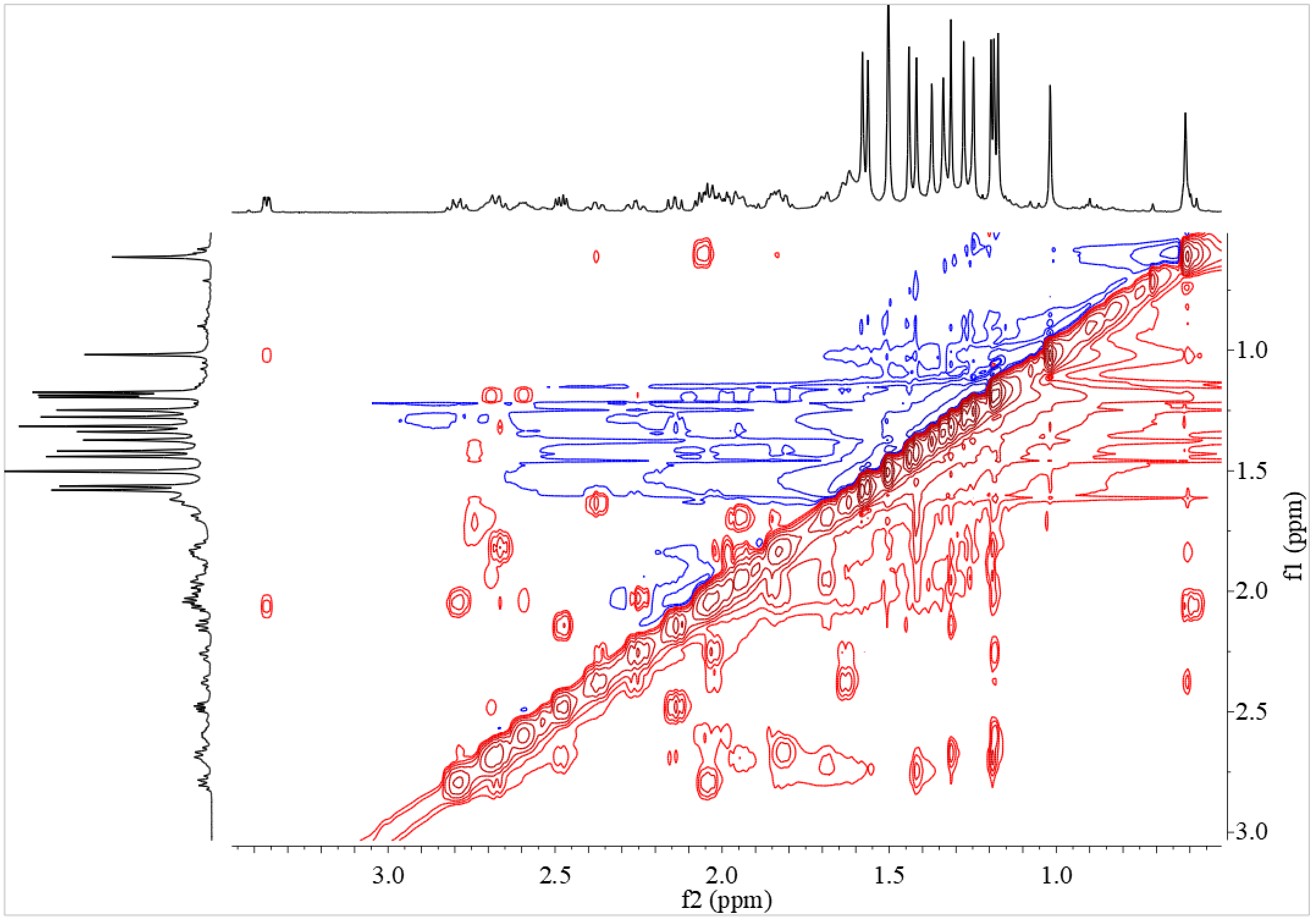


**Figure S41.** Partial NOESY (CDCl_3_) spectrum of compound **2**


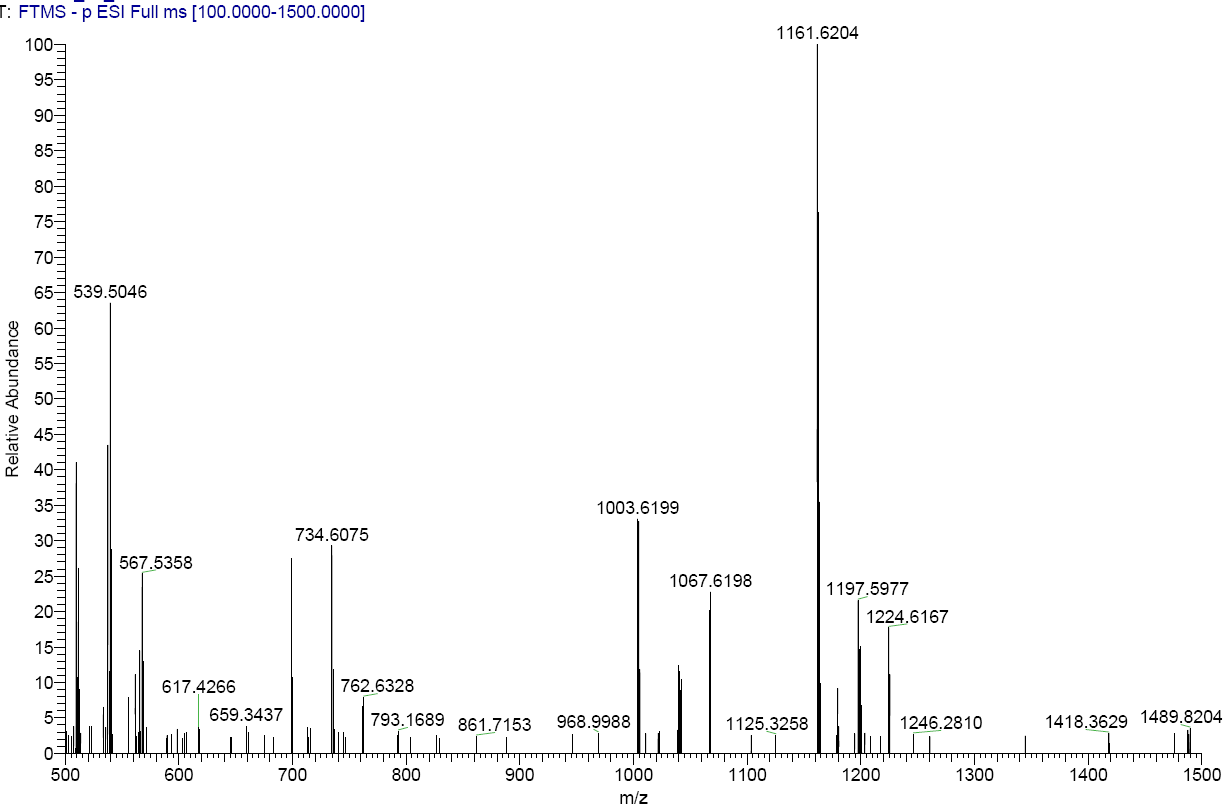


**Figure S42.** HRESIMS spectrum of compound **2**


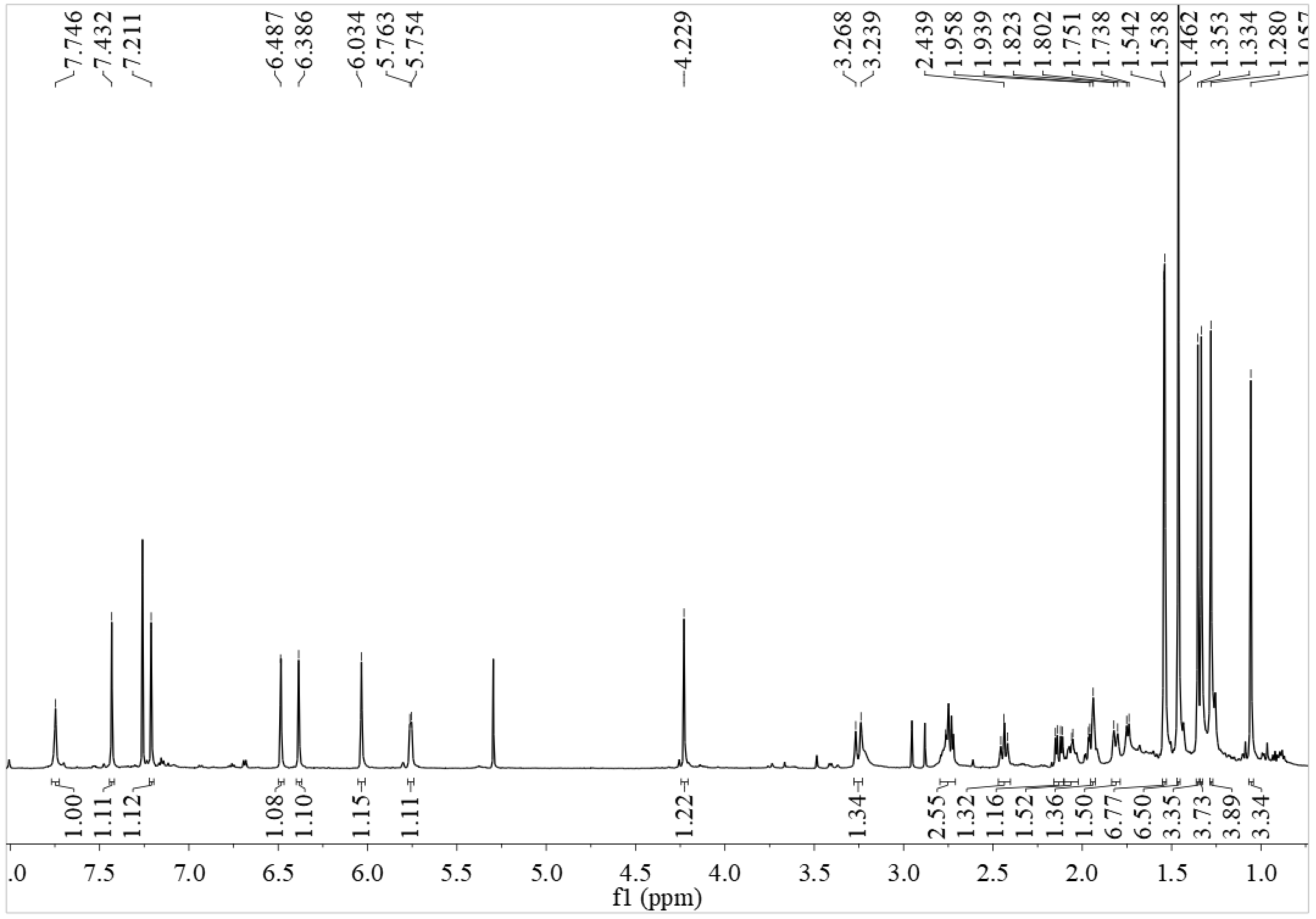


**Figure S43.** ^1^H NMR (600 MHz, CDCl_3_) spectrum of compound **3**


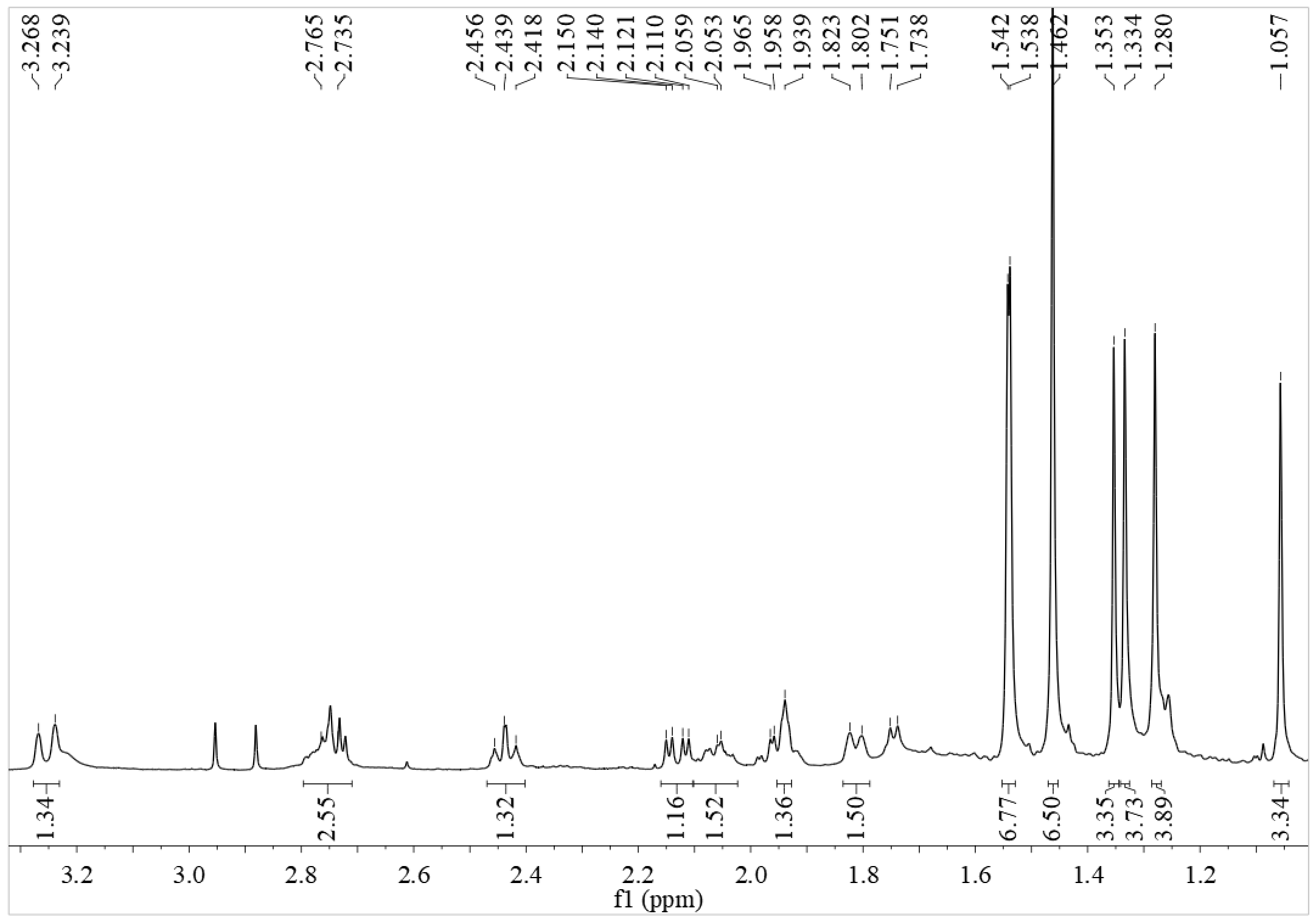


**Figure S44.** Partial ^1^H NMR (600 MHz, CDCl_3_) spectrum of compound **3**


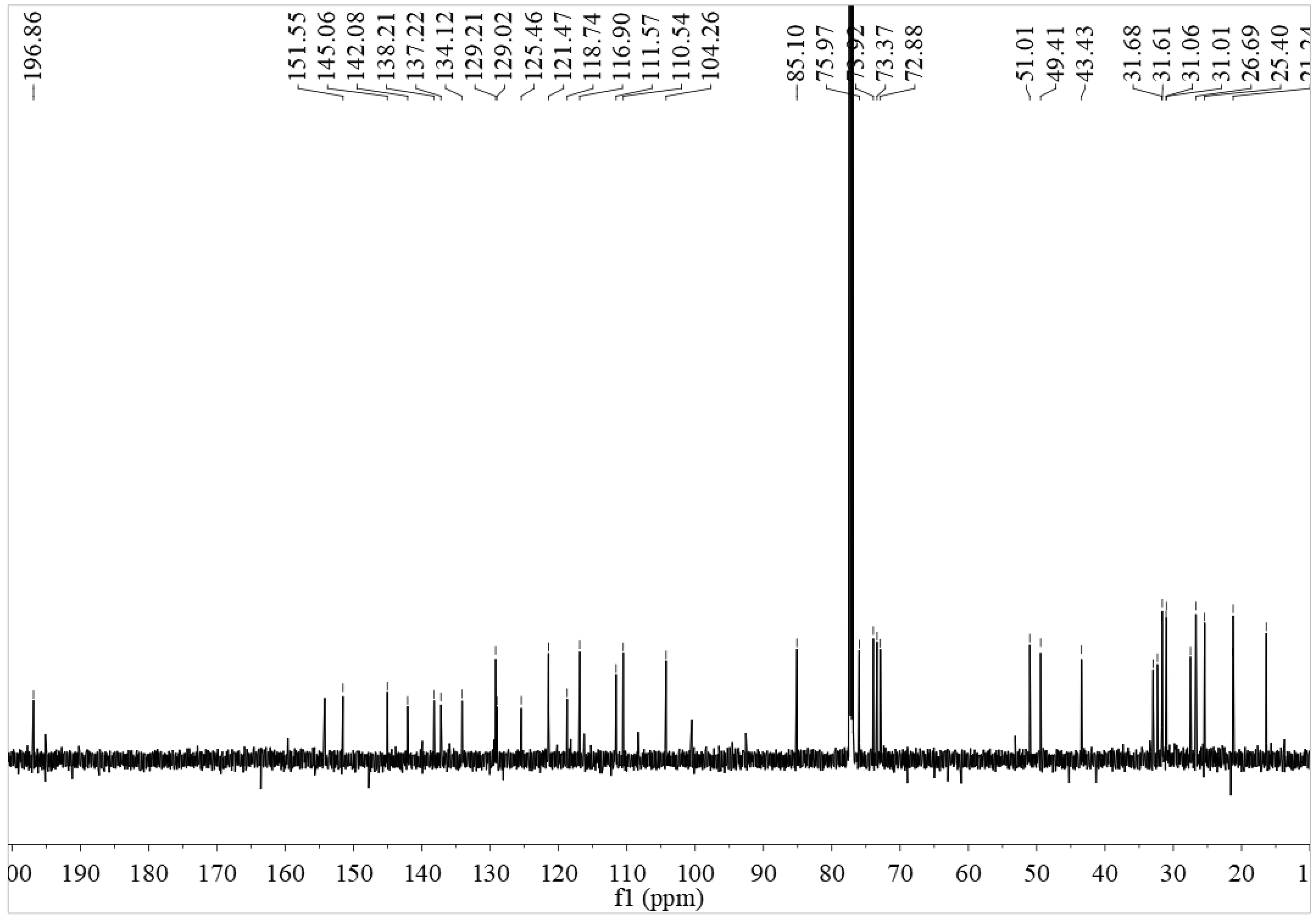


**Figure S45.** ^13^C NMR (150 MHz, CDCl_3_) spectrum of compound **3**


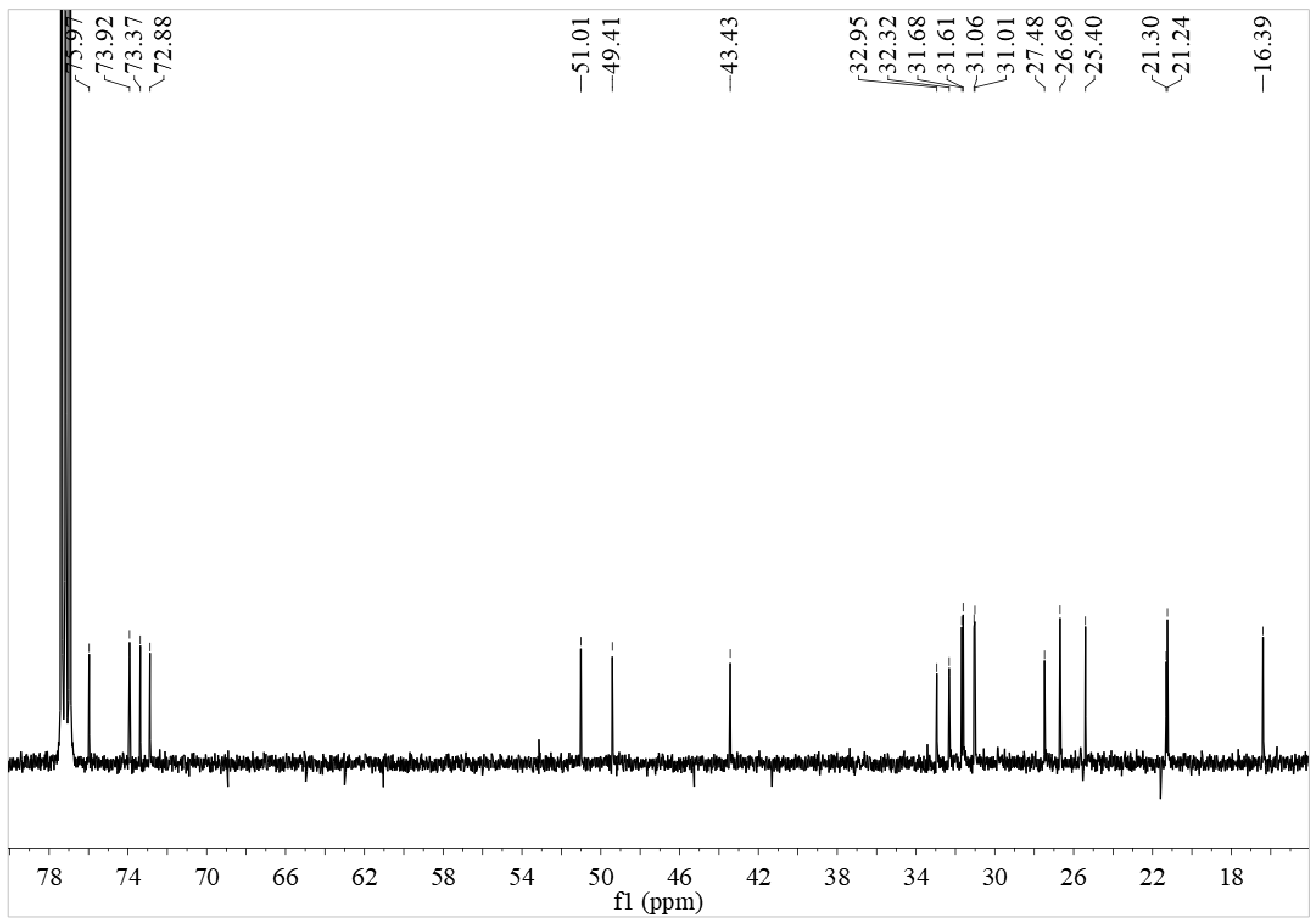


**Figure S46.** Partial ^13^C NMR (150 MHz, CDCl_3_) spectrum of compound **3**


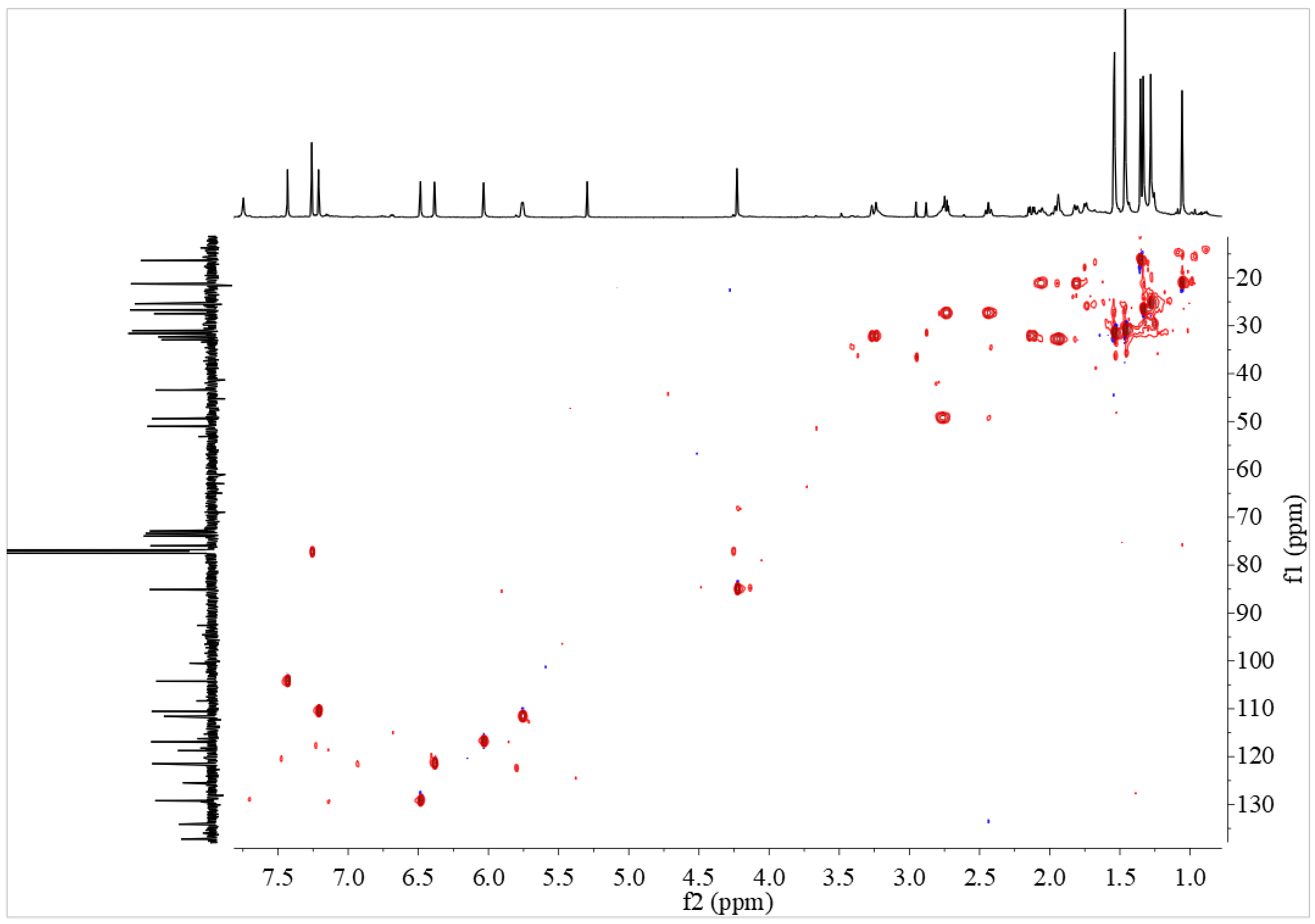


**Figure S47.** HSQC (CDCl_3_) spectrum of compound **3**


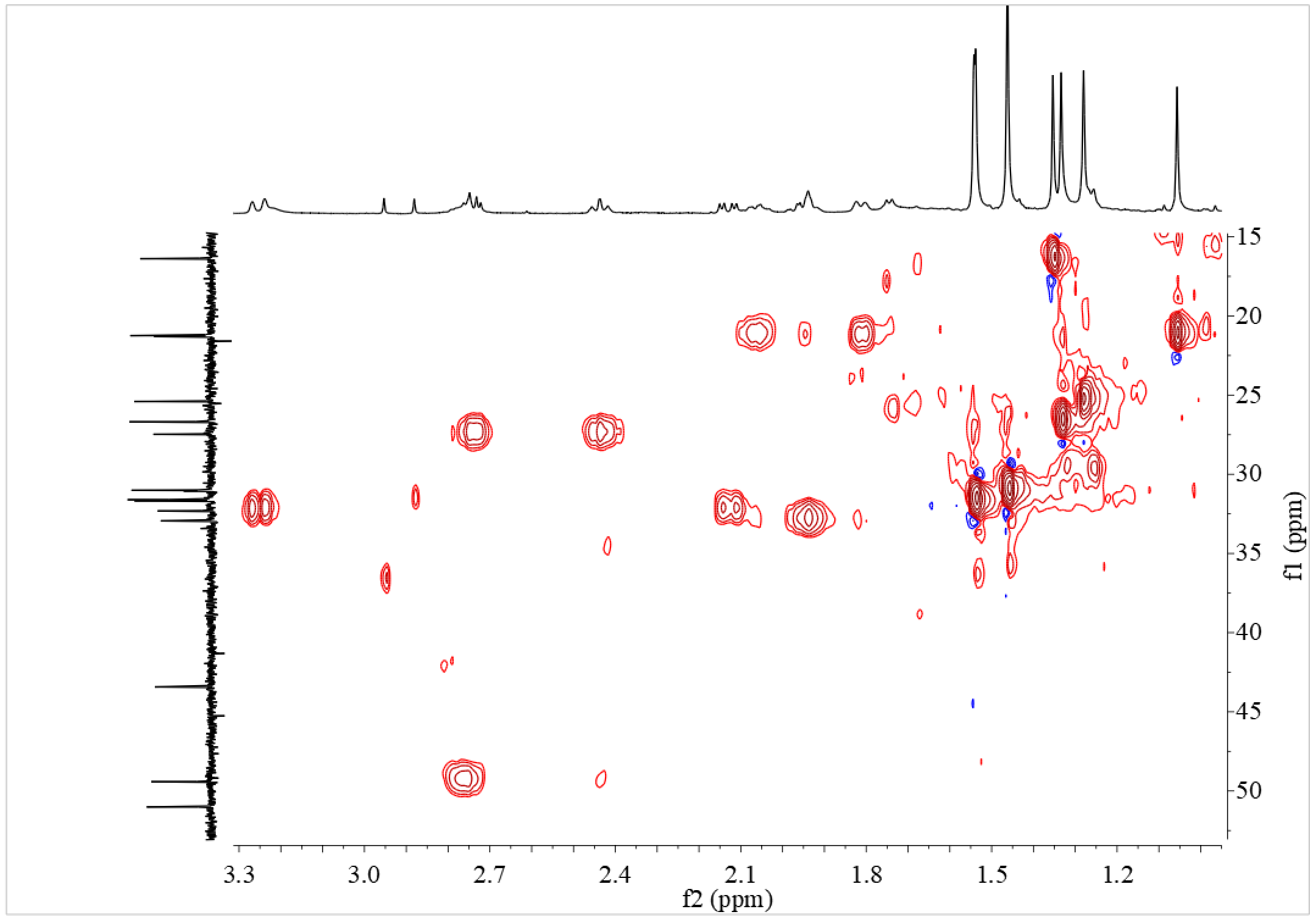


**Figure S48.** Partial HSQC (CDCl_3_) spectrum of compound **3**


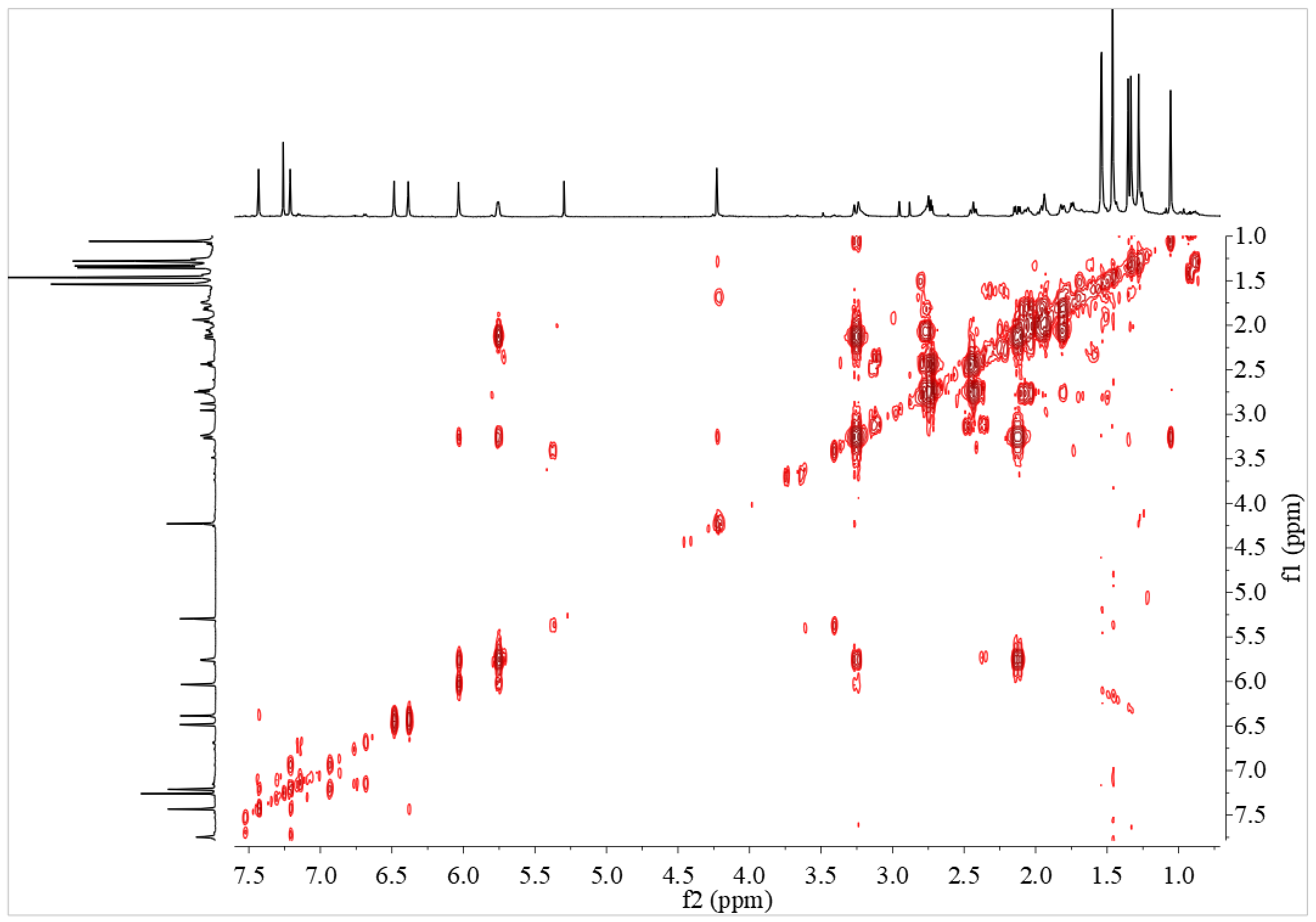


**Figure S49.** ^1^H-^1^H COSY (CDCl_3_) spectrum of compound **3**


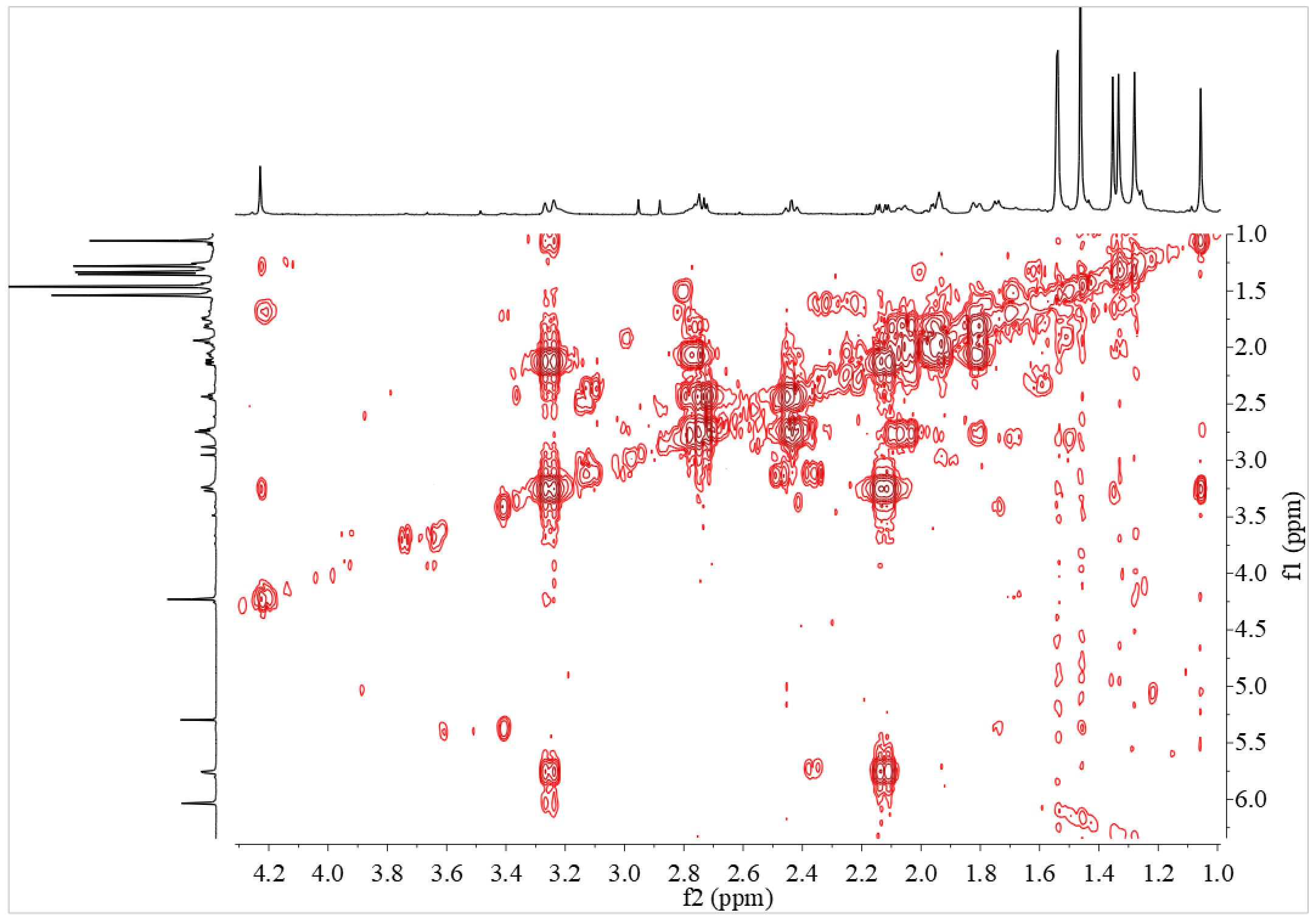


**Figure S50.** Partial ^1^H-^1^H COSY (CDCl_3_) spectrum of compound **3**


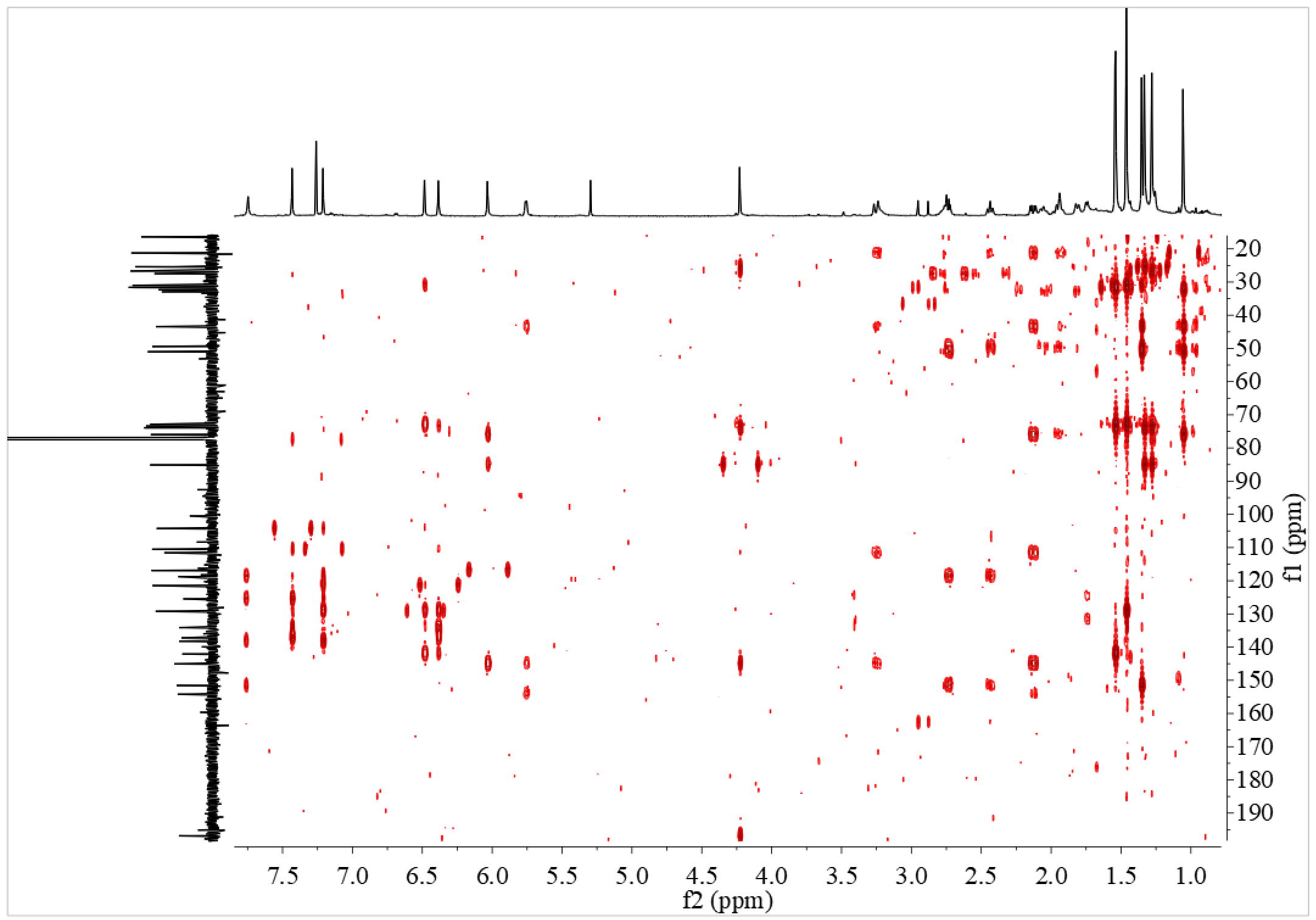


**Figure S51.** HMBC (CDCl_3_) spectrum of compound **3**


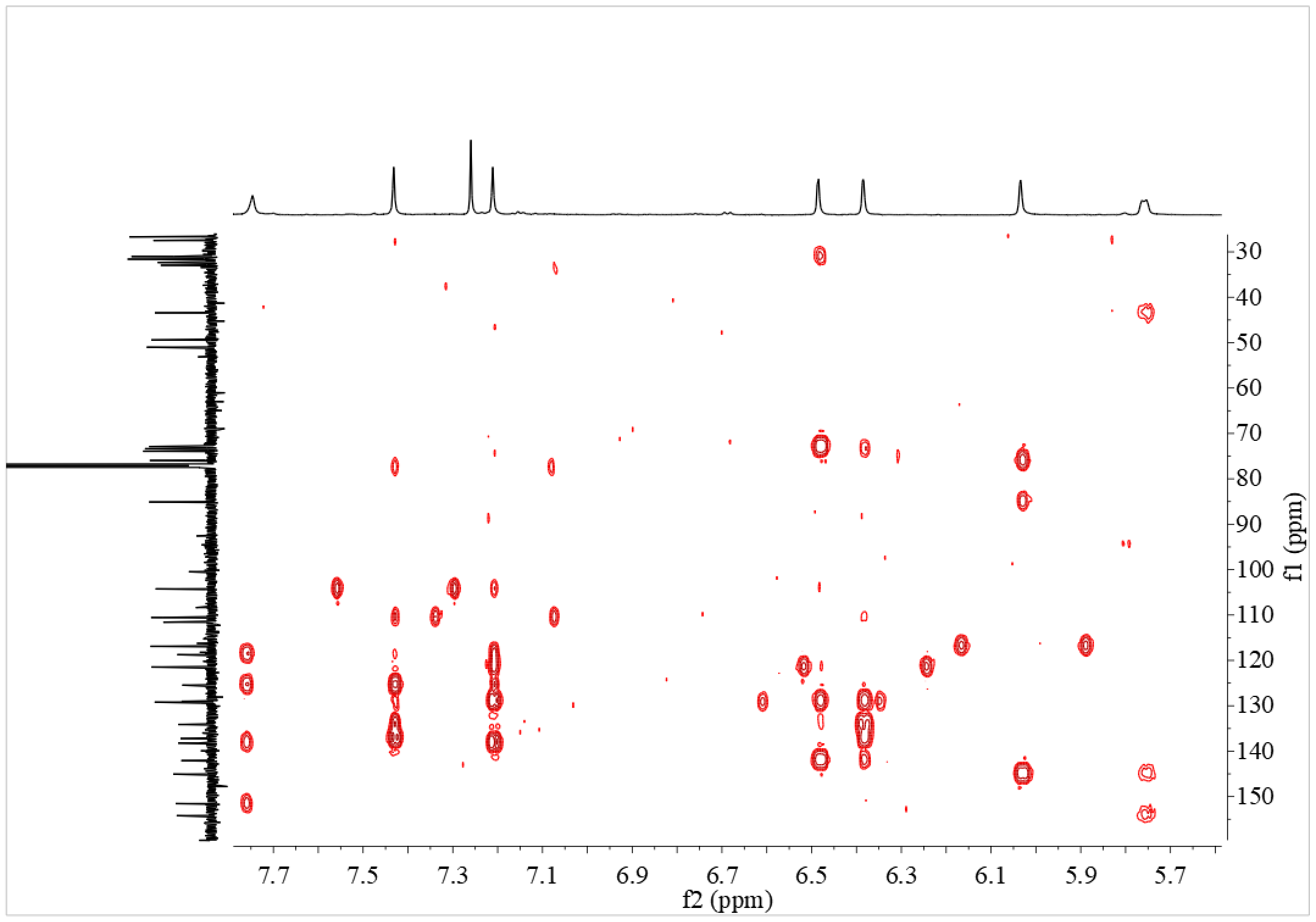


**Figure S52.** Partial HMBC (CDCl_3_) spectrum of compound **3**


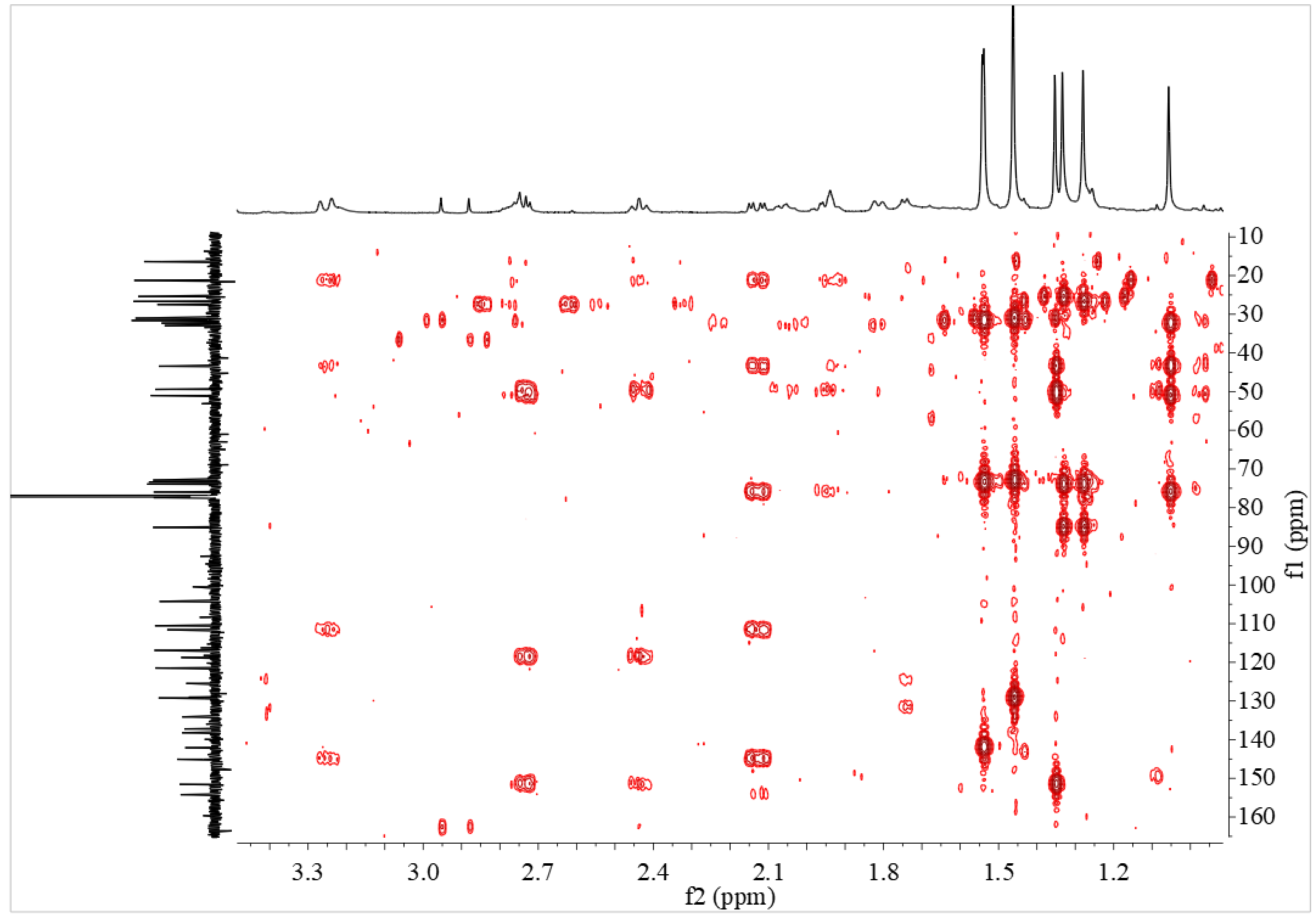


**Figure S53.** Partial HMBC (CDCl_3_) spectrum of compound **3**


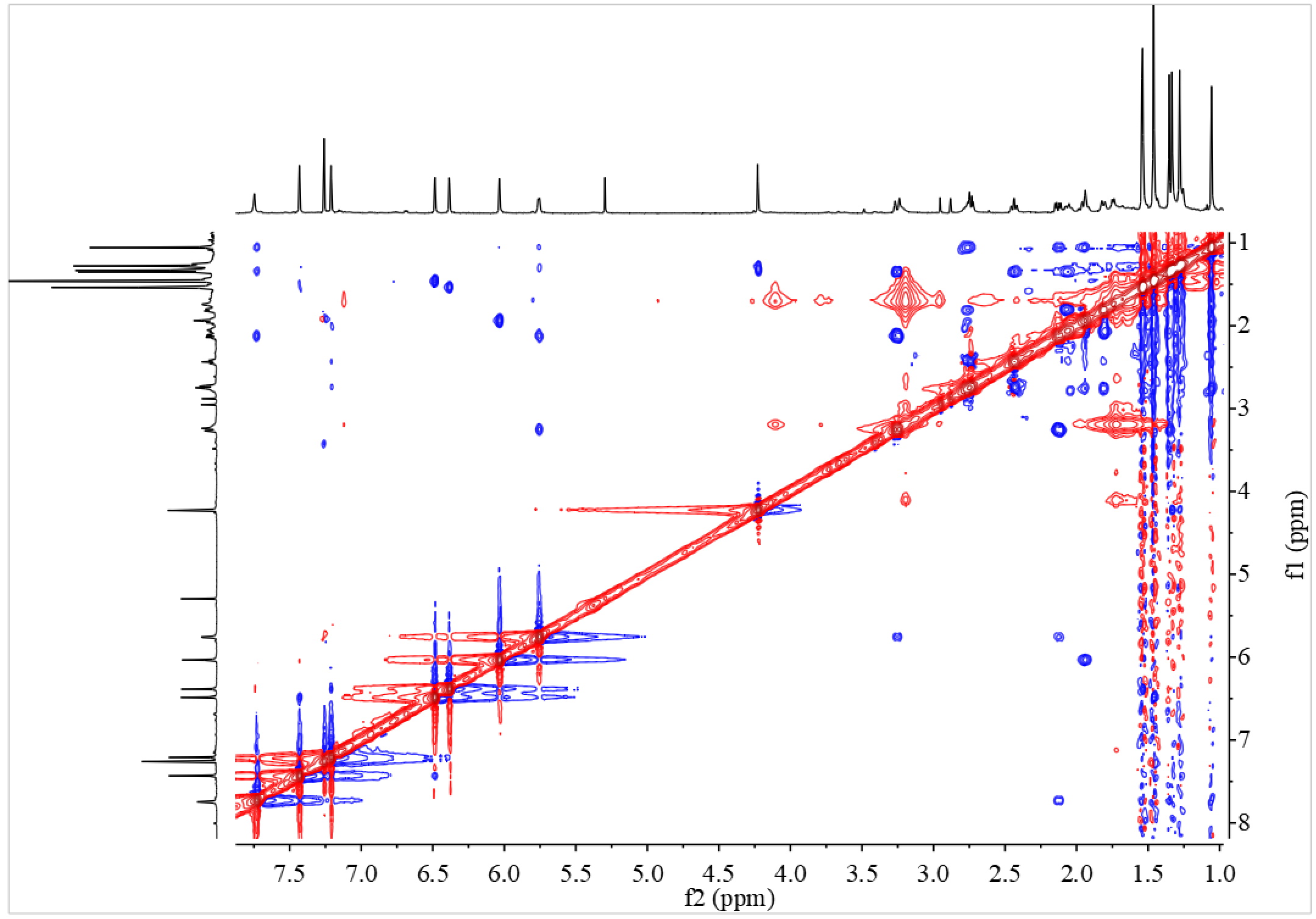


**Figure S54.** NOESY (CDCl_3_) spectrum of compound **3**


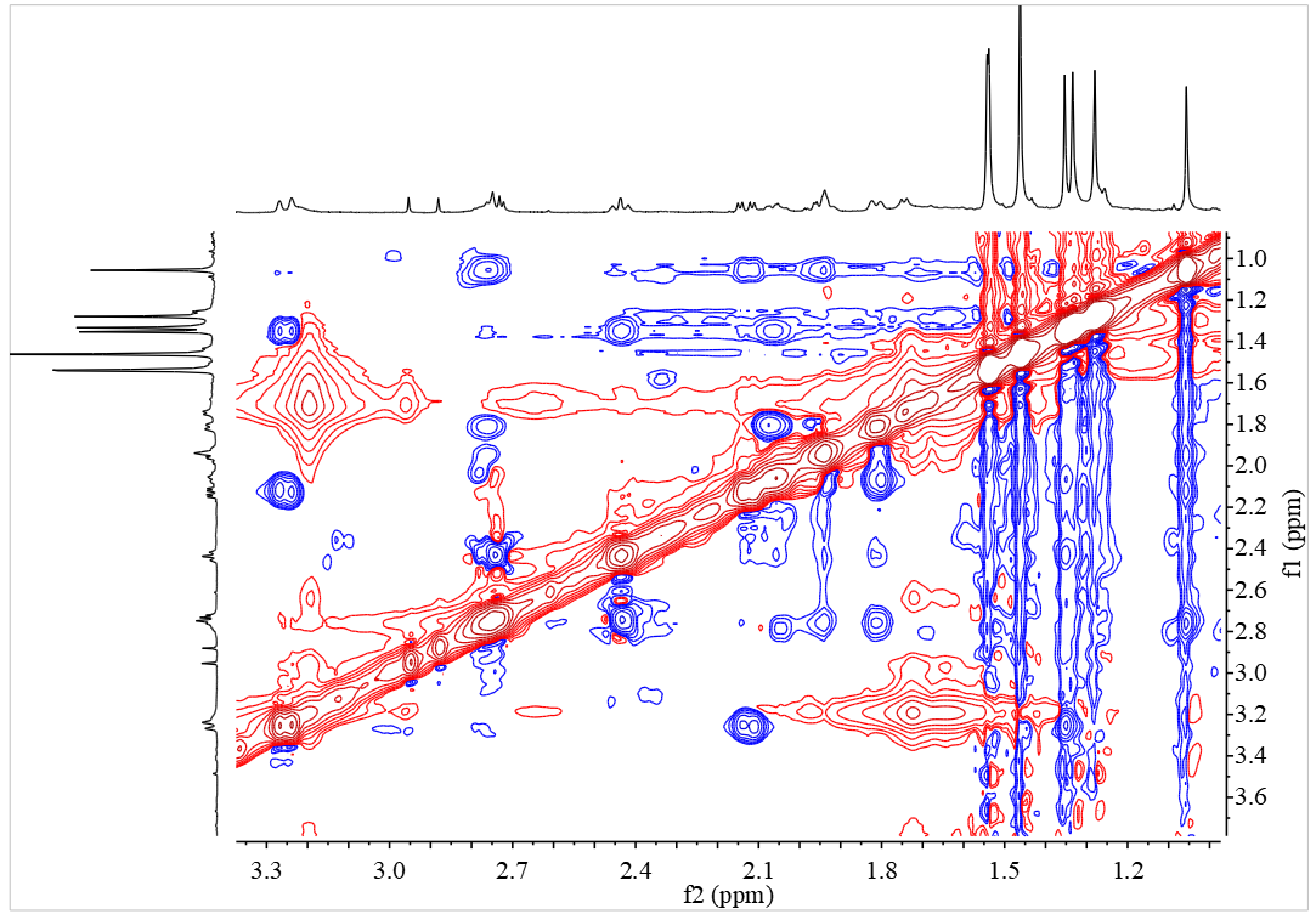


**Figure S55.** Partial NOESY (CDCl_3_) spectrum of compound **3**


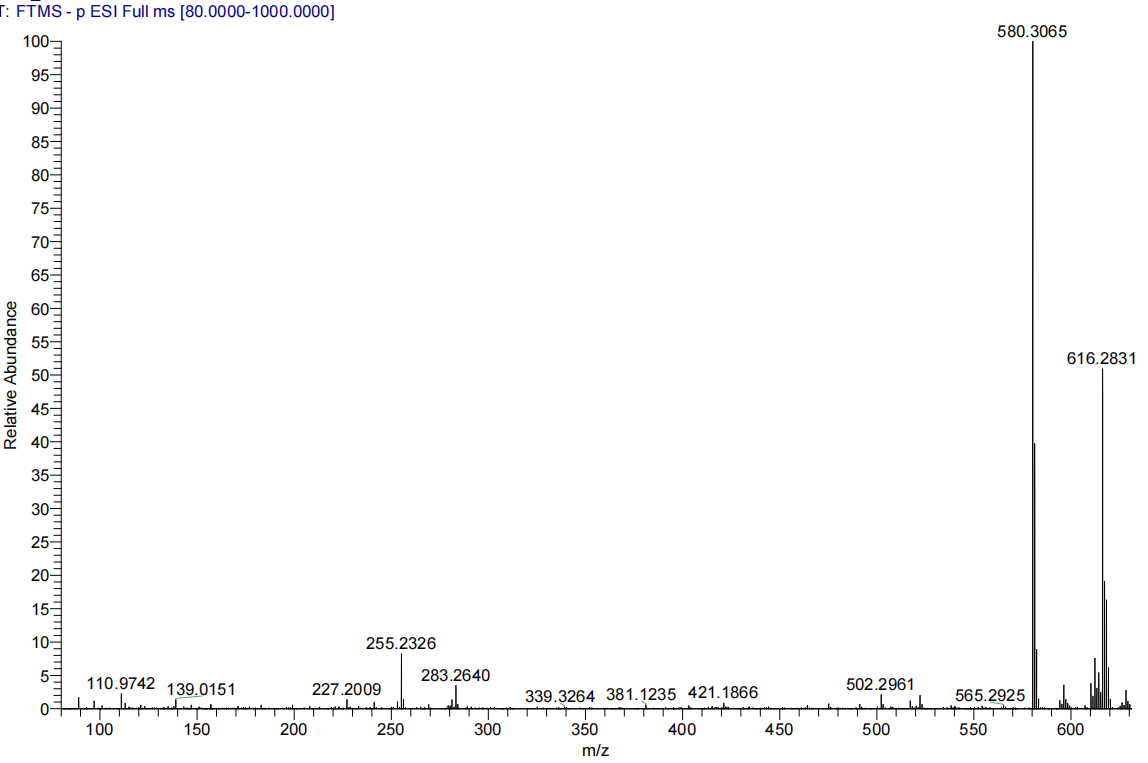


**Figure S56.** HRESIMS spectrum of compound **3**


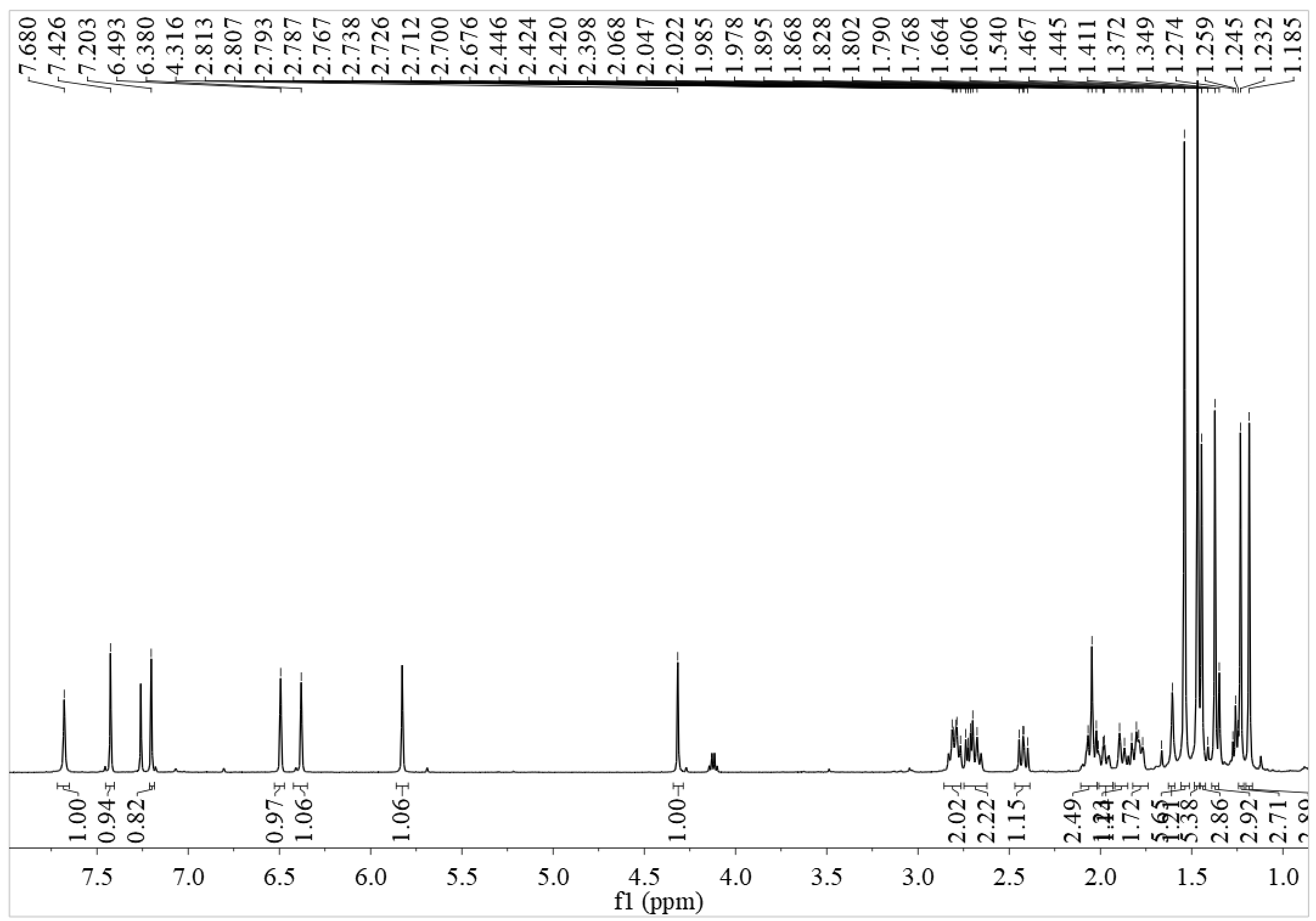


**Figure S57.** ^1^H NMR (600 MHz, CDCl_3_) spectrum of compound **4**


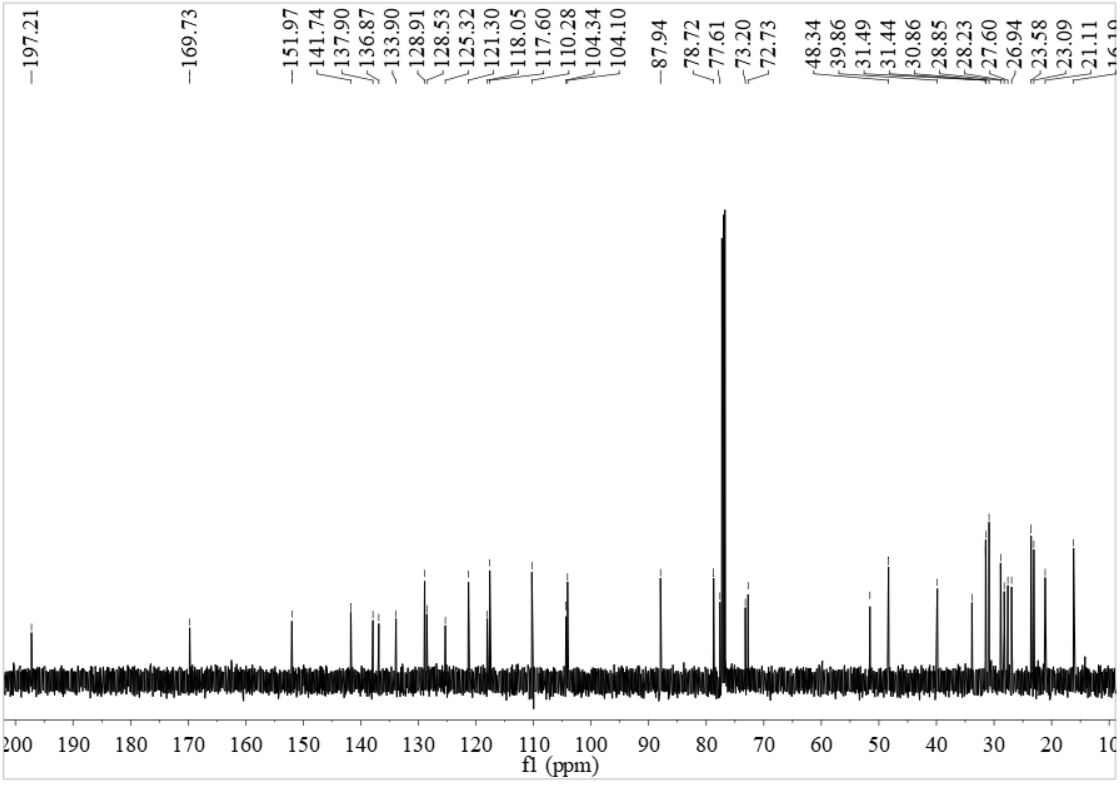


**Figure S58.** ^13^C NMR (150 MHz, CDCl_3_) spectrum of compound **4**


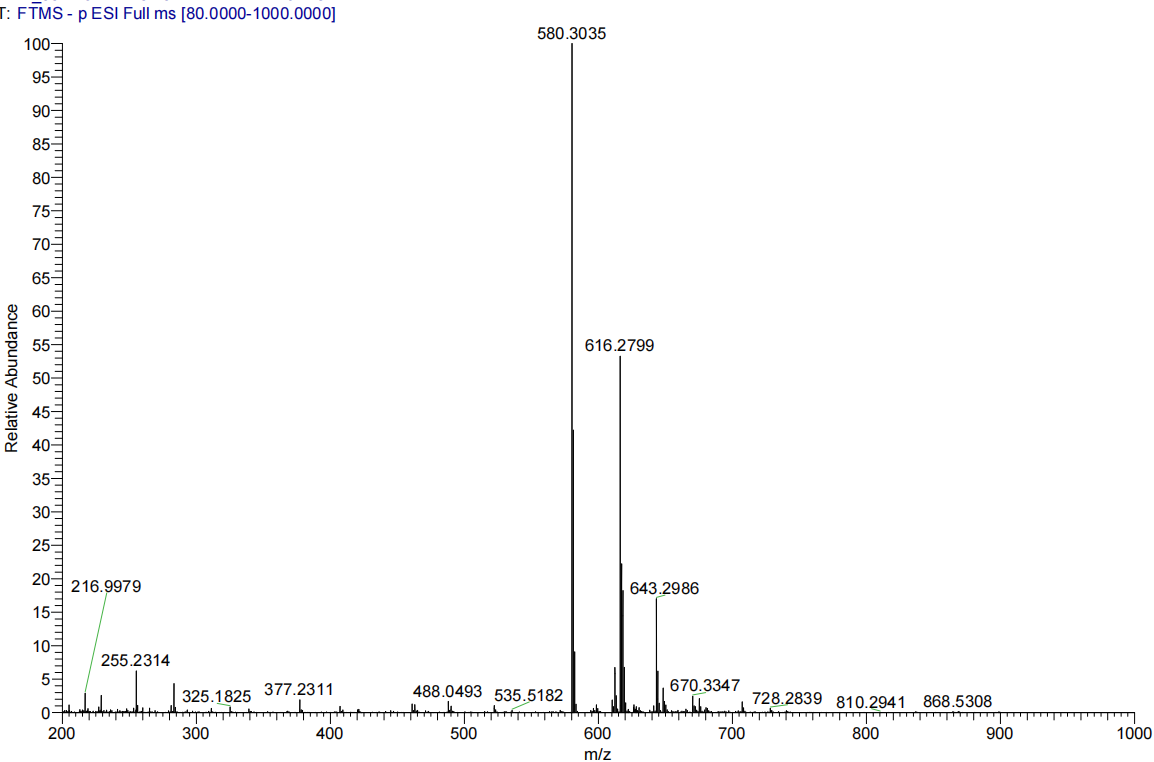


**Figure S59.** HRESIMS spectrum of compound **4**


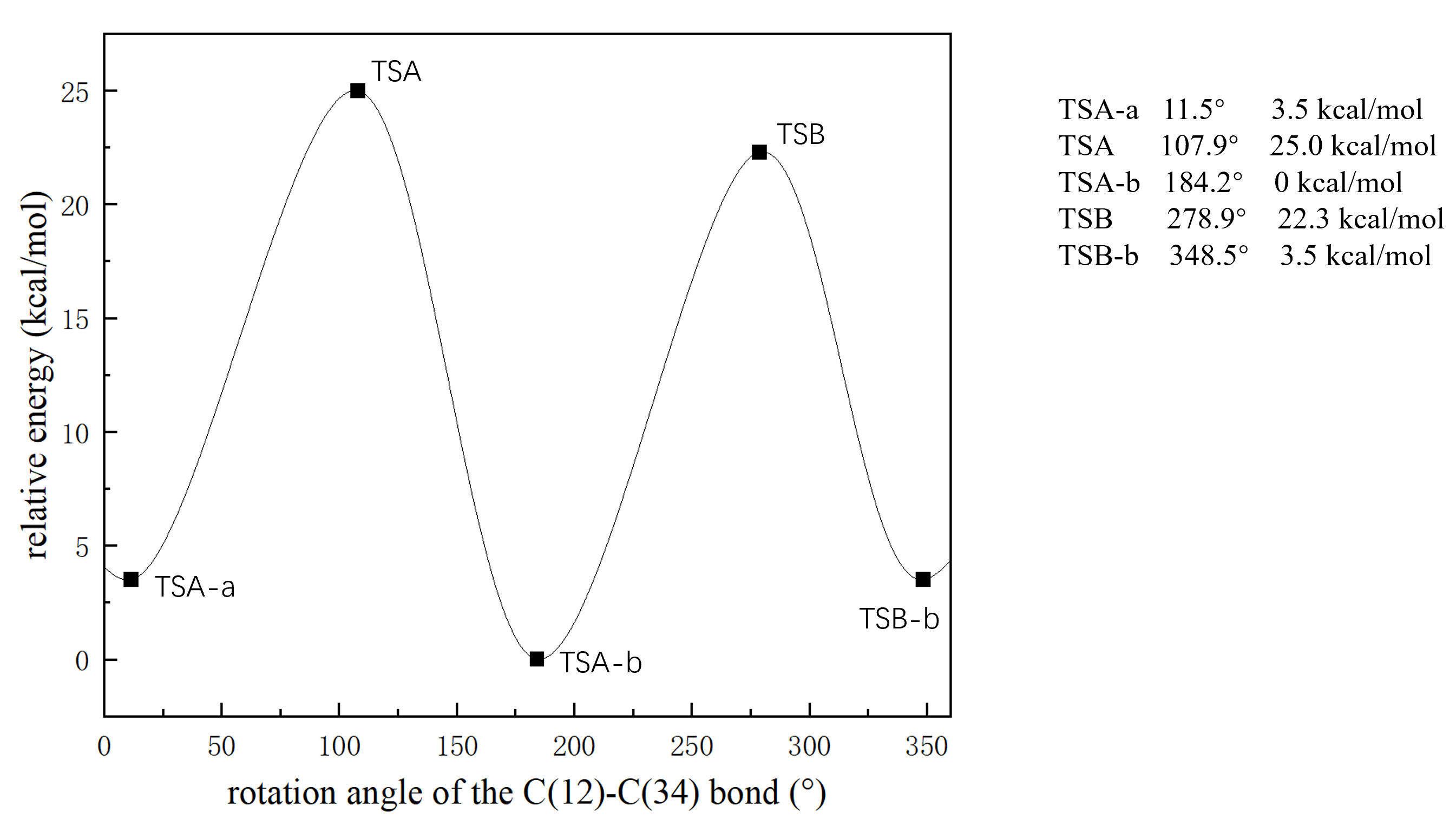


**Figure S60.** The ΔG of the *M* and *P* conformations of (3*S*,4*R*,7*S*,9*R*,13*S*,16*S*,3'*S*,4'*R*,9'*R*,13'*S*,16'*S*,22'*R*,23'*R*)-**1**

**Table S1** ^1^H and ^13^C NMR Data of **1** (600 MHz, CDCl_3_, *J* in Hz)

| no. | *δ*_H_ | *δ*_C_ | no. | *δ*_H_ | *δ*_C_ |
| --- | --- | --- | --- | --- | --- |
| 1-NH | 7.62, s | - | 1'-NH | 7.66, s | - |
| 2 | - | 153.0, C | 2' | - | 150.7, C |
| 3 | - | 51.7, C | 3' | - | 50.2, C |
| 4 | - | 40.0, C | 4' | - | 43.2, C |
| 5 | 1.83, m | 27.1, CH_2_ | 5' | 2.07, dd (13.2, 6.6) | 32.2, CH_2_ |
|  | 2.67, m |  |  | 3.14, d (13.2) |  |
| 6 | 2.05, m  2.79, dt (10.8, 3.6) | 28.4, CH_2_ | 6' | 5.72, d (6.6) | 111.6, CH |
|  |  |  |  |  |  |
| 7 | - | 104.5, C | 7' | - | 145.0, C |
| 9 | 4.31, s | 88.1, CH | 9' | 4.20, s | 85.0, CH |
| 10 | - | 197.3, C | 10' | - | 197.0, C |
| 11 | 5.81, s | 117.8, CH | 11' | 5.97, s | 116.8, CH |
| 12 | - | 169.8, C | 12' | - | 154.2, C |
| 13 | - | 77.8, C | 13' | - | 75.9, C |
| 14 | 1.80, m | 32.7, CH_2_ | 14' | 1.96, m | 34.0, CH_2_ |
|  | 1.85, m |  |  | 2.32, m |  |
| 15 | 1.70, m | 21.2, CH_2_ | 15' | 1.68, m | 20.9, CH_2_ |
|  | 1.96, d (6.6) |  |  | 1.60, m |  |
| 16 | 2.69, m | 48.3, CH | 16' | 2.46, m | 48.5, CH |
| 17 | 0.63, dd (12.6, 10.8) | 27.8, CH_2_ | 17' | 1.29, m | 29.5, CH_2_ |
|  | 2.47, dd (12.6, 6.0) |  |  | 1.93, m |  |
| 18 | - | 117.4, C | 18' | - | 119.2, C |
| 19 | - | 126.8, C | 19' | - | 123.8, C |
| 20 | - | 128.2, C | 20' | 6.72, s | 114.7, CH |
| 21 | - | 141.4, C | 21' | - | 136.6, C |
| 22 | 6.60, s | 119.7, CH | 22' | 4.60, d (7.8) | 47.7, CH |
| 23 | - | 142.0, C | 23' | 3.37, dd (7.8, 3.0) | 57.4, CH |
| 24 | - | 73.4, C | 24' | - | 75.1, C |
| 26 | - | 73.0, C | 26' | - | 72.6, C |
| 27 | 6.53, s | 129.1, CH | 27' | 6.10, s | 120.7, CH |
| 28 | - | 138.7, C | 28' | - | 134.4, C |
| 29 | - | 128.3, C | 29' | - | 133.4, C |
| 30 | 7.40, s | 102.5, CH | 30' | 7.41, s | 103.3, CH |
| 31 | - | 140.4, C | 31' | - | 139.4, C |
| 32 | 1.33, s | 16.2, CH_3_ | 32' | 0.93, s | 15.9, CH_3_ |
| 33 | 1.19, s | 23.6, CH_3_ | 33' | 1.04, s | 21.3, CH_3_ |
| 34 | - | 78.9, C | 34' | - | 73.8, C |
| 35 | 1.44, s | 29.0, CH_3_ | 35' | 1.26, s | 25.3, CH_3_ |
| 36 | 1.17, s | 23.2, CH_3_ | 36' | 1.32, s | 26.7, CH_3_ |
| 37 | 1.56, s | 31.7, CH_3_ | 37' | 1.01, s | 29.9, CH_3_ |
| 38 | 1.58, s | 31.8, CH_3_ | 38' | 1.34, s | 23.7, CH_3_ |
| 39 | 1.50, s | 31.0, CH_3_ | 39' | 1.42, s | 30.1, CH_3_ |
| 40 | 1.50, s | 31.0, CH_3_ | 40' | 1.39, s | 32.5, CH_3_ |
| 13-OH | 3.23, brs | - | 13'-OH | 2.69, brs | - |

**Table S2** ^1^H and ^13^C NMR Data of **2** (600 MHz, CDCl_3_, *J* in Hz)

| no. | *δ*_H_ | *δ*_C_ | no. | *δ*_H_ | *δ*_C_ |
| --- | --- | --- | --- | --- | --- |
| 1-NH | 7.73, s | - | 1'-NH | 7.61, s | - |
| 2 | - | 147.3, C | 2' | - | 153.0, C |
| 3 | - | 48.0, C | 3' | - | 51.8, C |
| 4 | - | 42.8, C | 4' | - | 40.0, C |
| 5 | 1.62, m | 29.9, CH2 | 5' | 1.81, m | 27.1, CH2 |
|  | 2.38, td (13.8, 3.6) |  |  | 2.67, m |  |
| 6 | 2.03, m | 34.6, CH2 | 6' | 2.05, m | 28.4, CH2 |
|  | 2.26, td (13.8, 3.0) |  |  | 2.79, dd (10.2, 13.2) |  |
| 7 | - | 94.4, C | 7' | - | 104.5, C |
| 9 | 4.27, s | 78.2, CH | 9' | 4.31, s | 88.1, CH |
| 10 | - | 199.8, C | 10' | - | 197.3, C |
| 11 | 5.94, s | 121.6, CH | 11' | 5.81, s | 117.8, CH |
| 12 | - | 160.6, C | 12' | - | 169.8, C |
| 13 | - | 140.7, C | 13' | - | 77.7, C |
| 14 | 5.99, m | 132.3, CH | 14' | 1.94, m | 33.9, CH2 |
|  | - |  |  | 1.85, m |  |
| 15 | 1.84, m | 27.9, CH2 | 15' | 1.69, m | 21.2, CH2 |
|  | 1.98, m |  |  | 1.96, m |  |
| 16 | 2.60, m | 43.9, CH | 16' | 2.69, m | 48.5, CH |
| 17 | 0.60, m | 29.3, CH2 | 17' | 2.14, dd (13.2, 11.4) | 27.8, CH2 |
|  | 2.07, m |  |  | 2.48, dd (13.2, 6.0) |  |
| 18 | - | 119.9, C | 18' | - | 117.4, C |
| 19 | - | 123.5, C | 19' | - | 126.8, C |
| 20 | - | 128.5, C | 20' | 6.75, s | 114.8, CH |
| 21 | - | 136.7, C | 21' | - | 141.4, C |
| 22 | 6.62, s | 119.7, CH | 22' | 4.61, d (7.8) | 47.7, CH |
| 23 | - | 142.0, C | 23' | 3.36, dd (7.8, 2.4) | 57.5, CH |
| 24 | - | 73.4, C | 24' | - | 75.1, C |
| 26 | - | 73.0, C | 26' | - | 72.6, C |
| 27 | 6.54, s | 129.2, CH | 27' | 6.11, s | 120.7, CH |
| 28 | - | 134.4, C | 28' | - | 138.7, C |
| 29 | - | 128.4, C | 29' | - | 133.5, C |
| 30 | 7.42, s | 103.3, CH | 30' | 7.40, s | 102.6, CH |
| 31 | - | 139.8, C | 31' | - | 140.4, C |
| 32 | 0.61, s | 14.6, CH3 | 32' | 1.32, s | 16.1, CH3 |
| 33 | 1.19, s | 23.3, CH3 | 33' | 1.20, s | 23.6, CH3 |
| 34 | - | 72.8, C | 34' | - | 78.9, C |
| 35 | 1.25, s | 24.5, CH3 | 35' | 1.44, s | 29.0, CH3 |
| 36 | 1.28, s | 26.7, CH3 | 36' | 1.17, s | 23.2, CH3 |
| 37 | 1.58, s | 31.8, CH3 | 37' | 1.02, s | 29.8, CH3 |
| 38 | 1.56, s | 31.7, CH3 | 38' | 1.34, s | 23.7, CH3 |
| 39 | 1.50, s | 31.0, CH3 | 39' | 1.42, s | 30.1, CH3 |
| 40 | 1.50, s | 31.0, CH3 | 40' | 1.37, s | 32.5, CH3 |
| 7-OH | - | - | 7'-OH | 4.18, brs | - |
| 13-OH | - | - | 13'-OH | 2.70, brs | - |

**Table S3** ^1^H and ^13^C NMR Data of **3** (600 MHz, CDCl_3_, *J* in Hz)

| no. | *δ*_H_ | *δ*_C_ |
| --- | --- | --- |
| 1-NH | 7.75, brs | - |
| 2 | - | 151.6, C |
| 3 | - | 51.0, C |
| 4 | - | 43.4, C |
| 5 | 1.94, m | 33.0, CH_2_ |
| 6 | 5.76, d (4.8) | 111.6, CH |
| 7 | - | 145.1, C |
| 9 | 4.23, s | 85.1, CH |
| 10 | - | 196.9, C |
| 11 | 6.03, s | 116.9, CH |
| 12 | - | 154.2, C |
| 13 | - | 76.0, C |
| 14 | 3.26, d (17.4) | 32.3, CH_2_ |
|  | 2.13, dd (17.4, 6.0) |  |
| 15 | 2.06, m | 21.3, CH_2_ |
|  | 1.81, m |  |
| 16 | 2.77, m | 49.4, CH |
| 17 | 2.74, m | 27.5, CH_2_ |
|  | 2.44, m |  |
| 18 | - | 118.7, C |
| 19 | - | 125.5, C |
| 20 | 7.21, s | 110.5, CH |
| 21 | - | 137.2, C |
| 22 | 6.39, s | 121.5, CH |
| 23 | - | 142.1, C |
| 24 | - | 73.4, C |
| 26 | - | 72.9, C |
| 27 | 6.49, s | 129.2, CH |
| 28 | - | 134.1, C |
| 29 | - | 129.0, C |
| 30 | 7.43, s | 104.3, CH |
| 31 | - | 138.2, C |
| 32 | 1.35, s | 16.4, CH_3_ |
| 33 | 1.06, s | 21.2, CH_3_ |
| 34 | - | 73.9, C |
| 35 | 1.33, s | 26.7, CH_3_ |
| 36 | 1.28, s | 25.4, CH_3_ |
| 37 | 1.54, s | 31.7, CH_3_ |
| 38 | 1.54, s | 31.6, CH_3_ |
| 39 | 1.46, s | 31.1, CH_3_ |
| 40 | 1.46, s | 31.0, CH_3_ |
| 13-OH | 3.22, brs | - |

**Table S4.** The coordinates for the lowest-energy conformer of **1a** in ECD calculation

-------------------------------------------------------------------------------------------

Coordinates (Angstroms)

X Y Z

-------------------------------------------------------------------------------------------

C 0.67673500 -3.30735100 0.57561900

C 1.10861500 -2.24851300 1.44348900

C 2.37584800 -1.67105200 1.24084300

C 3.14656500 -2.12649800 0.18494900

C 2.72231300 -3.20346500 -0.63387200

C 1.48293500 -3.81760400 -0.44304200

N -0.64239700 -3.61223000 0.89923700

C -1.05694800 -2.74083700 1.88862800

C -0.01379200 -1.94328900 2.28065500

C 4.47658800 -1.55276700 -0.30844900

C 4.63065200 -2.20163600 -1.72882800

C 3.70340700 -3.40733200 -1.70335300

C -2.30356900 -2.55730000 2.72937200

C -1.95524300 -1.12592200 3.28693700

C -0.41231600 -1.07708400 3.45118200

C -3.70237900 -2.46557500 2.00290100

C -4.78868100 -1.86065800 2.99335400

C -4.32565800 -0.58874600 3.73505000

C -2.92804400 -0.70270100 4.37987900

C -4.22772700 -3.85118600 1.54270100

C -5.66941100 -3.81888500 1.10565300

C -6.50016000 -2.79094300 1.37555200

C -6.09269000 -1.65727300 2.21121300

O -7.79630000 -2.84984100 0.93991700

C -8.46613400 -1.62538200 0.58449500

C -8.16235800 -0.49796900 1.57172300

C -6.91723700 -0.59435400 2.33637700

C 6.03429200 -2.62199300 -2.22559500

O 5.85213100 -3.32188800 -3.47935900

C 4.96063300 -4.45015200 -3.58736200

C 3.87603400 -4.44100400 -2.53555000

C 4.54869300 -0.03372600 -0.22872900

C 5.56689200 0.59446000 0.49159500

C 5.59975300 2.01232800 0.70447100

C 4.59536600 2.83759800 0.23820800

C 3.57400100 2.22186200 -0.49294800

C 3.53557500 0.81512200 -0.77325600

C 6.74712900 0.03394300 1.17402900

C 7.46080100 1.02593100 1.76230800

C 6.79333600 2.30831000 1.51275400

N 2.43550600 2.81007200 -1.02752500

C 1.67042800 1.82283300 -1.60472300

C 2.31769500 0.61382500 -1.51788500

C 0.40519900 1.78703300 -2.43634400

C 0.15931100 0.23980800 -2.33264700

C 1.55473600 -0.42644800 -2.31146000

C -0.88436900 2.52439400 -1.88512100

C -2.16786100 1.98498600 -2.63414600

C -2.25874600 0.44540400 -2.74183700

C -0.96467200 -0.21930700 -3.25078200

C 8.78305000 1.00496400 2.48506100

O 8.92106600 2.18689200 3.31162800

C 8.50118400 3.49426300 2.88738600

C 7.28334400 3.46144900 1.99592700

C -0.80733100 4.08011800 -2.05631600

C -2.15090400 4.81301900 -2.31385200

C -3.35662200 4.12683800 -1.70119700

C -3.41129500 2.61490200 -2.00650800

O -4.56837900 4.70328800 -2.16938500

C -5.51056800 4.25394100 -1.19017200

C -5.74260800 2.75026800 -1.32848900

C -4.59113600 1.98525400 -1.82027600

C 5.77555600 -5.75928800 -3.52694700

C 4.33080400 -4.30509300 -4.98687200

C 6.88275600 -1.40934500 -2.61832300

C 6.80301500 -3.49491200 -1.21786800

C -2.24334500 -3.64040800 3.84507800

C -3.59212100 -1.57428800 0.73384400

O -8.92054100 0.45901300 1.67623800

C 8.86748400 -0.14789500 3.49361500

C 9.94850100 0.90785900 1.47735300

C 8.14575800 4.21011400 4.20506800

C 9.66163100 4.24391500 2.19472900

C 0.81078100 2.20807900 -3.87806900

C -1.04297100 2.23455200 -0.36513000

O -3.38176800 4.34155300 -0.27469700

O -6.81031700 2.24300900 -0.99036300

C -4.75100400 4.58653700 0.13392000

C -4.90019600 6.07502000 0.46731400

C -5.08935600 3.70617700 1.33564600

O -5.11087100 -2.78578400 4.05701700

C -8.17082800 -1.20241900 -0.89856600

C -8.16391200 -2.44212900 -1.79919900

C -9.23081300 -0.19859700 -1.37526600

O -6.86061000 -0.63083700 -0.98557400

H -2.12753300 -0.43796200 2.45204700

H 4.24589600 -1.48553700 -2.46713100

H -0.19924100 0.06540500 -1.31276400

H 2.70681000 -0.83529700 1.85125900

H 1.13395800 -4.60626500 -1.10568400

H -1.22424400 -4.25558100 0.38582100

H 5.26865300 -1.95043500 0.33521200

H -0.04045600 -0.04631400 3.40617800

H -0.07139400 -1.49837200 4.40893500

H -4.31582800 0.25127900 3.03046300

H -5.07962500 -0.36414600 4.49610500

H -2.65003000 0.27436000 4.79550100

H -2.94306400 -1.41126400 5.21512600

H -3.62100000 -4.20819700 0.69703800

H -4.09116100 -4.60167400 2.33180700

H -6.05167900 -4.64566700 0.51250500

H -9.52940800 -1.86724700 0.67018500

H -6.69913600 0.24487200 2.98801500

H 3.23650800 -5.32166900 -2.48968200

H 4.58838100 3.90803200 0.43188000

H 7.01056500 -1.01745400 1.18308600

H 2.17794200 3.77731600 -0.90683600

H 1.50543500 -1.41482700 -1.85484300

H 1.97732600 -0.55512700 -3.32023300

H -3.10158700 0.19949400 -3.40213100

H -0.77607900 0.04329100 -4.29795000

H -1.08477800 -1.30922500 -3.21050200

H 6.81190700 4.41950300 1.78220100

H -0.34817900 4.48887700 -1.14773600

H -0.14564700 4.33875800 -2.88491900

H -2.33169500 4.87187000 -3.38809900

H -2.11563600 5.83237400 -1.91804400

H -6.44801900 4.79899300 -1.30676500

H -4.74954000 0.92619500 -2.00160900

H 5.13283900 -6.62578400 -3.72417900

H 6.56581700 -5.73665600 -4.28476700

H 6.23977600 -5.89810400 -2.54708700

H 3.75801700 -3.37489700 -5.04647200

H 5.11835100 -4.27798400 -5.74834700

H 3.66167600 -5.14483000 -5.20673600

H 7.07929100 -0.76940200 -1.75276900

H 6.36658200 -0.81576300 -3.37929700

H 7.83933000 -1.74144400 -3.03593200

H 6.19014800 -4.31434000 -0.83138600

H 7.68396100 -3.91973100 -1.70946200

H 7.15166900 -2.90175300 -0.36534600

H -3.10733100 -3.60567500 4.50845100

H -2.18657300 -4.64291500 3.40674300

H -1.33219900 -3.50863900 4.43760800

H -3.41126700 -0.52228700 0.96664000

H -2.75782300 -1.91771800 0.11297700

H -4.50169200 -1.61352100 0.13088500

H 8.05615100 -0.07133400 4.22261400

H 9.82244200 -0.10502300 4.02771700

H 8.79539200 -1.11568300 2.98637700

H 10.90284000 1.03408100 2.00036400

H 9.94245300 -0.07065100 0.98470300

H 9.87090600 1.67015500 0.69726000

H 9.01777900 4.21485700 4.86748300

H 7.32973400 3.68498600 4.70972100

H 7.84099300 5.24634500 4.02072500

H 9.38788600 5.28824300 2.00372100

H 10.54463200 4.23003600 2.84243500

H 9.92357700 3.78531900 1.23813700

H -0.03862900 2.24721800 -4.55932700

H 1.29049600 3.19248500 -3.87747900

H 1.55086800 1.50329500 -4.27063400

H -1.33119700 1.20322500 -0.14792500

H -0.09168500 2.41677200 0.14406400

H -1.79172800 2.88761600 0.08489700

H -4.72320700 6.68720800 -0.42138800

H -4.17003900 6.35440500 1.23347700

H -5.90384300 6.28909300 0.85216200

H -4.50328100 4.02802900 2.20246300

H -6.15180000 3.78526300 1.58854100

H -4.86053400 2.65527900 1.14065400

H -5.61592600 -3.52051300 3.67092300

H -9.09835200 -3.00629900 -1.70783700

H -7.33451700 -3.10340000 -1.53997000

H -8.04464100 -2.12668000 -2.84076000

H -8.98336200 0.12522600 -2.39195200

H -9.27420900 0.67966600 -0.72621500

H -10.22616200 -0.65636800 -1.39280600

H -6.92996200 0.34136300 -0.88357800

H -2.50827900 0.02247100 -1.76191300

O -2.13651300 2.52650200 -3.98060100

H -2.94507100 2.22357000 -4.42655800

-------------------------------------------------------------------------------------------

**Table S5.** The coordinates for the lowest-energy conformer of *ent*-**1a** in ECD calculation

-------------------------------------------------------------------------------------------

Coordinates (Angstroms)

X Y Z

-------------------------------------------------------------------------------------------

C -0.67872400 -3.34591600 0.50720000

C -1.10583200 -2.30708400 1.40155700

C -2.37035100 -1.71892100 1.21244200

C -3.14273400 -2.14476200 0.14544000

C -2.72396500 -3.20416300 -0.69878400

C -1.48767400 -3.82858600 -0.52263600

N 0.63990800 -3.66235800 0.82043900

C 1.05828900 -2.81861700 1.83087300

C 0.01845300 -2.02781000 2.24540800

C -4.46892000 -1.55234100 -0.33506400

C -4.62528300 -2.16585600 -1.77096100

C -3.70550700 -3.37734700 -1.77310700

C 2.30768900 -2.66073900 2.67253800

C 1.96403600 -1.24243300 3.26525500

C 0.42218700 -1.19533900 3.43896600

C 3.70342100 -2.55407100 1.94032800

C 4.79182900 -1.97096500 2.93077100

C 4.33540500 -0.70443300 3.69418300

C 2.94767400 -0.83982700 4.35662600

C 4.23083100 -3.92852300 1.44995500

C 5.64445200 -3.86391700 0.94435100

C 6.48671700 -2.86353500 1.26073400

C 6.10213300 -1.77465900 2.15784700

O 7.77961000 -2.90444300 0.80116900

C 8.45053700 -1.66842400 0.50315600

C 8.18846700 -0.60890000 1.57345700

C 6.95028600 -0.73613000 2.34524600

C -6.03117300 -2.56534200 -2.27931700

O -5.85174000 -3.23843400 -3.54831200

C -4.96747700 -4.36966200 -3.68092500

C -3.88346900 -4.39051800 -2.62895500

C -4.53590600 -0.03535000 -0.22166600

C -5.55822700 0.58007000 0.50396900

C -5.59829300 1.99513900 0.73270000

C -4.60148200 2.83164000 0.27065000

C -3.57043500 2.22751800 -0.45646300

C -3.52083200 0.82315600 -0.74755600

C -6.73867100 0.00702500 1.17569700

C -7.47126100 0.99192200 1.75269300

C -6.81305900 2.28071800 1.51246400

N -2.43170900 2.82727700 -0.97708400

C -1.65598200 1.84993100 -1.55669400

C -2.29519100 0.63564400 -1.48351600

C -0.38560100 1.82983000 -2.38092200

C -0.12690600 0.28444700 -2.28417000

C -1.51690100 -0.39371400 -2.27667400

C 0.89380600 2.57602100 -1.81890300

C 2.18641200 2.05288800 -2.56366700

C 2.29223900 0.51480300 -2.67776700

C 1.00724000 -0.15950500 -3.19749700

C -8.70389400 0.93667100 2.61822500

O -9.36653800 2.22503400 2.63149300

C -8.65561800 3.47266600 2.69387400

C -7.32921400 3.43092600 1.97457300

C 0.80202800 4.13181200 -1.98162700

C 2.13913000 4.87887100 -2.23118400

C 3.34838500 4.20212800 -1.61520800

C 3.42035600 2.69225200 -1.92646000

O 4.55676500 4.79323400 -2.07377300

C 5.49763800 4.34952300 -1.09082400

C 5.74556600 2.84853700 -1.23279400

C 4.60548000 2.07365400 -1.73631800

C -6.87107400 -1.33871400 -2.64634400

C 2.24382400 -3.77538200 3.75604500

C 3.58045200 -1.63040000 0.69603500

O 8.96407700 0.32685900 1.73268500

C -8.33910600 0.50003800 4.05332700

C -9.76800800 -0.00568000 2.04054300

C -8.46543600 3.92150900 4.16038500

C -9.59106800 4.46333400 1.97400400

C -0.78694200 2.25491000 -3.82276600

C 1.04782000 2.27873600 -0.29987500

O 3.36308700 4.41131700 -0.18732100

O 6.81565500 2.35127000 -0.88733600

C 4.72688600 4.67028400 0.22967900

O 5.06958500 -3.00561300 3.90534100

C 8.12094800 -1.13706500 -0.93754400

C 9.19434700 -0.13124800 -1.37815700

C 8.04858000 -2.31194200 -1.91815500

O 6.82595100 -0.52348200 -0.94388700

H 0.22638200 0.10730100 -1.26283600

H 2.12954400 -0.53614000 2.44484000

H -4.23487700 -1.43422300 -2.49095800

C 4.85794200 6.15946000 0.56786800

C 5.06750100 3.79062500 1.43126800

C -5.79050500 -5.67466500 -3.64925000

C -4.33593700 -4.19791000 -5.07659600

C -6.80769900 -3.45474100 -1.29209900

H -5.26390300 -1.96211800 0.29723800

O 2.15722500 2.60020100 -3.90793000

H -2.69742000 -0.89625800 1.84257100

H -1.14194400 -4.60229400 -1.20430600

H 1.21820000 -4.29969900 0.29560300

H 0.05205800 -0.16300900 3.42461300

H 0.08449900 -1.64357900 4.38561600

H 5.09368100 -0.47264400 4.45327700

H 4.31593800 0.15118100 3.00795800

H 2.97893400 -1.57107500 5.17185000

H 2.67013300 0.12451200 4.80097500

H 4.17160800 -4.66912600 2.25540600

H 3.58448700 -4.29880000 0.64003300

H 6.00578800 -4.65766900 0.29570900

H 9.51325900 -1.92567300 0.53896300

H 6.74488200 0.07598200 3.03484900

H -3.24885400 -5.27556100 -2.60285500

H -4.60569100 3.90164000 0.46695900

H -6.97399500 -1.05052300 1.20984500

H -2.18323500 3.79610300 -0.85026600

H -1.46254000 -1.38377200 -1.82457000

H -1.93067900 -0.52144400 -3.28915500

H 3.14123900 0.27971800 -3.33403300

H 2.54008300 0.08946600 -1.69855600

H 0.82237700 0.10649900 -4.24449100

H 1.13742100 -1.24840200 -3.16169900

H -6.82440500 4.38493100 1.83024900

H 0.14015500 4.38862500 -2.81062000

H 0.33653300 4.53124800 -1.07210000

H 2.09213500 5.89647200 -1.83203500

H 2.32395100 4.94312200 -3.30441000

H 6.43032300 4.90421600 -1.20010200

H 4.77644700 1.01704700 -1.92067600

H -7.06714500 -0.71787100 -1.76696600

H -6.34908600 -0.73095700 -3.39203000

H -7.82819800 -1.65558000 -3.07432500

H 3.09900600 -3.75704200 4.43030500

H 1.32367200 -3.66803200 4.33972700

H 2.20384800 -4.76416000 3.28673200

H 2.75418000 -1.97414900 0.06530500

H 3.37817600 -0.58833900 0.95429800

H 4.49127500 -1.63610900 0.09492200

H -9.21375300 0.60199900 4.70507900

H -8.01332600 -0.54597900 4.05999400

H -7.52304300 1.10219300 4.46259800

H -10.66099000 0.00910900 2.67416400

H -10.04898400 0.31540400 1.03385700

H -9.39329400 -1.03339600 1.99094000

H -8.05248800 4.93620000 4.20304500

H -9.43372200 3.91949400 4.67214400

H -7.78448500 3.25863300 4.70000300

H -10.56538000 4.47829400 2.47366500

H -9.17577700 5.47726500 1.98645300

H -9.73843200 4.15343000 0.93550400

H 0.06611100 2.30591100 -4.49863900

H -1.51783000 1.54522600 -4.22357700

H -1.27620500 3.23460100 -3.81971500

H 0.09163500 2.44552200 0.20545300

H 1.34824500 1.24977500 -0.08789400

H 1.78600900 2.93827000 0.15809100

H 5.85858300 -2.72425700 4.39667800

H 10.17450800 -0.61366500 -1.46268200

H 8.92486900 0.27362000 -2.35960300

H 9.28508900 0.69545400 -0.66860700

H 7.20760700 -2.96470100 -1.67505100

H 8.96709800 -2.90836000 -1.89561800

H 7.90659000 -1.92370900 -2.93199800

H 6.92751400 0.44102700 -0.80555700

H 4.12008700 6.42906000 1.33018100

H 5.85685500 6.38349000 0.95935400

H 4.67977700 6.77190900 -0.32039800

H 4.85375100 2.73744100 1.23173300

H 6.12689800 3.88297200 1.69231700

H 4.47086200 4.10251400 2.29455200

H -6.25644000 -5.83111800 -2.67291000

H -5.15297900 -6.54076000 -3.86427400

H -6.57994300 -5.63099900 -4.40700600

H -3.75813300 -3.26974500 -5.11554100

H -3.67113900 -5.03631300 -5.31402400

H -5.12273700 -4.15011800 -5.83781300

H -6.20109100 -4.28602800 -0.92107700

H -7.15523400 -2.87685800 -0.42873300

H -7.68995700 -3.86343700 -1.79481800

H 2.97042400 2.30554600 -4.35097900

-------------------------------------------------------------------------------------------

**Table S6.** The coordinates for the lowest-energy conformer of **1b** in ECD calculation

-------------------------------------------------------------------------------------------

Coordinates (Angstroms)

X Y Z

-------------------------------------------------------------------------------------------

C 2.30917600 0.39063300 1.50048000

C 1.98346400 1.01271600 0.24848900

C 0.81061400 1.78692900 0.15519500

C 0.01857800 1.92140100 1.28314600

C 0.36194800 1.30333700 2.51267200

C 1.51676100 0.53146800 2.63941000

N 3.48786000 -0.32793900 1.32766400

C 3.92967700 -0.12986600 0.03397800

C 3.03978500 0.65596700 -0.65247100

C -1.23978800 2.77394200 1.44150100

C -1.83593700 2.27790300 2.80536000

C -0.66620200 1.61047200 3.51095200

C 4.97389600 -0.75922900 -0.86585200

C 4.91831900 0.32003400 -2.00881000

C 3.43380200 0.76882200 -2.10582500

C 6.46287100 -0.87666600 -0.36245400

C 7.42666300 -1.16976700 -1.58846500

C 7.20387400 -0.23416900 -2.79402000

C 5.72741600 -0.11066000 -3.22559600

C 6.65294300 -2.02896500 0.66049100

C 8.09814600 -2.36645600 0.91754700

C 9.11375400 -1.94582700 0.13252200

C 8.88107300 -1.18506800 -1.09532300

O 10.39500100 -2.14819000 0.56718100

C 11.49509000 -2.07956700 -0.35413200

C 11.30757100 -0.96578000 -1.38890700

C 9.93101000 -0.64909500 -1.76078300

C -2.51737900 3.29357300 3.75458200

O -2.82535900 2.58772100 4.98113600

C -1.81331600 1.86522200 5.71026800

C -0.65318100 1.44518300 4.83937600

C -2.19286800 2.77626700 0.26068400

C -2.52444100 3.96980700 -0.38427400

C -3.45679000 4.02471800 -1.47183700

C -4.07916300 2.88738400 -1.94820700

C -3.72911200 1.68197200 -1.33062000

C -2.80222000 1.58735800 -0.23945500

C -2.06648900 5.35004900 -0.14411900

C -2.67764300 6.20024100 -1.00616600

C -3.57645700 5.43455700 -1.87710100

N -4.17774100 0.40553400 -1.63716500

C -3.59817400 -0.47903700 -0.75894800

C -2.74645800 0.18499000 0.09152900

C -3.47252300 -1.98456400 -0.68934000

C -2.90372800 -2.05731700 0.77087600

C -1.98719500 -0.81682900 0.93962900

C -4.78109100 -2.86669400 -0.77057400

C -4.46690200 -4.32983500 -0.26319800

C -3.70219700 -4.37894900 1.08167700

C -2.45599900 -3.46954200 1.12498100

C -2.49437500 7.67768700 -1.24038700

O -3.64480200 8.22253200 -1.93173900

C -4.31781800 7.53343400 -2.99860900

C -4.31279400 6.03364800 -2.82690200

C -5.36569100 -2.92611400 -2.22340400

C -6.03937200 -4.25794600 -2.63722500

C -6.68467300 -5.00565000 -1.48727300

C -5.75341900 -5.15575200 -0.26453400

O -7.08544800 -6.30441100 -1.89832100

C -8.01575800 -6.66945500 -0.87117400

C -7.26499700 -6.94654000 0.43446700

C -6.06815200 -6.10626500 0.63806900

C -2.54510500 0.61905900 6.24742200

C -1.32324600 2.72083800 6.89753900

C -1.66034700 4.53941600 4.03717300

C -3.89561900 3.71666100 3.23856800

C 4.36732100 -2.11610400 -1.33272900

C 6.89816000 0.43856800 0.34016700

O 12.26840700 -0.44613600 -1.95578400

C -2.46294000 8.46715400 0.07465300

C -1.20208300 7.94243300 -2.04266400

C -5.76697100 8.04993200 -2.91192400

C -3.71618200 7.92486200 -4.36742800

C -5.88371000 -2.25755800 0.14189700

O -7.91004900 -4.35837300 -1.08970700

O -7.65706300 -7.77565800 1.24112500

C -8.89237900 -5.38134600 -0.78320700

C -9.94798400 -5.37978400 -1.89390900

C -9.51235000 -5.09684300 0.58356400

O 7.22263200 -2.49267200 -2.13655900

C 12.80803500 -1.96675400 0.47487000

C 12.86813000 -0.64545700 1.25775900

C 12.93193700 -3.16503300 1.41869100

O 13.89535900 -2.07384600 -0.43781900

H 5.42313700 1.20512500 -1.60340600

H -2.59470000 1.51771700 2.58175600

H -3.75019100 -1.83403400 1.43016500

H 0.53273300 2.26589000 -0.78006900

H 1.77449000 0.04332800 3.57650800

H 3.98123100 -0.80588300 2.06520800

H -0.89875200 3.80502300 1.58532800

H 3.35012100 1.77937500 -2.52357600

H 2.83444100 0.10790800 -2.75007400

H 7.58274700 0.76518000 -2.54955300

H 7.81037100 -0.61707500 -3.62085000

H 5.65611600 0.63596900 -4.02668900

H 5.36735600 -1.05811900 -3.64154200

H 6.19122500 -1.74485400 1.61786300

H 6.11303200 -2.92920600 0.34064800

H 8.34931100 -2.91974600 1.81895600

H 11.55436200 -3.02442000 -0.91865700

H 9.81089600 -0.02527800 -2.63970700

H 0.20300200 1.00699500 5.35077400

H -4.79301000 2.91729200 -2.76846200

H -1.32848900 5.63305100 0.59796200

H -4.87734400 0.19061400 -2.33065700

H -1.88308100 -0.54750800 1.99483800

H -0.96947300 -0.98119700 0.55877500

H -4.37702900 -4.09552100 1.89816600

H -1.68741000 -3.83754600 0.43585200

H -2.02515700 -3.50535700 2.13362200

H -4.95077700 5.46538100 -3.50199100

H -6.09867500 -2.11492600 -2.31700600

H -4.58358600 -2.72704300 -2.95846100

H -5.29105100 -4.92567800 -3.06694100

H -6.81187100 -4.08157100 -3.39180200

H -8.57072200 -7.55528900 -1.18319700

H -5.48172600 -6.30610500 1.52975900

H -2.90389700 0.00511800 5.41595100

H -1.88090300 0.01080700 6.87211900

H -3.40735200 0.92526600 6.85025300

H -0.63964600 2.14607300 7.53405600

H -0.79643900 3.61559000 6.55572100

H -2.17921300 3.03347700 7.50495800

H -0.63228800 4.28019600 4.30658200

H -2.10648500 5.10114300 4.86391900

H -1.62767100 5.20078400 3.16439100

H -4.38559900 4.36435100 3.97348700

H -3.81330200 4.25823300 2.29161300

H -4.52883100 2.83771300 3.08298400

H 3.42850300 -1.93663700 -1.86520500

H 5.04131400 -2.67193000 -1.98401100

H 4.11940200 -2.74522200 -0.47088200

H 7.82759500 0.30139300 0.89793500

H 7.05849500 1.26558100 -0.35481600

H 6.12728500 0.75659600 1.04884100

H -2.37417900 9.53779300 -0.13731400

H -1.61181900 8.16306600 0.69290700

H -3.38509500 8.29816200 0.63726400

H -1.15038600 8.99967500 -2.32526300

H -0.32341800 7.69616800 -1.43631300

H -1.15833100 7.33280100 -2.94951200

H -5.77467800 9.14074800 -3.00803500

H -6.20350400 7.78355700 -1.94514500

H -6.38506500 7.62404900 -3.71015600

H -3.69789900 9.01596100 -4.46017500

H -2.69684600 7.54822400 -4.48311500

H -4.32046200 7.51713300 -5.18635300

H -5.68087400 -2.37662500 1.20878600

H -5.96824500 -1.18371200 -0.05296000

H -6.85539900 -2.71141900 -0.05881100

H -9.49706500 -5.65674900 -2.85113800

H -10.38060300 -4.37872900 -1.98988000

H -10.75586300 -6.08464100 -1.66564800

H -10.17723700 -5.91307800 0.88553100

H -8.74549000 -4.97458200 1.35242300

H -10.09913800 -4.17366300 0.53785500

H 7.48676500 -3.14253600 -1.46449200

H 12.01941800 -0.55109500 1.94339600

H 12.86564600 0.21510100 0.58078400

H 13.79444000 -0.61324800 1.83963400

H 13.89999100 -3.11790000 1.92720100

H 12.89305300 -4.10197700 0.85273600

H 12.13540900 -3.16805900 2.16684600

H 13.76035100 -1.37088700 -1.10267700

O -3.63945700 -4.97008700 -1.27112900

H -3.41395700 -5.42135600 1.27184200

H -3.53918000 -5.89876200 -1.00292300

C -2.38576900 -2.36473600 -1.74087800

H -1.43728200 -1.88642100 -1.47789100

H -2.23201700 -3.44001200 -1.81724300

H -2.65628600 -1.98874900 -2.73290900

-------------------------------------------------------------------------------------------

**Table S7.** The coordinates for the lowest-energy conformer of *ent*-**1b** in ECD calculation

-------------------------------------------------------------------------------------------

Coordinates (Angstroms)

X Y Z

-------------------------------------------------------------------------------------------

C -2.30917700 0.39062000 1.50045600

C -1.98346900 1.01271700 0.24847200

C -0.81062100 1.78693600 0.15518400

C -0.01858300 1.92139600 1.28313600

C -0.36195000 1.30331700 2.51265500

C -1.51676000 0.53144400 2.63938600

N -3.48786200 -0.32794800 1.32763600

C -3.92968200 -0.12986300 0.03395300

C -3.03979400 0.65598000 -0.65248900

C 1.23978100 2.77393900 1.44149800

C 1.83592700 2.27790000 2.80535900

C 0.66619800 1.61044700 3.51094000

C -4.97390700 -0.75921000 -0.86588000

C -4.91833600 0.32006900 -2.00882300

C -3.43381800 0.76885600 -2.10584000

C -6.46287800 -0.87665700 -0.36247400

C -7.42667600 -1.16973800 -1.58848300

C -7.20389600 -0.23412000 -2.79402500

C -5.72744100 -0.11060500 -3.22561000

C -6.65293600 -2.02897800 0.66044800

C -8.09813500 -2.36648900 0.91750300

C -9.11375100 -1.94584300 0.13249800

C -8.88108300 -1.18505400 -1.09533100

O -10.39499400 -2.14822300 0.56716300

C -11.49509700 -2.07956900 -0.35413100

C -11.30758500 -0.96576900 -1.38889400

C -9.93102800 -0.64907300 -1.76077300

C 2.51734500 3.29357400 3.75459500

O 2.82533800 2.58771300 4.98114000

C 1.81331100 1.86517900 5.71025800

C 0.65318000 1.44513900 4.83936100

C 2.19286800 2.77626600 0.26068500

C 2.52444900 3.96980800 -0.38426700

C 3.45680300 4.02472000 -1.47182400

C 4.07917600 2.88738600 -1.94819500

C 3.72911900 1.68197300 -1.33061500

C 2.80221900 1.58735600 -0.23945600

C 2.06649900 5.35005100 -0.14410900

C 2.67765900 6.20024300 -1.00615100

C 3.57647900 5.43456100 -1.87708100

N 4.17774700 0.40553500 -1.63716200

C 3.59817300 -0.47903900 -0.75895200

C 2.74645200 0.18498600 0.09152200

C 3.47252100 -1.98456600 -0.68935000

C 2.90371600 -2.05732400 0.77086200

C 1.98718100 -0.81683600 0.93961200

C 4.78109200 -2.86669400 -0.77057600

C 4.46690200 -4.32983500 -0.26320400

C 3.70218700 -4.37895200 1.08166600

C 2.45598600 -3.46954900 1.12496300

C 2.49439100 7.67768800 -1.24037700

O 3.64483600 8.22253500 -1.93169900

C 4.31786900 7.53344200 -2.99856100

C 4.31283200 6.03365500 -2.82686700

C 5.36570300 -2.92610900 -2.22340100

C 6.03937700 -4.25794400 -2.63722300

C 6.68467600 -5.00565000 -1.48727100

C 5.75341900 -5.15575100 -0.26453400

O 7.08544800 -6.30441300 -1.89831700

C 8.01575600 -6.66945800 -0.87116900

C 7.26499300 -6.94654000 0.43447100

C 6.06814800 -6.10626400 0.63807000

C 3.89557600 3.71670100 3.23858900

C -4.36734000 -2.11608200 -1.33277900

C -6.89816600 0.43856200 0.34018100

O -12.26842500 -0.44611500 -1.95575400

C 2.46291700 8.46715900 0.07465900

C 1.20211900 7.94242400 -2.04269000

C 5.76702500 8.04992900 -2.91184100

C 3.71626700 7.92489000 -4.36739000

C 2.38577500 -2.36473500 -1.74089700

C 5.88370100 -2.25755500 0.14190500

O 7.91005300 -4.35837600 -1.08970400

O 7.65705900 -7.77565600 1.24113300

C 8.89238000 -5.38135200 -0.78320200

O -7.22264400 -2.49263300 -2.13659900

C -12.80802700 -1.96675200 0.47489700

C -12.93192200 -3.16504000 1.41870800

C -12.86809000 -0.64546200 1.25780200

O -13.89537100 -2.07382000 -0.43776900

H 3.75017400 -1.83404000 1.43015900

H -5.42314800 1.20515600 -1.60340400

H 2.59470400 1.51772800 2.58175600

C 9.94798600 -5.37979200 -1.89390200

C 9.51234900 -5.09685000 0.58357100

C 1.32323100 2.72076200 6.89754800

C 2.54512300 0.61901700 6.24738400

C 1.66028600 4.53939300 4.03720500

H 0.89874300 3.80501900 1.58532500

O 3.63946400 -4.97008800 -1.27114100

H -0.53274300 2.26590800 -0.78007500

H -1.77448700 0.04329300 3.57647900

H -3.98122100 -0.80591700 2.06517200

H -3.35013700 1.77941400 -2.52357800

H -2.83446200 0.10794800 -2.75010100

H -7.81039800 -0.61701400 -3.62085700

H -7.58276800 0.76522500 -2.54954000

H -5.36738400 -1.05805700 -3.64157400

H -5.65614700 0.63603600 -4.02669100

H -6.11301700 -2.92920800 0.34058300

H -6.19121600 -1.74488300 1.61782400

H -8.34929000 -2.91980400 1.81889800

H -11.55439400 -3.02441300 -0.91866800

H -9.81092300 -0.02523900 -2.63968600

H -0.20299800 1.00693400 5.35075500

H 4.79302600 2.91729400 -2.76844600

H 1.32849500 5.63305100 0.59796800

H 4.87736200 0.19061800 -2.33064200

H 1.88305600 -0.54751900 1.99482000

H 0.96946200 -0.98120300 0.55874700

H 3.41394700 -5.42136000 1.27182800

H 4.37701300 -4.09552400 1.89816000

H 1.68740300 -3.83755500 0.43582900

H 2.02513900 -3.50536600 2.13360200

H 4.95082300 5.46539000 -3.50195100

H 4.58360600 -2.72702900 -2.95846300

H 6.09869400 -2.11492700 -2.31699300

H 6.81187700 -4.08157200 -3.39180100

H 5.29105300 -4.92567200 -3.06693900

H 8.57071800 -7.55529400 -1.18319000

H 5.48171800 -6.30610400 1.52975700

H 3.81324900 4.25829200 2.29164600

H 4.52880800 2.83776900 3.08298600

H 4.38554300 4.36438500 3.97352200

H -3.42851800 -1.93661200 -1.86524600

H -4.11943100 -2.74521700 -0.47094100

H -5.04133500 -2.67189100 -1.98407300

H -7.05853400 1.26558300 -0.35478500

H -7.82758400 0.30136500 0.89797100

H -6.12727600 0.75659000 1.04883800

H 2.37415000 9.53779600 -0.13731500

H 1.61178300 8.16306600 0.69289300

H 3.38506000 8.29817800 0.63729200

H 0.32343900 7.69616000 -1.43635900

H 1.15042500 8.99966400 -2.32529700

H 1.15839100 7.33278500 -2.94953400

H 6.20353400 7.78354200 -1.94505500

H 6.38513200 7.62404800 -3.71006300

H 5.77474200 9.14074500 -3.00794200

H 2.69692900 7.54826500 -4.48310300

H 3.69799700 9.01599100 -4.46012400

H 4.32056000 7.51716500 -5.18630600

H 2.65630900 -1.98876300 -2.73293000

H 2.23201000 -3.44000900 -1.81725300

H 1.43729100 -1.88640500 -1.47792700

H 6.85539300 -2.71141100 -0.05879800

H 5.96823300 -1.18370800 -0.05294800

H 5.68085900 -2.37662600 1.20879200

H -7.48676100 -3.14250800 -1.46453700

H -12.89305700 -4.10197800 0.85274200

H -12.13538100 -3.16808100 2.16684900

H -13.89996700 -3.11790200 1.92723500

H -12.01936200 -0.55111900 1.94342400

H -13.79438700 -0.61324800 1.83969700

H -12.86560800 0.21510200 0.58083700

H -13.76036800 -1.37085700 -1.10262300

H 10.38060600 -4.37873800 -1.98987600

H 10.75586500 -6.08464800 -1.66563600

H 9.49706800 -5.65676200 -2.85113000

H 10.17722900 -5.91309000 0.88554300

H 10.09914500 -4.17367500 0.53786200

H 8.74548700 -4.97458100 1.35242700

H 2.17919500 3.03340800 7.50496700

H 0.79640200 3.61551000 6.55575100

H 0.63964700 2.14597000 7.53406000

H 2.90392700 0.00510100 5.41590000

H 3.40736400 0.92522700 6.85022200

H 1.88093200 0.01074000 6.87206900

H 0.63223300 4.28014500 4.30661300

H 2.10641200 5.10111700 4.86395900

H 1.62759200 5.20077300 3.16443400

H 3.53919200 -5.89876500 -1.00293900

-------------------------------------------------------------------------------------------

**Table S8.** The coordinates for the lowest-energy conformer of **2a** in ECD calculation

-------------------------------------------------------------------------------------------

Coordinates (Angstroms)

X Y Z

-------------------------------------------------------------------------------------------

C 0.72633600 -2.73987900 1.43362400

C 0.19029700 -2.55530800 0.11465700

C -1.19570700 -2.35577600 -0.03766200

C -1.98524400 -2.32449500 1.09919100

C -1.43210900 -2.48176600 2.39654300

C -0.06543200 -2.69199800 2.58113900

N 2.09775700 -2.93838300 1.31316500

C 2.43561000 -2.83868000 -0.02187900

C 1.31031400 -2.62117200 -0.77640800

C -3.50781300 -2.21438600 1.17765600

C -3.76083000 -1.89050700 2.69011000

C -2.50148200 -2.37291000 3.39298300

C 3.65370000 -3.15801500 -0.86826900

C 3.17654700 -2.40608900 -2.16691600

C 1.64197200 -2.63415900 -2.25041400

C 5.06496700 -2.60144800 -0.43627700

C 6.07169000 -2.67145900 -1.67603500

C 5.49203600 -2.03189600 -2.95387700

C 4.12019500 -2.62587400 -3.34292900

C 5.70330600 -3.43727000 0.70270000

C 7.06362300 -2.88571700 1.15202000

C 8.06031400 -2.70617000 -0.00488600

C 7.41466000 -2.07318700 -1.24375800

O 9.11078500 -1.93819300 0.52119800

C 10.10986300 -1.48772600 -0.40446400

C 9.45181000 -0.71496500 -1.54682800

C 8.07534100 -1.10075500 -1.89447700

C -5.01629900 -2.46334400 3.39119900

O -4.89825300 -2.14978000 4.79989800

C -3.73003100 -2.52011100 5.55845500

C -2.50101900 -2.68161500 4.69547300

C -4.16018100 -1.29142700 0.16495200

C -5.08076200 -1.79255000 -0.75867400

C -5.73409600 -0.95971300 -1.72561700

C -5.48287500 0.39593700 -1.79935900

C -4.55312000 0.90609100 -0.88731300

C -3.87179500 0.10418100 0.08802400

C -5.58108700 -3.16338200 -0.96964900

C -6.49034300 -3.17766300 -1.97577100

C -6.64450200 -1.81698200 -2.50138300

N -4.11963700 2.21798400 -0.76085000

C -3.15684600 2.25955900 0.21998600

C -2.98687900 1.01453600 0.77315800

C -2.42328900 3.35668000 0.94862600

C -1.33236400 2.46088400 1.62942900

C -2.00610900 1.09386900 1.92492000

C -1.71555800 4.48503500 0.12292200

C -0.68256500 5.16391600 1.05676200

C -0.22582100 4.59964000 2.19849600

C -0.61001600 3.23806800 2.72117600

C -7.21509000 -4.31726300 -2.64494700

O -8.38943900 -3.82556300 -3.33622100

C -8.40018400 -2.60023100 -4.08767400

C -7.49390000 -1.54302400 -3.50459900

C -2.75690700 5.52568800 -0.44292600

C -2.61886300 6.94928300 0.11154700

C -1.19318300 7.42549200 -0.06265700

C -0.20605900 6.50480400 0.67471500

O -1.02316000 8.74877300 0.40406800

C 0.22774500 9.11119200 -0.19715000

C 1.37030600 8.36512200 0.50148600

C 1.02708200 7.00405600 0.93353800

C -4.01626800 -3.81253700 6.35204300

C -3.52800500 -1.35126700 6.54306400

C -6.28765100 -1.71957200 2.97192600

C -5.20161800 -3.97539700 3.17527900

C 3.63871400 -4.70344800 -1.06136200

C 4.92136900 -1.13882200 0.06005500

O 10.05696000 0.13143500 -2.20054100

C -6.27517200 -5.05278600 -3.62431600

C -7.78901000 -5.30999900 -1.62629000

C -8.04696300 -2.86465500 -5.56860000

C -9.86209400 -2.12238800 -3.98881400

C -3.39069200 3.95248400 2.01212700

C -0.94583800 3.92121500 -1.10259700

O -0.85236700 7.47377700 -1.46106200

O 2.47966800 8.86614100 0.62515700

C 0.00625400 8.62841000 -1.66733900

C -0.78909700 9.67490200 -2.45440700

C 1.26670500 8.19779800 -2.41480400

O 6.34266100 -4.03301500 -2.06094200

O 8.55047400 -3.98159500 -0.45953500

C 11.18467100 -0.67917800 0.37587600

C 10.59129600 0.58923600 1.00945800

C 11.82883800 -1.56904000 1.44146300

O 12.22298200 -0.35462100 -0.54337800

H 3.24717000 -1.33780700 -1.93072200

H -3.82485000 -0.80054500 2.79530100

H -0.58944400 2.24600700 0.85441500

H -1.63411400 -2.22271700 -1.02320500

H 0.36322800 -2.80628800 3.57421500

H 2.73266900 -3.04545600 2.08830300

H -3.89574300 -3.22171900 0.99200100

H 1.15280500 -1.84451600 -2.83357900

H 1.38045200 -3.59085600 -2.72673800

H 5.37972600 -0.94951100 -2.81502700

H 6.21756200 -2.18762400 -3.75866900

H 3.75010500 -2.11127000 -4.23878100

H 4.22428100 -3.68527000 -3.60027800

H 5.03437200 -3.45823800 1.57306900

H 5.82033400 -4.47465200 0.37933600

H 7.52875200 -3.55230800 1.89109100

H 6.95234000 -1.91725700 1.64573300

H 10.61622200 -2.35358100 -0.85696700

H 7.66023100 -0.59373900 -2.75979700

H -1.61011200 -3.06828100 5.18890600

H -5.97715700 1.03890800 -2.52432100

H -5.24636500 -4.03342400 -0.41644800

H -4.37340800 2.96815800 -1.38472900

H -1.27421200 0.28188300 1.95051100

H -2.51369700 1.09312800 2.90177100

H 0.46194700 5.17601100 2.81487700

H -1.22781000 3.34942600 3.62591700

H 0.29548100 2.70829500 3.05010900

H -7.58246000 -0.54225400 -3.92451400

H -2.63841800 5.58588100 -1.52905400

H -3.77819300 5.17815300 -0.26181600

H -2.86464100 6.98745200 1.17932200

H -3.28708800 7.63892700 -0.41359200

H 0.37200200 10.18895600 -0.11049900

H 1.80715300 6.42607200 1.42114600

H -4.92516900 -3.68444200 6.94935400

H -4.15728600 -4.66859400 5.68707400

H -3.18634200 -4.04286700 7.03098300

H -2.68355300 -1.54393900 7.21451500

H -3.33454100 -0.42569300 5.99280200

H -4.43110300 -1.21371900 7.14816700

H -7.14482100 -2.09433200 3.54149300

H -6.48799600 -1.85318200 1.90470200

H -6.18499400 -0.64905700 3.17546600

H -4.27857800 -4.53391300 3.35615300

H -5.97223500 -4.34402800 3.85967000

H -5.53401600 -4.18867100 2.15342400

H 2.68412300 -5.01222000 -1.49719300

H 4.45209200 -5.04882600 -1.69755000

H 3.71947900 -5.21061200 -0.09374100

H 4.38685200 -0.50087900 -0.64526600

H 4.36175100 -1.11395300 1.00172800

H 5.88878000 -0.66280800 0.23791500

H -6.84455700 -5.78585600 -4.20633400

H -5.48701200 -5.57620800 -3.07192300

H -5.78841500 -4.35724400 -4.31370300

H -6.98783800 -5.77829200 -1.04501000

H -8.46809100 -4.79615100 -0.94049700

H -8.34655200 -6.09575900 -2.14658000

H -7.00755200 -3.18115700 -5.68539100

H -8.19329200 -1.95871700 -6.16850100

H -8.69790900 -3.65125500 -5.96477200

H -10.52896500 -2.89623900 -4.38322700

H -10.12608400 -1.93624800 -2.94387900

H -10.01565000 -1.20215000 -4.56343700

H -4.31381800 4.31775300 1.55271700

H -3.68232600 3.18225300 2.73263600

H -2.93727000 4.78210900 2.56332200

H -1.63085500 3.40427800 -1.78449100

H -0.47895800 4.74554700 -1.65119400

H -0.15741200 3.21824000 -0.82187800

H -0.16114000 10.53933900 -2.69931400

H -1.64975800 10.01635600 -1.87210900

H -1.15319500 9.23686600 -3.38941000

H 1.97585100 9.02921600 -2.48783900

H 1.76358900 7.36476600 -1.91158700

H 1.00487500 7.87785900 -3.42872900

H 7.11551700 -4.36090100 -1.56361200

H 8.89575400 -4.45347600 0.31850800

H 11.37563100 1.11883700 1.55892700

H 9.77946800 0.34473400 1.70238700

H 10.20127500 1.26725400 0.24320300

H 12.63719300 -1.01587400 1.92982200

H 12.26298800 -2.46235300 0.97931600

H 11.10061800 -1.87576400 2.19681000

H 11.79422600 0.12349100 -1.27923600

-------------------------------------------------------------------------------------------

**Table S9.** The coordinates for the lowest-energy conformer of *ent*-**2a** in ECD calculation

-------------------------------------------------------------------------------------------

Coordinates (Angstroms)

X Y Z

-------------------------------------------------------------------------------------------

C -0.72655400 -2.73996400 1.43361600

C -0.19049600 -2.55548600 0.11464700

C 1.19551600 -2.35599900 -0.03767300

C 1.98504900 -2.32469600 1.09918600

C 1.43189000 -2.48186900 2.39653400

C 0.06520200 -2.69203100 2.58113700

N -2.09797100 -2.93851400 1.31315400

C -2.43583600 -2.83865100 -0.02188100

C -1.31052900 -2.62122000 -0.77641200

C 3.50761400 -2.21459900 1.17765300

C 3.76061400 -1.89060000 2.69009300

C 2.50124200 -2.37291600 3.39298500

C -3.65391900 -3.15801700 -0.86825900

C -3.17675200 -2.40619700 -2.16695100

C -1.64217500 -2.63429700 -2.25042300

C -5.06517400 -2.60136500 -0.43632500

C -6.07191100 -2.67149500 -1.67602300

C -5.49226100 -2.03208600 -2.95395500

C -4.12040200 -2.62607500 -3.34294300

C -5.70349300 -3.43700700 0.70279400

C -7.06377800 -2.88537200 1.15210300

C -8.06050500 -2.70591200 -0.00479100

C -7.41486200 -2.07315200 -1.24377500

O -9.11089400 -1.93778400 0.52122600

C -10.10999000 -1.48733700 -0.40439100

C -9.45202700 -0.71502500 -1.54709500

C -8.07555300 -1.10084900 -1.89467500

C 5.01604700 -2.46339600 3.39130400

O 4.89797600 -2.14965600 4.79994700

C 3.72972900 -2.51991100 5.55849900

C 2.50074900 -2.68152300 4.69549800

C 4.16005000 -1.29165800 0.16496800

C 5.08060100 -1.79283200 -0.75866700

C 5.73410800 -0.96000200 -1.72549800

C 5.48310400 0.39569500 -1.79911800

C 4.55331800 0.90588700 -0.88712400

C 3.87179500 0.10397900 0.08807500

C 5.58070400 -3.16373000 -0.96976500

C 6.49003700 -3.17804700 -1.97581900

C 6.64448100 -1.81732800 -2.50124000

N 4.11993500 2.21781100 -0.76063400

C 3.15697800 2.25940500 0.22003800

C 2.98684700 1.01435900 0.77312300

C 2.42331400 3.35653000 0.94856700

C 1.33223500 2.46075200 1.62913400

C 2.00583600 1.09366900 1.92467900

C 1.71577200 4.48494600 0.12279000

C 0.68269700 5.16385300 1.05652200

C 0.22573000 4.59954800 2.19815200

C 0.60973200 3.23790500 2.72080200

C 7.21458700 -4.31766500 -2.64516100

O 8.38924100 -3.82612400 -3.33602700

C 8.40045400 -2.60061800 -4.08719400

C 7.49411700 -1.54338900 -3.50426000

C 2.75728600 5.52553700 -0.44285900

C 2.61932300 6.94910600 0.11170600

C 1.19372600 7.42547400 -0.06264400

C 0.20639600 6.50482800 0.67450200

O 1.02379100 8.74873900 0.40419900

C -0.22698300 9.11135500 -0.19717700

C -1.36973000 8.36533600 0.50121600

C -1.02674000 7.00417500 0.93316500

C 6.28747300 -1.71976600 2.97199600

C -3.63898300 -4.70345700 -1.06123100

C -4.92153500 -1.13865600 0.05977900

O -10.05725800 0.13113300 -2.20105100

C 6.27467200 -5.05253600 -3.62502300

C 7.78794900 -5.31091700 -1.62670000

C 8.04779200 -2.86473000 -5.56830700

C 9.86239000 -2.12298300 -3.98765600

C 3.39050300 3.95226100 2.01228400

C 0.94618300 3.92126100 -1.10287400

O 0.85308900 7.47391900 -1.46107900

O -2.47906800 8.86644900 0.62474100

C -0.00531800 8.62870200 -1.66737200

O -6.34295500 -4.03308400 -2.06079200

C -11.18447800 -0.67830600 0.37595100

C -11.82850200 -1.56770200 1.44201300

C -10.59070900 0.59021400 1.00896400

O -12.22297200 -0.35382600 -0.54310300

H 0.58943600 2.24593900 0.85398500

H -3.24736300 -1.33789300 -1.93085300

H 3.82463700 -0.80062800 2.79517900

C 0.79030800 9.67517200 -2.45421100

C -1.26569000 8.19834300 -2.41509700

C 4.01599700 -3.81227000 6.35217100

C 3.52764700 -1.35098900 6.54300800

C 5.20128100 -3.97550000 3.17567600

H 3.89555600 -3.22192900 0.99203700

O -8.55077100 -3.98136800 -0.45923600

H 1.63393300 -2.22301600 -1.02322200

H -0.36346700 -2.80625000 3.57421500

H -2.73296200 -3.04477300 2.08834500

H -1.15298200 -1.84471000 -2.83363800

H -1.38067300 -3.59104500 -2.72665100

H -6.21777800 -2.18795100 -3.75872700

H -5.37998700 -0.94967300 -2.81527100

H -4.22446700 -3.68548900 -3.60023400

H -3.75031400 -2.11152300 -4.23882500

H -5.82054800 -4.47445200 0.37961900

H -5.03451500 -3.45785700 1.57312900

H -7.52891100 -3.55185200 1.89127400

H -6.95242300 -1.91686600 1.64571000

H -10.61666000 -2.35318300 -0.85656900

H -7.66046100 -0.59402500 -2.76011500

H 1.60983200 -3.06816000 5.18893500

H 5.97756000 1.03866700 -2.52395700

H 5.24575800 -4.03377700 -0.41671100

H 4.37382800 2.96798400 -1.38446900

H 1.27388300 0.28173000 1.95004100

H 2.51322100 1.09281400 2.90163500

H -0.46206100 5.17595200 2.81447300

H 1.22734700 3.34913500 3.62568100

H -0.29586600 2.70815400 3.04949000

H 7.58291500 -0.54258300 -3.92403700

H 3.77851600 5.17787200 -0.26167100

H 2.63892400 5.58582200 -1.52899700

H 3.28769700 7.63871100 -0.41329900

H 2.86497900 6.98716400 1.17951000

H -0.37115300 10.18912100 -0.11043100

H -1.80695100 6.42622900 1.42058800

H 7.14456100 -2.09440800 3.54176900

H 6.48795500 -1.85365100 1.90483600

H 6.18483400 -0.64919400 3.17525600

H -2.68433500 -5.01233100 -1.49687000

H -3.71995500 -5.21054300 -0.09358800

H -4.45224600 -5.04884100 -1.69756500

H -4.38681100 -0.50088600 -0.64554200

H -5.88894300 -0.66252400 0.23734800

H -4.36212200 -1.11367600 1.00157200

H 6.84398400 -5.78550700 -4.20723700

H 5.48624700 -5.57597300 -3.07302400

H 5.78826000 -4.35656100 -4.31422200

H 8.46700300 -4.79750500 -0.94055100

H 6.98649500 -5.77918100 -1.04578800

H 8.34540500 -6.09666200 -2.14710600

H 8.19442100 -1.95870100 -6.16799600

H 8.69881900 -3.65132000 -5.96436300

H 7.00839900 -3.18113100 -5.68555900

H 10.52932500 -2.89684100 -4.38194900

H 10.01630300 -1.20265100 -4.56203100

H 10.12598900 -1.93708800 -2.94257800

H 4.31374400 4.31753400 1.55310400

H 2.93698100 4.78187100 2.56341900

H 3.68195500 3.18199200 2.73282500

H 0.15754000 3.21848100 -0.82230300

H 0.47960700 4.74568700 -1.65159200

H 1.63122700 3.40416200 -1.78461600

H -7.11566900 -4.36095400 -1.56323300

H -12.63663500 -1.01423200 1.93039700

H -12.26293300 -2.46109500 0.98028700

H -11.10013300 -1.87429200 2.19727000

H -11.37485200 1.12017600 1.55835900

H -10.20070200 1.26787700 0.24238700

H -9.77880500 0.34584000 1.70185100

H -11.79433700 0.12382900 -1.27932800

H 1.15445700 9.23718100 -3.38921500

H 0.16252300 10.53973700 -2.69910400

H 1.65096200 10.01644200 -1.87179200

H -1.76277500 7.36532000 -1.91205800

H -1.97470800 9.02986500 -2.48817000

H -1.00372300 7.87846800 -3.42900600

H 3.18604400 -4.04262200 7.03106900

H 4.92485700 -3.68409100 6.94952800

H 4.15712000 -4.66835300 5.68725000

H 3.33417300 -0.42545800 5.99267400

H 2.68318700 -1.54362400 7.21445900

H 4.43072900 -1.21335700 7.14812100

H 4.27817400 -4.53391800 3.35653000

H 5.53380000 -4.18898100 2.15390700

H 5.97177000 -4.34407700 3.86024300

H -8.89594800 -4.45313200 0.31892100

-------------------------------------------------------------------------------------------

**Table S10.** The coordinates for the lowest-energy conformer of **2b** in ECD calculation

-------------------------------------------------------------------------------------------

Coordinates (Angstroms)

X Y Z

-------------------------------------------------------------------------------------------

C 2.02758200 0.54840200 1.49426600

C 1.65161400 1.09574100 0.22140200

C 0.46084000 1.84113500 0.12306500

C -0.29929200 2.02213300 1.26629400

C 0.09420500 1.48096500 2.51677000

C 1.26756700 0.73767400 2.64838100

N 3.21499300 -0.15600000 1.32214900

C 3.61446500 -0.01893300 0.00704100

C 2.68712000 0.71236300 -0.69144900

C -1.57102700 2.85580400 1.41597300

C -2.11629600 2.42206300 2.82121500

C -0.90899700 1.82631000 3.52821900

C 4.64790800 -0.66710200 -0.89597000

C 4.54027200 0.37340200 -2.07309500

C 3.04004900 0.76621300 -2.15915400

C 6.14925100 -0.75474000 -0.41896000

C 7.09426300 -1.04037300 -1.67714600

C 6.84638500 -0.06855700 -2.84906400

C 5.36600100 -0.03558800 -3.28623200

C 6.38093900 -1.91553200 0.58187400

C 7.83365800 -1.99642100 1.07269000

C 8.86256700 -2.05590000 -0.06888600

C 8.54833600 -1.06257800 -1.19426700

O 10.10749400 -1.82637700 0.53729300

C 11.23367800 -1.66569500 -0.33641700

C 10.95702300 -0.54889100 -1.34223900

C 9.54998500 -0.33311400 -1.71408800

C -2.80207100 3.47094400 3.73030500

O -3.05047900 2.83038300 5.00519500

C -1.99272300 2.18914000 5.74507000

C -0.85022100 1.74414200 4.86317900

C -2.55351500 2.77246500 0.26268900

C -2.92047100 3.91954400 -0.44425400

C -3.87299700 3.89268300 -1.51547400

C -4.47819200 2.71754700 -1.91530400

C -4.09199200 1.55708700 -1.23574600

C -3.14749100 1.54495000 -0.15628400

C -2.48157800 5.31896500 -0.29785400

C -3.12240600 6.10459500 -1.19870800

C -4.02446200 5.27309800 -2.00344700

N -4.51161800 0.25523600 -1.46784700

C -3.89650300 -0.56567400 -0.55182100

C -3.05339500 0.16421800 0.24990300

C -3.75800000 -2.05814500 -0.38376000

C -3.14332300 -2.04751900 1.05694800

C -2.27051200 -0.76812300 1.15216900

C -5.04422200 -2.95334500 -0.38748700

C -4.66968200 -4.30811200 0.26352800

C -3.57446200 -4.48538700 1.03772400

C -2.57857300 -3.41625700 1.41094100

C -2.96496300 7.56621500 -1.53082300

O -4.13729400 8.04980600 -2.23068700

C -4.81887700 7.28605000 -3.23968700

C -4.78623600 5.80015600 -2.97553700

C -5.60739400 -3.14942700 -1.84739300

C -5.55139600 -4.58854500 -2.37660700

C -6.22961700 -5.51495300 -1.39087600

C -5.55020900 -5.45686200 -0.01228100

O -6.22996200 -6.85361200 -1.84506800

C -7.22570100 -7.44748000 -0.99987800

C -6.67968600 -7.57957200 0.42657100

C -5.79603300 -6.48179900 0.83943700

C -2.66779700 0.96197200 6.38888100

C -1.48997200 3.13697200 6.85422800

C -1.97932300 4.75878500 3.90940600

C -4.20807200 3.81828800 3.23269000

C 4.05167600 -2.04989600 -1.29419300

C 6.55739900 0.57194900 0.27345400

O 11.85948700 0.08910700 -1.87936900

C -2.91625200 8.43962300 -0.27067600

C -1.69335000 7.79344300 -2.37649200

C -6.27439600 7.78497900 -3.15677500

C -4.24957800 7.60047600 -4.64185200

C -2.71327500 -2.56174200 -1.42128900

C -6.19510600 -2.32945700 0.44754400

O -7.61907000 -5.16290500 -1.25733400

O -7.01138400 -8.50557300 1.15431900

C -8.37586600 -6.39454000 -1.10524700

C -9.18238700 -6.61744000 -2.38905800

C -9.28484800 -6.28803100 0.11759700

O 6.83702700 -2.33941700 -2.24461700

O 8.84607000 -3.34728200 -0.70532200

C 12.50660300 -1.44505300 0.52934700

C 12.41211700 -0.15150300 1.35406100

C 12.73030000 -2.65546300 1.43825800

O 13.62211300 -1.40822900 -0.35536000

H 5.00689700 1.29083600 -1.69622000

H -2.85872100 1.63036300 2.66113300

H -3.98007300 -1.86854800 1.74036600

H 0.14521300 2.26257500 -0.82783600

H 1.56366800 0.30706100 3.60224300

H 3.73928800 -0.58541300 2.06794800

H -1.25117600 3.90054000 1.49423900

H 2.91215700 1.75227800 -2.62190100

H 2.44892700 0.05494400 -2.75532400

H 7.15255300 0.94685800 -2.56847500

H 7.48363600 -0.38697900 -3.68019700

H 5.25027000 0.69403700 -4.09783500

H 5.06473300 -1.01013500 -3.68506700

H 5.72738600 -1.79302100 1.45551100

H 6.10043800 -2.86427400 0.11756100

H 7.98293100 -2.88515400 1.70097700

H 8.08548100 -1.13722000 1.69914100

H 11.38886800 -2.58652500 -0.91859000

H 9.38750400 0.40719700 -2.49067500

H 0.03275800 1.35883800 5.37155000

H -5.20398700 2.68530300 -2.72492700

H -1.73436300 5.65879000 0.41031200

H -5.23069400 -0.00808100 -2.12351600

H -2.17074900 -0.43398800 2.18917700

H -1.24876100 -0.92770900 0.77911100

H -3.36867000 -5.48703600 1.41142000

H -1.61970000 -3.61108700 0.90550100

H -2.35946900 -3.48602600 2.48616200

H -5.42584600 5.18063600 -3.60226800

H -6.65599400 -2.83717100 -1.86422400

H -5.07900900 -2.49737700 -2.54929000

H -4.51716000 -4.92627300 -2.51105000

H -6.06116300 -4.66908300 -3.34177200

H -7.49585300 -8.42846900 -1.39304000

H -5.39163900 -6.52088000 1.84694500

H -3.03865800 0.28712100 5.61179200

H -1.96397200 0.41321400 7.02501000

H -3.51577000 1.28386200 7.00368200

H -1.00285800 4.02095300 6.43460800

H -2.33454300 3.46570200 7.46904700

H -0.76797200 2.62660800 7.50304700

H -0.93381700 4.55015200 4.15463900

H -2.41503400 5.35410700 4.71801000

H -2.00041400 5.36806000 2.99912600

H -4.69604300 4.49936300 3.93818800

H -4.17106200 4.29670300 2.24946600

H -4.81810900 2.91285500 3.15593300

H 3.06867800 -1.90993300 -1.75326200

H 4.69576200 -2.59474900 -1.98272800

H 3.89725600 -2.67000800 -0.40424900

H 6.01107400 0.68908300 1.21580100

H 7.62511000 0.61110900 0.50294800

H 6.33843300 1.45252200 -0.33237500

H -2.84350400 9.49497600 -0.55340400

H -2.04942200 8.18432900 0.34773900

H -3.82491900 8.29841000 0.32073300

H -1.65742700 7.12265300 -3.23949900

H -1.66443900 8.82895400 -2.73306100

H -0.79905800 7.60307200 -1.77285800

H -6.68808900 7.57423100 -2.16653100

H -6.90051400 7.29983200 -3.91387700

H -6.30174700 8.86718400 -3.32176100

H -4.25028100 8.68385200 -4.80202700

H -3.22681800 7.23238000 -4.75477200

H -4.86308300 7.13411800 -5.42165100

H -3.00973300 -2.30991900 -2.44361700

H -1.74455000 -2.08321600 -1.24841300

H -2.57303600 -3.64582300 -1.36855200

H -7.07085000 -2.98514100 0.40917900

H -5.92942000 -2.19004300 1.49846400

H -6.48318800 -1.35398500 0.03907200

H -8.51253100 -6.75960600 -3.24202400

H -9.81464900 -5.74531500 -2.58483400

H -9.82832300 -7.49814300 -2.29559000

H -9.78319900 -7.24375600 0.31200800

H -8.72334800 -6.00534900 1.01127100

H -10.05366200 -5.52823700 -0.05747300

H 7.40833300 -2.99999900 -1.80947700

H 8.95937100 -4.01585800 -0.00717100

H 11.54317700 -0.16811900 2.02017000

H 12.33349800 0.72587200 0.70353900

H 13.31764200 -0.04163800 1.95875400

H 11.91398600 -2.77085500 2.15614200

H 13.66945400 -2.52430400 1.98478100

H 12.81323900 -3.56959800 0.84034400

H 13.42931600 -0.70579600 -1.00601100

-------------------------------------------------------------------------------------------

**Table S11.** The coordinates for the lowest-energy conformer of *ent*-**2b** in ECD calculation

-------------------------------------------------------------------------------------------

Coordinates (Angstroms)

X Y Z

-------------------------------------------------------------------------------------------

C -2.02759700 0.54836300 1.49425000

C -1.65169100 1.09581100 0.22142800

C -0.46093100 1.84123400 0.12309700

C 0.29924000 2.02216600 1.26631000

C -0.09422600 1.48091700 2.51676100

C -1.26755600 0.73757600 2.64836200

N -3.21498400 -0.15608400 1.32211600

C -3.61453100 -0.01887800 0.00704500

C -2.68722500 0.71250000 -0.69141500

C 1.57097500 2.85585000 1.41599900

C 2.11625200 2.42208200 2.82121900

C 0.90897700 1.82624700 3.52820700

C -4.64790900 -0.66709200 -0.89599200

C -4.54032800 0.37344200 -2.07309300

C -3.04013100 0.76637200 -2.15911800

C -6.14926900 -0.75481900 -0.41903500

C -7.09428800 -1.04046400 -1.67717100

C -6.84641100 -0.06866400 -2.84911700

C -5.36600500 -0.03562800 -3.28623500

C -6.38093800 -1.91556500 0.58185200

C -7.83364200 -1.99638900 1.07270400

C -8.86256400 -2.05594000 -0.06885500

C -8.54836000 -1.06268300 -1.19428400

O -10.10749800 -1.82642500 0.53730500

C -11.23367900 -1.66579600 -0.33640400

C -10.95704700 -0.54914100 -1.34238900

C -9.55000900 -0.33328500 -1.71419300

C 2.80197400 3.47095400 3.73035100

O 3.05043500 2.83035000 5.00520700

C 1.99269100 2.18906500 5.74506400

C 0.85021000 1.74402800 4.86316500

C 2.55345100 2.77247600 0.26271500

C 2.92041300 3.91953500 -0.44426300

C 3.87287300 3.89262700 -1.51553500

C 4.47800800 2.71747000 -1.91538900

C 4.09180700 1.55703800 -1.23578600

C 3.14738200 1.54493400 -0.15626100

C 2.48157300 5.31897800 -0.29785700

C 3.12240000 6.10457100 -1.19874300

C 4.02438400 5.27302800 -2.00350200

N 4.51143300 0.25518300 -1.46785400

C 3.89632200 -0.56570400 -0.55182600

C 3.05328900 0.16421400 0.24995700

C 3.75794900 -2.05816500 -0.38363500

C 3.14345600 -2.04745900 1.05715800

C 2.27057900 -0.76810100 1.15239900

C 5.04424700 -2.95325900 -0.38742300

C 4.66989000 -4.30798400 0.26379400

C 3.57477000 -4.48528500 1.03812500

C 2.57882900 -3.41619600 1.41134500

C 2.96500500 7.56619100 -1.53086800

O 4.13729500 8.04977200 -2.23080000

C 4.81889000 7.28594700 -3.23974600

C 4.78615300 5.80005800 -2.97561000

C 5.60722700 -3.14950200 -1.84736900

C 5.55106000 -4.58866500 -2.37646900

C 6.22952900 -5.51494500 -1.39079000

C 5.55046800 -5.45669100 -0.01203300

O 6.22976900 -6.85368100 -1.84479600

C 7.22577300 -7.44738500 -0.99980500

C 6.68020800 -7.57923500 0.42685200

C 5.79659000 -6.48145400 0.83980500

C 4.20795200 3.81840600 3.23273500

C -4.05153300 -2.04981500 -1.29421600

C -6.55747900 0.57185700 0.27338100

O -11.85955300 0.08862700 -1.87972200

C 2.91634900 8.43960500 -0.27072200

C 1.69337600 7.79346700 -2.37649600

C 6.27444500 7.78476300 -3.15676500

C 4.24968300 7.60044400 -4.64192800

C 2.71314300 -2.56193600 -1.42098700

C 6.19517900 -2.32915300 0.44736700

O 7.61901000 -5.16286000 -1.25764100

O 7.01213400 -8.50512100 1.15464600

C 8.37586300 -6.39445900 -1.10571100

O -6.83716700 -2.33952500 -2.24465400

C -12.50658900 -1.44496600 0.52935800

C -12.73024500 -2.65520500 1.43851900

C -12.41205700 -0.15127000 1.35384500

O -13.62215200 -1.40828000 -0.35527600

H 3.98028300 -1.86840400 1.74046500

H -5.00703900 1.29084800 -1.69624100

H 2.85869900 1.63040200 2.66113500

C 9.18192800 -6.61748900 -2.38979900

C 9.28532900 -6.28780600 0.11676600

C 1.48991200 3.13684500 6.85425700

C 2.66779500 0.96191300 6.38886700

C 1.97917900 4.75875900 3.90950400

H 1.25109700 3.90057800 1.49426000

O -8.84599700 -3.34736300 -0.70522300

H -0.14535400 2.26274800 -0.82778700

H -1.56362100 0.30688900 3.60219900

H -3.73937500 -0.58531600 2.06795700

H -2.91230500 1.75246700 -2.62181600

H -2.44893900 0.05516100 -2.75528600

H -7.48359800 -0.38713600 -3.68027800

H -7.15264100 0.94674600 -2.56857400

H -5.06467400 -1.01016900 -3.68503500

H -5.25030200 0.69398600 -4.09785200

H -6.10045100 -2.86435300 0.11760700

H -5.72736400 -1.79300900 1.45546200

H -7.98290200 -2.88506600 1.70108000

H -8.08541900 -1.13714000 1.69911400

H -11.38893600 -2.58668700 -0.91846000

H -9.38753300 0.40696500 -2.49083900

H -0.03274800 1.35865900 5.37152100

H 5.20374800 2.68518600 -2.72505900

H 1.73437100 5.65882400 0.41030700

H 5.23021000 -0.00821600 -2.12381700

H 2.17095300 -0.43388400 2.18939200

H 1.24879000 -0.92778100 0.77948300

H 3.36913000 -5.48691100 1.41196200

H 1.61993400 -3.61113500 0.90599700

H 2.35982700 -3.48586800 2.48659300

H 5.42570600 5.18051400 -3.60237500

H 5.07884100 -2.49743200 -2.54924900

H 6.65585800 -2.83735300 -1.86436100

H 6.06059600 -4.66928900 -3.34174900

H 4.51679400 -4.92639400 -2.51063600

H 7.49581200 -8.42844500 -1.39286300

H 5.39244600 -6.52039700 1.84741500

H 4.17090500 4.29686400 2.24953500

H 4.81804700 2.91301400 3.15593500

H 4.69590400 4.49947700 3.93825200

H -3.06847300 -1.90978300 -1.75314500

H -3.89718900 -2.66996800 -0.40428500

H -4.69545000 -2.59467500 -1.98290400

H -7.62522400 0.61101500 0.50274000

H -6.01128800 0.68892700 1.21581400

H -6.33842100 1.45245300 -0.33237900

H 2.84373400 9.49497500 -0.55342500

H 2.04947300 8.18440600 0.34766400

H 3.82498100 8.29829400 0.32072300

H 1.66449100 8.82897500 -2.73307700

H 1.65742500 7.12268000 -3.23950300

H 0.79908600 7.60311900 -1.77285300

H 6.68806800 7.57397700 -2.16649800

H 6.90056700 7.29957400 -3.91383400

H 6.30191400 8.86697000 -3.32173400

H 4.25045000 8.68382700 -4.80205000

H 4.86319300 7.13407900 -5.42171900

H 3.22690700 7.23241200 -4.75491800

H 1.74440800 -2.08343500 -1.24808900

H 3.00950000 -2.31024000 -2.44337800

H 2.57294900 -3.64601700 -1.36810300

H 7.07094700 -2.98481300 0.40909200

H 6.48321400 -1.35377400 0.03864700

H 5.92958300 -2.18948700 1.49827700

H -7.40834700 -3.00009500 -1.80933100

H -12.81315800 -3.56946700 0.84079400

H -11.91392700 -2.77043200 2.15642500

H -13.66940300 -2.52397700 1.98502100

H -12.33360200 0.72599900 0.70316000

H -11.54300900 -0.16771400 2.01981800

H -13.31748800 -0.04134100 1.95866600

H -13.42939500 -0.70598800 -1.00609000

H 9.81409200 -5.74536800 -2.58589900

H 9.82792900 -7.49816000 -2.29646400

H 8.51179600 -6.75975900 -3.24253600

H 8.72420600 -6.00494800 1.01062500

H 9.78371900 -7.24351800 0.31113200

H 10.05409800 -5.52806400 -0.05872700

H 1.00273700 4.02082400 6.43470200

H 0.76796100 2.62642400 7.50308500

H 2.33448800 3.46560100 7.46905400

H 3.51575300 1.28380300 7.00369200

H 1.96398100 0.41311700 7.02497400

H 3.03870500 0.28708200 5.61178200

H 2.00029700 5.36809200 2.99926600

H 0.93366400 4.55008900 4.15466700

H 2.41482500 5.35403700 4.71817400

H -8.95929100 -4.01586800 -0.00700500

-------------------------------------------------------------------------------------------

**Table S12.** The coordinates for the lowest-energy conformer of **3** in ECD calculation

-------------------------------------------------------------------------------------------

Coordinates (Angstroms)

X Y Z

-------------------------------------------------------------------------------------------

C -4.66674700 0.19170900 0.06028800

C -4.77171800 -1.15083800 -0.42067700

C -3.45190600 0.72897900 0.44832300

C -2.33522600 -0.10844800 0.35863400

C -2.41110900 -1.44889000 -0.13447700

C -3.66094000 -1.97059100 -0.52430600

N -1.00961200 0.15090900 0.69189200

C 1.14214800 -1.45268100 0.68739200

C 1.14794300 -2.65885200 -0.31801200

C -0.29418100 -3.22930100 -0.31578800

C 2.37735200 -0.52986700 0.36054000

C 3.70777700 -1.37935200 0.36777200

C 3.61920300 -2.67961000 -0.46137000

C 2.37818900 -3.53496000 -0.13880200

C 2.56082200 0.60766600 1.39663300

C 3.84824100 1.35801600 1.22418700

C 4.88995800 -0.48003200 -0.02156300

O 5.97245300 1.67725500 0.29597600

C 7.26315400 1.08983200 0.08523900

C 5.93778700 -0.89435200 -0.77367600

C 2.19287100 0.12328900 -1.03585600

C 8.23572600 2.20904300 -0.38081400

C 7.17071600 -0.11665100 -0.85524500

O 8.11429500 -0.46827700 -1.55269400

C 1.09122500 -1.93170200 2.16697400

O 9.54565600 1.64036500 -0.43842300

C 8.30968100 3.30729100 0.67781300

C 4.90300600 0.85593700 0.56063300

C -1.07035300 -1.95190500 -0.10863700

C -0.25764300 -0.96121400 0.37627000

C -6.18250200 -1.40152600 -0.73824600

C -6.91277800 -0.28920600 -0.48591900

C -6.02219300 0.76763000 0.01345600

C -8.39718300 -0.04110800 -0.53647500

O -8.65651100 1.38281400 -0.65331000

C -7.92373300 2.37557200 0.08897800

C -6.48392200 1.98855000 0.31596300

C -9.03917100 -0.63035400 -1.79516300

C -9.08131200 -0.61876700 0.71927000

C -7.97624600 3.62368000 -0.80914300

C -8.62179000 2.68549900 1.43033300

C 7.82647200 2.78862700 -1.74104100

O 3.97601600 -1.74090500 1.74754100

H 1.23511700 -2.20969900 -1.31192800

H -3.36629600 1.74879300 0.81420100

H -3.74391800 -2.98705900 -0.89785600

H -0.66321200 1.03812200 1.01579300

H -0.51757100 -3.74942300 -1.25286000

H -0.46383200 -3.95050200 0.49507400

H 3.61427200 -2.43688700 -1.52868600

H 4.52952700 -3.26340700 -0.28729400

H 2.44185300 -3.94268500 0.87410600

H 2.35231300 -4.39125000 -0.82152700

H 1.72809900 1.31793900 1.31257900

H 2.51708000 0.20654100 2.41252600

H 3.92027300 2.37083000 1.60923900

H 7.65007900 0.70916800 1.04334800

H 5.94852700 -1.84587800 -1.29179300

H 1.18490200 0.53207900 -1.12905700

H 2.89553000 0.94166700 -1.19073700

H 2.32994100 -0.57394400 -1.86168800

H 2.03832100 -2.32990500 2.51939400

H 0.80703400 -1.10939000 2.82808100

H 0.32155500 -2.69766100 2.28091800

H 9.51644300 0.89207800 -1.05513000

H 9.06871700 4.03518000 0.38340600

H 7.35387200 3.81977300 0.79065200

H 8.60278700 2.88979900 1.64436100

H -6.55871100 -2.35096400 -1.10153400

H -5.83302800 2.76258800 0.71607500

H -8.91795600 -1.71602700 -1.82260500

H -8.58167000 -0.20585500 -2.68989700

H -10.10781600 -0.40328700 -1.81059100

H -9.03988000 -1.71102800 0.70242700

H -8.59279400 -0.28851000 1.63716500

H -10.12963200 -0.30992000 0.74901300

H -9.01673400 3.89319800 -1.00527600

H -7.48632000 3.42522900 -1.76388800

H -7.48301900 4.47428400 -0.33047000

H -8.14120800 3.53305800 1.92808800

H -9.66793500 2.94401500 1.24891900

H -8.58864700 1.83665500 2.11334200

H 7.84959600 2.02820900 -2.52514100

H 6.82061400 3.21346100 -1.70322800

H 8.52639900 3.57865000 -2.02159700

H 4.84962400 -2.15109000 1.78719500

-------------------------------------------------------------------------------------------

**Table S13.** The coordinates for the lowest-energy conformer of *ent*-**3** in ECD calculation

-------------------------------------------------------------------------------------------

Coordinates (Angstroms)

X Y Z

-------------------------------------------------------------------------------------------

C 2.33170100 -0.12281200 0.36869900

C 2.41187100 -1.46567000 -0.12473100

C 3.66410600 -1.98231700 -0.51919300

C 4.77273600 -1.15656700 -0.41900200

C 4.66260100 0.18770800 0.06358300

C 3.44579900 0.72146700 0.45598800

N 1.00660000 0.13223900 0.70589400

C 0.25717900 -0.98334500 0.39458500

C 1.07254700 -1.97385400 -0.09301800

C 6.18449400 -1.40043900 -0.73956400

C 6.91072000 -0.28231100 -0.48984000

C 6.01519000 0.76922700 0.01429500

C -1.14646700 -1.47243500 0.69406400

C -1.14842300 -2.66874600 -0.32616400

C 0.29306900 -3.24815600 -0.31498900

C -2.37682600 -0.53847400 0.37271100

C -3.71023700 -1.38491200 0.35351000

C -3.61931700 -2.67166800 -0.49909000

C -2.38746200 -3.54139500 -0.17065300

C -2.56573900 0.58072500 1.43162500

C -3.85259400 1.33729200 1.26442500

C -4.90926100 0.84625300 0.58844900

C -4.89136900 -0.47803800 -0.02512700

O -5.98241300 1.67077600 0.35410900

C -7.27134900 1.09703200 0.09806200

C -7.17227000 -0.09682000 -0.85756300

C -5.94015100 -0.87768700 -0.78821600

C 8.39424900 -0.02409500 -0.55017500

O 8.65289700 1.39821300 -0.64597400

C 7.91490800 2.38136600 0.09857100

C 6.47439800 1.99166300 0.32554700

C 9.09578400 -0.62073500 0.68880400

C -1.10895300 -1.96939700 2.17010000

C -2.17548200 0.14424800 -1.00787400

O -8.11807600 -0.44167400 -1.56664700

O -3.99066900 -1.76884400 1.72147100

C -8.21658700 2.23081500 -0.39374300

C -8.31160900 3.32650300 0.66996400

C -7.74743400 2.81204400 -1.73713000

O -9.52354600 1.67530700 -0.50277400

H -1.22345200 -2.20811300 -1.31821500

C 8.61391900 2.68705400 1.44260200

C 7.96396900 3.63379300 -0.79822600

C 9.02312900 -0.58943300 -1.82986800

H 3.75038300 -2.99981200 -0.89247100

H 3.35656100 1.74173700 0.82314900

H 0.65622400 1.02036000 1.02911400

H 6.56479900 -2.34921800 -1.10418300

H 0.52145100 -3.76453900 -1.25541100

H 0.44907400 -3.97703300 0.49465300

H -4.53900700 -3.25108000 -0.34547800

H -3.59681100 -2.40956700 -1.56389500

H -2.46866000 -3.95555800 0.84074100

H -2.35844500 -4.39203100 -0.86321600

H -2.53211300 0.15628700 2.44107200

H -1.72794400 1.29063000 1.36751500

H -3.92941700 2.34080200 1.67486500

H -7.69745600 0.71246200 1.03986000

H -5.94961200 -1.81833500 -1.32827900

H 5.82154300 2.76245500 0.73221900

H 10.14598300 -0.30921800 0.70670100

H 9.05254800 -1.71503600 0.65828900

H 8.61631400 -0.29733300 1.61696300

H -0.35315300 -2.75349700 2.27595700

H -0.80978200 -1.15596900 2.83957400

H -2.06980800 -2.35247900 2.51079100

H -1.17243900 0.57731000 -1.06533000

H -2.27671200 -0.54511300 -1.84898000

H -2.89579700 0.95032200 -1.16163900

H -4.85190200 -2.21820100 1.71751100

H -9.04461500 4.07282500 0.34778700

H -8.65378100 2.90506600 1.62127600

H -7.34719700 3.81658000 0.82510900

H -8.42715300 3.61470000 -2.03978000

H -7.75687200 2.04872900 -2.52220900

H -6.73387300 3.21930900 -1.66100000

H -9.44016400 0.91109300 -1.10560300

H 8.13535300 3.53742400 1.94250500

H 9.66376500 2.93925700 1.25947300

H 8.57412600 1.83234900 2.12240700

H 9.00677300 3.90646800 -0.99133200

H 7.47589000 3.42945300 -1.75544600

H 7.46353800 4.48215000 -0.31810500

H 8.54676700 -0.14934700 -2.71015700

H 10.09178500 -0.35213800 -1.85520200

H 8.90651400 -1.67736000 -1.87284300

-------------------------------------------------------------------------------------------
